# Genome-wide identification and characterization of phased small interfering RNA genes in response to *Botrytis cinerea* infection in *Solanum lycopersicum*

**DOI:** 10.1038/s41598-017-02233-x

**Published:** 2017-06-08

**Authors:** Fangli Wu, Yue Chen, Xing Tian, Xiaole Zhu, Weibo Jin

**Affiliations:** 1 Zhejiang Province Key Laboratory of Plant Secondary Metabolism and Regulation, College of Life Sciences, Zhejiang Sci-Tech University, Hangzhou, 310018 China; 2 College of Life Sciences, Northwest A&F University, Yangling, 712100 China

## Abstract

Phased small interfering RNAs (phasiRNAs) are encoded by a novel class of genes known as phasiRNA producing (*PHAS*) genes. These genes play important regulatory roles by targeting protein coding transcripts in plant species. In this study, 91 regions were identified as potential *PHAS* loci in tomato, with additional evidence that seven of them can be triggered by five miRNAs. Among the identified loci, 51 were located in genic regions, and the remaining 40 were located in intergenic regions. The transient overexpression of *PHAS15* and *PHAS26* demonstrated that phasiRNAs predicted by PhaseTank were indeed generated from their respective *PHAS* loci. Using sRNA-seq data from *B. cinerea*-infected tomato leaves, we identified 50 *B. cinerea*-responsive phasiRNAs with increased abundance and five with decreased abundance. Moreover, 164 targets of these differentially expressed phasiRNAs were predicted, and 94 of them were confirmed experimentally using degradome data. Gene ontology analysis of the targets revealed an enrichment of genes with functions related to defense responses and signaling regulation. These results suggest that a large number of endogenous siRNAs, such as phasiRNAs, have not yet been identified in tomato and underscore the urgent need to systematically identify and functionally analyze siRNAs in tomato.

## Introduction

Endogenous small RNAs in plants regulate gene expression from DNA modifications to RNA stability at the transcriptional and post-transcriptional levels. These small RNAs include microRNAs (miRNAs); heterochromatic small-interfering RNAs; phased, secondary, small-interfering RNAs (phasiRNAs); and small-interfering RNAs from natural antisense transcripts. In addition to the numerous miRNA studies reported thus far, there has been increasing interest in the identification of loci that generate phasiRNAs. The first of these to be described were the trans-acting small-interfering RNAs (tasiRNAs); thus, tasiRNAs are phasiRNAs that have been shown to target a transcript in *trans*. Transcripts from these loci are converted into double-stranded RNA (dsRNA) by RNA-dependent RNA polymerase 6 (RDR6)-SGS3 and processed by DCL4, generating siRNAs in a 21-nt phase^1–5^. These phased siRNAs are known as phasiRNAs, and the loci that produce them were designated as *PHAS* genes by Zhai *et al*.^6^.

Similar to miRNAs, tasiRNAs repress their target transcripts at the post-transcriptional level. As indicated above, the primary transcripts from tasiRNA loci are used to generate dsRNAs by RDR6, and the dsRNAs are cleaved by DCL4 into phased 21-nt segments^3–5^. Four tasiRNA gene families, *TAS1*, *TAS2*, *TAS3*, and *TAS4*, have been identified in *Arabidopsis thaliana* ^3, 5, 7^. *TAS3* is conserved in various plant species^8^. Aside from these four tasiRNAs generated from non-coding genes, accumulated evidence suggests that several coding genes, such as those encoding PPR, NB-LRR disease resistance proteins, and MYB transcription factors, also generate phased siRNAs^5, 6, 9–16^.

PhasiRNAs are known to be involved in abiotic^17, 18^ and biotic stress responses^12^. In Solanaceae plants, several miRNAs can trigger phasiRNA production from *NB-LRR* loci in response to biotic stress. Moreover, the *tasNB-LRR* regulator cascades triggered by miR482 family members are conserved pathways in many plants, particularly in Solanaceae species^11, 12^. Shivaprasad *et al*. showed that miR482a can trigger the generation of phasiRNAs from an *NBS-LRR* gene and enhance the infection of *Pseudomonas syringae* DC3000^11^. Yang *et al*. showed that overexpressed miR482e in potato increased plant sensitivity to *Verticillium dahliae* infection through NBS-LRR targeting and secondary phasiRNA generation^19^. In a previous study, 90 *PHAS* loci were predicted using the PhaseTank pipeline on the tomato genome; however, no further analysis for these candidates was presented, such as locus information, chromosome distribution, or experimental evidence^20^. To identify *Botrytis cinerea-*responsive phasiRNAs in tomato in this study, we used the PhaseTank pipeline for identification of *PHAS* loci, with two more sRNA datasets (from mock- and *B. cinerea*-infected tomato leaves) compared to our previous study^20^. The candidate genes were also characterized and annotated in terms of overlap with annotated genes, chromosome distribution, and tissue expression. RT-PCR was performed to detect and validate the primary transcripts and to investigate their tissue expression. Two candidates were selected for experimental validation, and the results demonstrated that phasiRNAs predicted by PhaseTank were indeed generated from their respective *PHAS* loci. Finally, phasiRNAs responsive to *B. cinerea* were investigated based on sRNA-seq data, and their targets were characterized using degradome data and gene ontology enrichment analysis.

## Results

### Identification and annotation of *PHAS* loci and phasiRNAs in tomato

For genome-wide detection of phased sRNAs, PhaseTank^21^ was employed along with sRNA libraries and tomato genome sequences. A total of 91 sequences were identified as potential *PHAS* loci in the tomato genome (Tables S1 and S2), with 51 and 40 loci located in annotated genic regions and intergenic regions, respectively. Thirty-one loci were related with disease resistance, including 28R genes; the remaining three were other types of disease resistance genes (Table S2). Among the 40 intergenic *PHAS* loci, 18 passed the protein-coding-score test and were considered as potential coding RNAs according to the Coding Potential Calculator (CPC) (Table S2). ORF prediction of the remaining 22 intergenic *PHAS* loci revealed that all of them were less than 100 amino acids in length. According to a previous report, these genes with unknown functions are lincRNA genes^21^. This phenomenon has also been reported in recent studies on rice^22^.

### Identification of putative phasiRNA triggers

**Figure 1:**
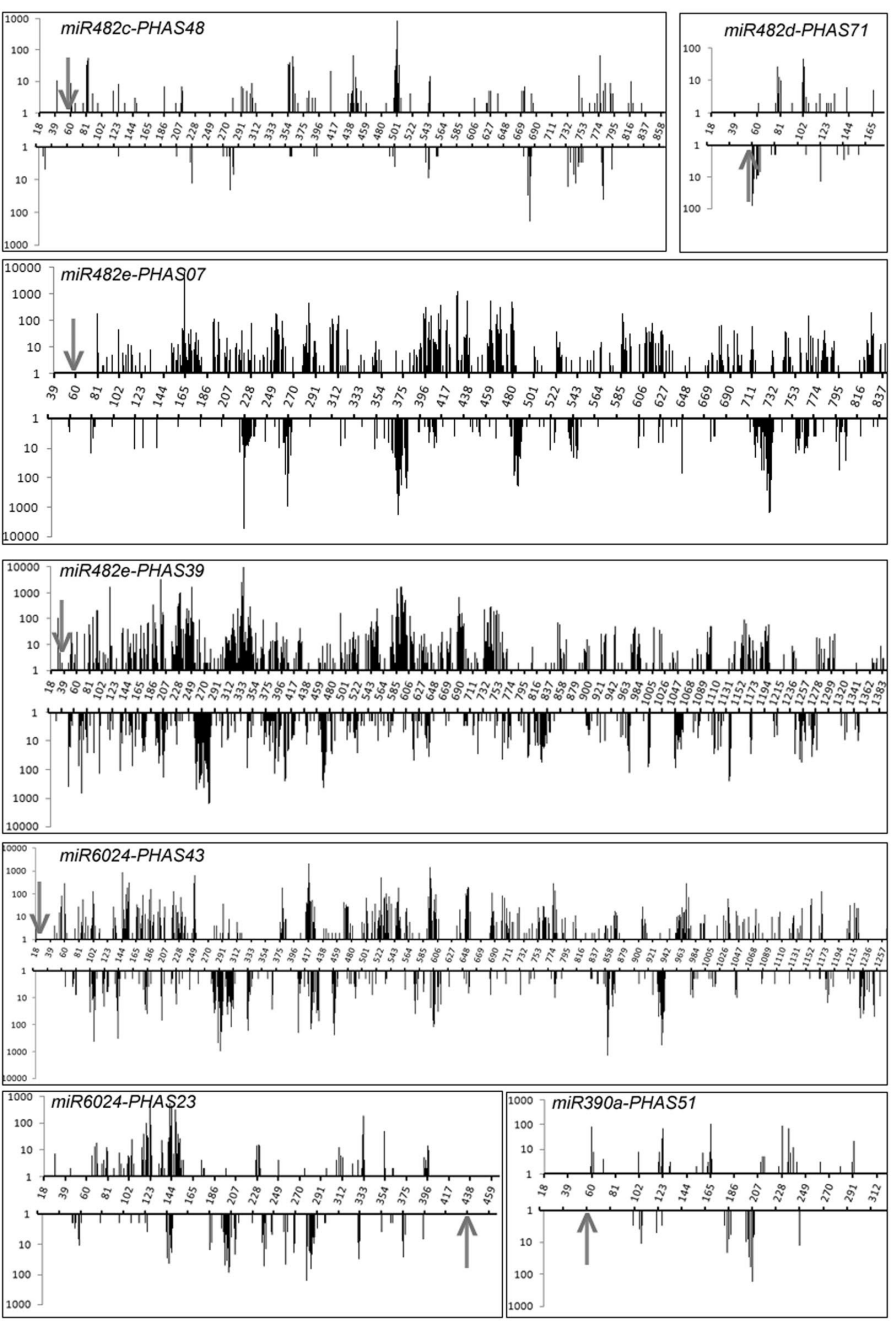
Read abundance distribution of seven *PHAS* loci in the sRNA library. The arrows indicate the miRNA cleavage sites. The X-axis represents a double-stranded *PHAS* locus. Abscissa indicates the phasiRNA position mapped within the *PHAS* locus. Ordinate indicates the read abundance of the mapped sRNAs.

For these 91 *PHAS* loci, miRNA binding site prediction was performed using the psRNAtarget pipeline (Table S3). Degradome datasets produced from tomato leaves, roots, and fruits were applied as the experimental supports for the identification of potential phasiRNA triggers. Seven *PHAS* loci (*PHAS23*, *PHAS43*, *PHAS07*, *PHAS39*, *PHAS71*, *PHAS48*, and *PHAS51*) targeted by five miRNA triggers were subsequently confirmed (Fig. 1).

### Transcript activity analysis of 91 *PHAS* loci

Nearly half of the 91 putative *PHAS* genes were located in intergenic regions (40 loci). Their transcriptional activity was therefore investigated to assess their biogenesis and biological functionusing transcriptome data from seedlings, flowers, roots, and fruits and Tophat^23^ and Cufflinks^24^ analysis. Thirty-five of the 91 *PHAS* loci were transcribed in these tissues; the remaining 56 candidates were not detected (Table S2).

RT-PCR was also used to detect the transcript levels of the 91 *PHAS* loci. Thirty-seven *PHAS* genes were amplified in a mixed tissue sample of tomato roots, stems, and leaves (Table 1 and Figure S1). No amplicons were detected for the remaining 54 *PHAS* candidates. The 37 amplified *PHAS* genes were sequenced for validation and submitted to GenBank (accession numbers KU555888–KU555924) (Table 1). Except for *Sly-PHAS08*, *Sly-PHAS37*, and *Sly-PHAS38*, sequence analysis revealed that all amplicons had >99% alignment, confirming the transcriptional activities of 34 *PHAS* gene loci. Interestingly, only 15 *PHAS* candidates could be detected using both transcriptome data and RT-PCR. Nineteen candidates, which were not detected from the transcriptome data, were amplified in the mixed samples, suggesting tissue-specific transcription in tomato stems. The remaining 57 *PHAS* candidates were not detected, and their transcript levels could not be validated by RT-PCR. The failure to detect these putative *PHAS* genes may be attributable to expression at extremely low levels or in a highly tissue-specific manner. Moreover, this fraction of phasiRNAs may not be amenable to confirmation through these means.

**Table 1:** Transcriptional confirmation and tissue expression of 91 putative *PHAS* genes based on RT-PCR.

PHAS_ID	Accession No.	Root	Stem	Leaf	PHAS_ID	Accession No.	Root	Stem	Leaf
PHAS02	KU555888	√	√	√	PHAS21	KU555907	√	√	√
PHAS03	KU555889	√	√	√	PHAS22	KU555908	√	√	√
PHAS04	KU555890	√	√	√	PHAS23	KU555909	√	√	√
PHAS05	KU555891	No	√	√	PHAS24	KU555910	√	√	√
PHAS06	KU555892	√	√	√	PHAS25	KU555911	√	√	√
PHAS07	KU555893	√	√	√	PHAS26	KU555912	√	√	√
PHAS08	KU555894	No	√	No	PHAS27	KU555913	√	√	√
PHAS09	KU555895	√	√	√	PHAS28	KU555914	√	√	No
PHAS10	KU555896	√	√	√	PHAS29	KU555915	√	√	√
PHAS11	KU555897	√	√	√	PHAS30	KU555916	√	√	√
PHAS12	KU555898	No	√	No	PHAS31	KU555917	√	√	√
PHAS13	KU555899	√	√	√	PHAS32	KU555918	√	√	√
PHAS14	KU555900	√	√	√	PHAS33	KU555919	√	√	√
PHAS15	KU555901	√	√	√	PHAS34	KU555920	√	√	√
PHAS16	KU555902	No	No	√	PHAS35	KU555921	No	√	√
PHAS17	KU555903	No	√	√	PHAS36	KU555922	√	√	√
PHAS18	KU555904	√	√	√	PHAS37	KU555923	√	√	√
PHAS19	KU555905	√	√	√	PHAS38	KU555924	√	No	√
PHAS20	KU555906	√	√	√					

### Experimental validation of two *PHAS* loci and their phasiRNAs

**Figure 2:**
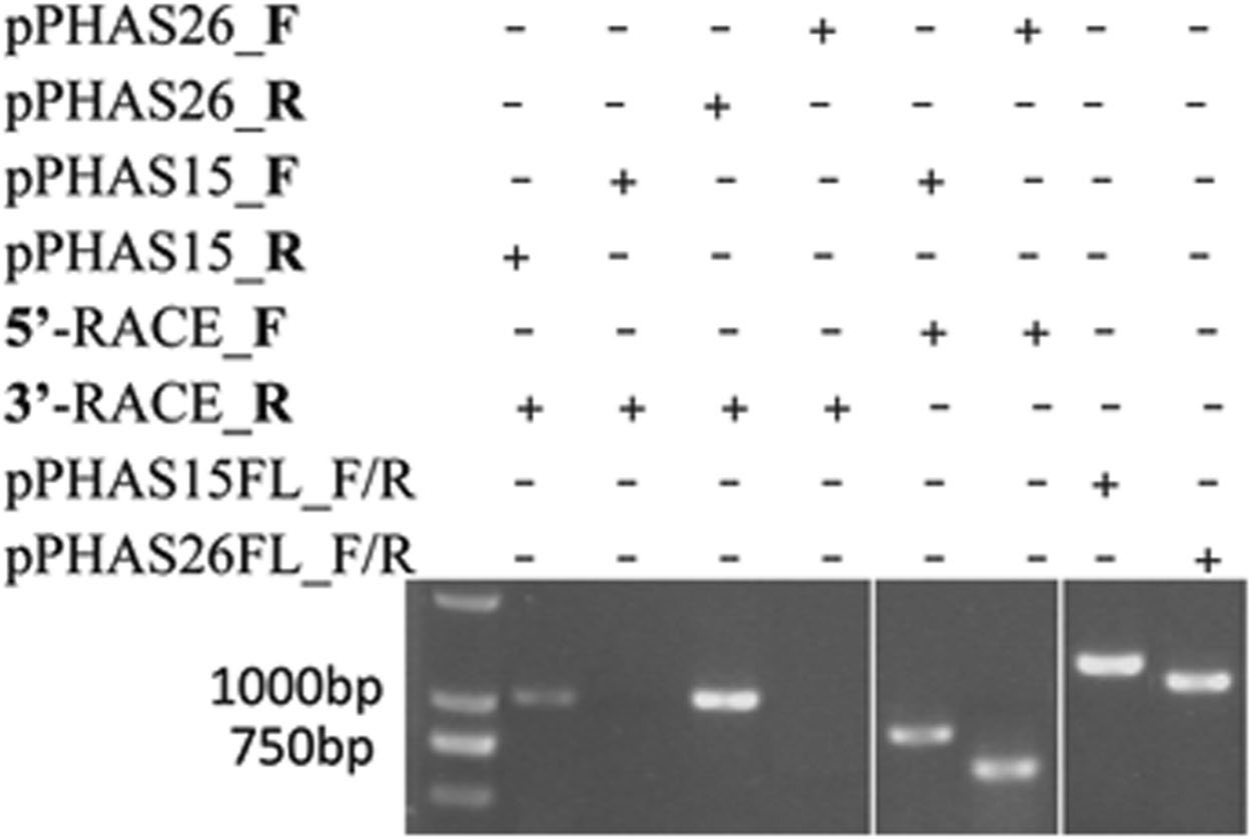
RACE analysis for *PHAS15* and *PHAS26*.

If the analysis above is correct, the transcription of the identified *PHAS* genes should be possible in tomato along with the production of their phasiRNAs. To test this, two *PHAS* loci (*PHAS15* and *PHAS26*) and their phasiRNAs (siR15-2 and siR15-6 from *PHAS15* and siR26-2 and siR26-4 from *PHAS26*) were selected for validation through transient expression in tomato leaves and detection of the phasiRNAs. *PHAS15* is located at the *Solyc06g054600.2* locus, which encodes zinc finger CCCH domain-containing protein 58, while *PHAS26* is intergenic. A RACE kit was used to amplify the respective 5’ and 3’ sequences, and the results revealed that both *PHAS15* and *PHAS26* were transcribed from the negative strand. This means that *PHAS15* was not from the genic region of *Solyc06g054600.2* (Table S2); instead, it was encoded by the antisense strand of *Solyc06g054600.2* (Fig. 2). According to the 5’ and 3’ sequences of *PHAS15* and *PHAS26*, both full-length cDNAs were also amplified from tomato leaves and sequenced (Fig. 2). The sequence analysis showed that *PHAS15* is 1193 bp in length and located on chromosome 6 from 37325436 to 37326628, partially overlapping the antisense strand of *Solyc06g054600.2*. *PHAS26* is 1020 bp in length and located in an intergenic region of chromosome 11 from 18739580 to 18740598 (Figure S2).

**Figure 3:**
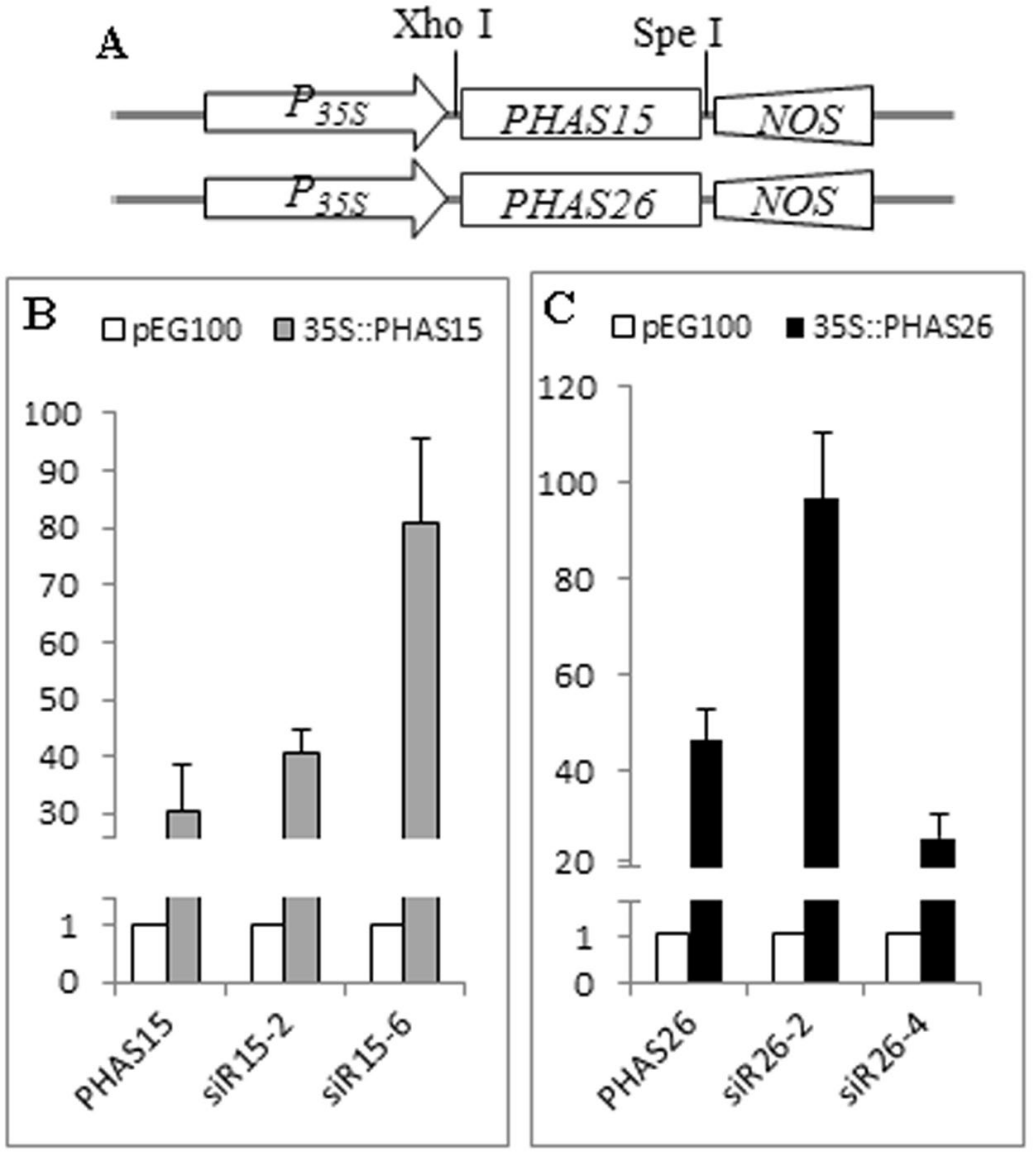
Transient overexpression. (**A**) Construction of the *PHAS* overexpression vectors. (**B**) The expression levels of *PHAS15* and the corresponding phasiRNAs were detected in p35S::PHAS15-transformed leaves and control leaves. (**C**) The expression levels of *PHAS26* and the corresponding phasiRNAs were detected in p35S::PHAS26-transformed leaves and control leaves. Ubi3 was used as the internal control. Error bars indicate SD from three biological repeats; the normalized levels of the control were set arbitrarily to 1.

Next, overexpression vectors of *PHAS15* and *PHAS26* were constructed by fusing *PHAS15* and *PHAS26* with the CaMV35S promoter (Fig. 3A). *Agrobacterium tumefaciens* LBA4404 cells containing either the *PHAS15* or *PHAS26* overexpression vectors were injected into tomato leaves on one side of the midvein. LBA4404 cells with blank pEarleyGate100 (PEG100) vector were also cultured and injected into tomato leaves on one side of the midvein as the control. After 24 h, RT-qPCR analysis was performed. The results showed that *PHAS15*, *PHAS26*, and the four phasiRNAs were transiently overexpressed in the leaves transformed with the *PHAS* fusion vectors, demonstrating that the identified phasiRNAs are indeed generated from the respective *PHAS* loci (Fig. 3B and C). These results confirmed the PHAS identification from the PhaseTank pipeline.

### Identification of *B. cinerea*-responsive phasiRNAs and experimental evidence

As indicated above, 31 of the identified *PHAS* loci mapped to the coding regions of genes encoding disease resistance-related proteins, suggesting that phasiRNA could be responsive to disease infection. To investigate phasiRNAs responsive to *B. cinerea* in tomato, reads from two small RNA libraries of tomato leaves inoculated with *B. cinerea* (infected and control) were analyzed. A total of 245 phasiRNAs with relatively high expression were subjected to further analysis. Among these 245 phasiRNAs, 50 from 27 loci hand increased abundance and five phasiRNAs from five loci had decreased abundance in *B. cinerea*-infected leaves (Table S4).

**Figure 4:**
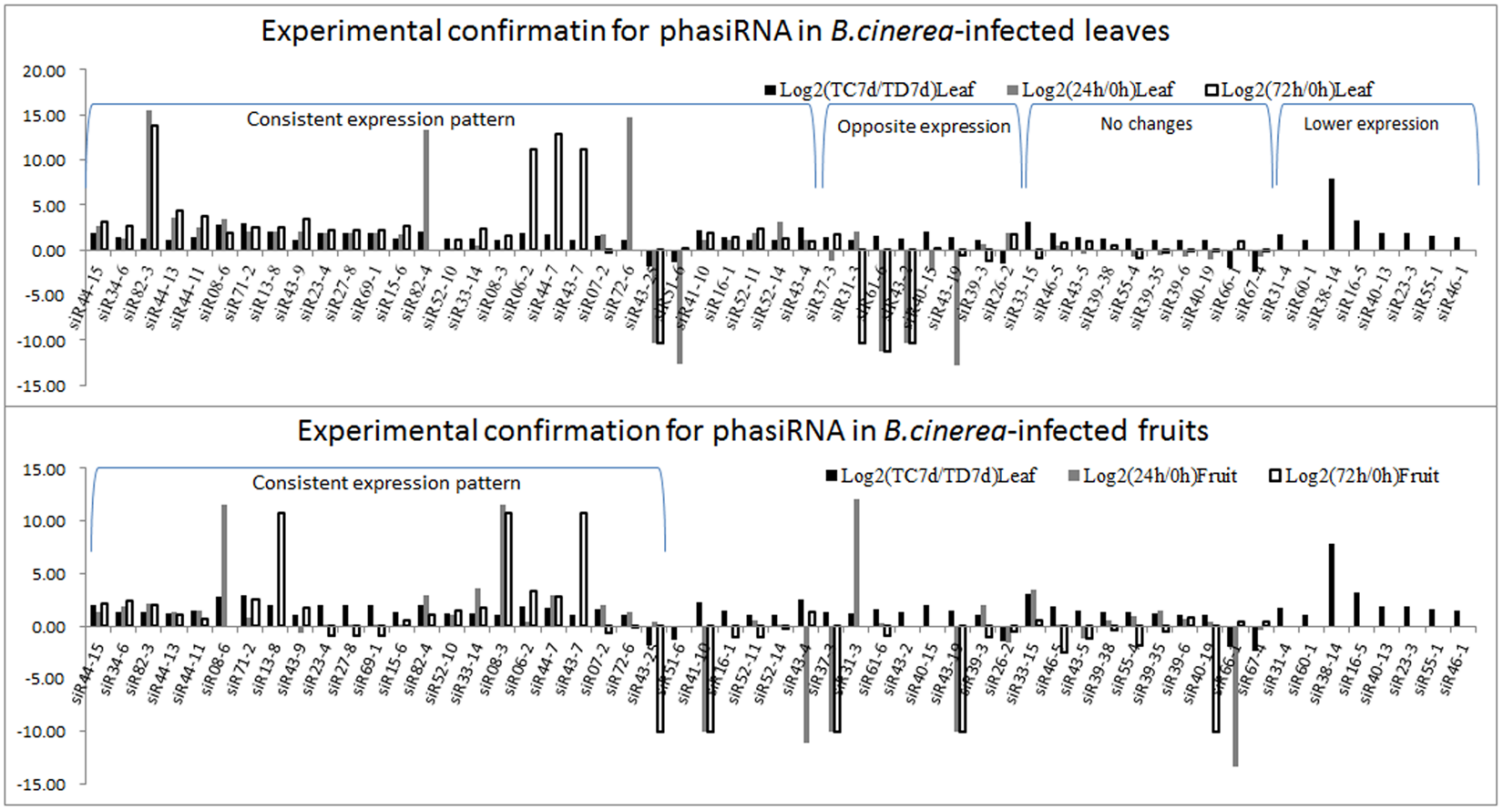
Experimental confirmation for *B.cinerea*-responsive phasiRNAs in tomato leaves (**A**) and fruits (**B**).

To validate these 55 *B. cinerea*-responsive phasiRNAs, sRNA-seq data from *B. cinerea*-inoculated tomato leaves at three time points (0 h, 24 h, and 72 h) were used^25^. Twenty-nine phasiRNAs were confirmed, including 27 phasiRNAs with increased abundance and two with decreased abundance, and the expression patterns were consistent with our sRNA-seq data. Another eight phasiRNAs also had altered expression in *B. cinerea*-infected leaves, but an opposite change in expression patterns was observed. Among the remaining 18 phasiRNAs, no expression changes were found for ten phasiRNAs, and expression analysis could not be performed for the remaining eight phasiRNAs (siR31-4, siR60-1, siR38-14, siR16-5, siR40-13, siR23-3, siR55-1, and siR46-1) due to their very low read counts (<10 TP10M) (Fig. 4A and Table S5). sRNA-seq data from *B. cinerea*-infected tomato fruits at the same time points were also used. Among the 29 confirmed phasiRNAs, 23 (22 with increased abundance and one with decreased abundance) were further validated with consistent expression patterns in fruit (Fig. 4B and Table S5) and will thus be analyzed in subsequent works.

From the analysis of phasiRNAs responsive to *B. cinerea* infection, five phasiRNAs triggered by four conserved miRNAs (miR6024, miR482d, miR482e, and miR390a) were also found. SiR51-6 from miR390a-PHAS51, which was highly homologous to TAS3, had decreased abundance in *B. cinerea*-infected leaves, but no expression signal was detected in tomato fruits. Otherwise, the remaining four phasiRNAs triggered by conserved miRNAs (siR07-2 triggered by miR482e, siR71-2 triggered by miR482d, and both siR23-4 and siR43-7 triggered by miR6024) had increased abundance in both *B. cinerea*-infected leaves and *B. cinerea*-infected fruits (Fig. 4).

### Target prediction of phasiRNAs and degradome evidence

**Figure 5:**
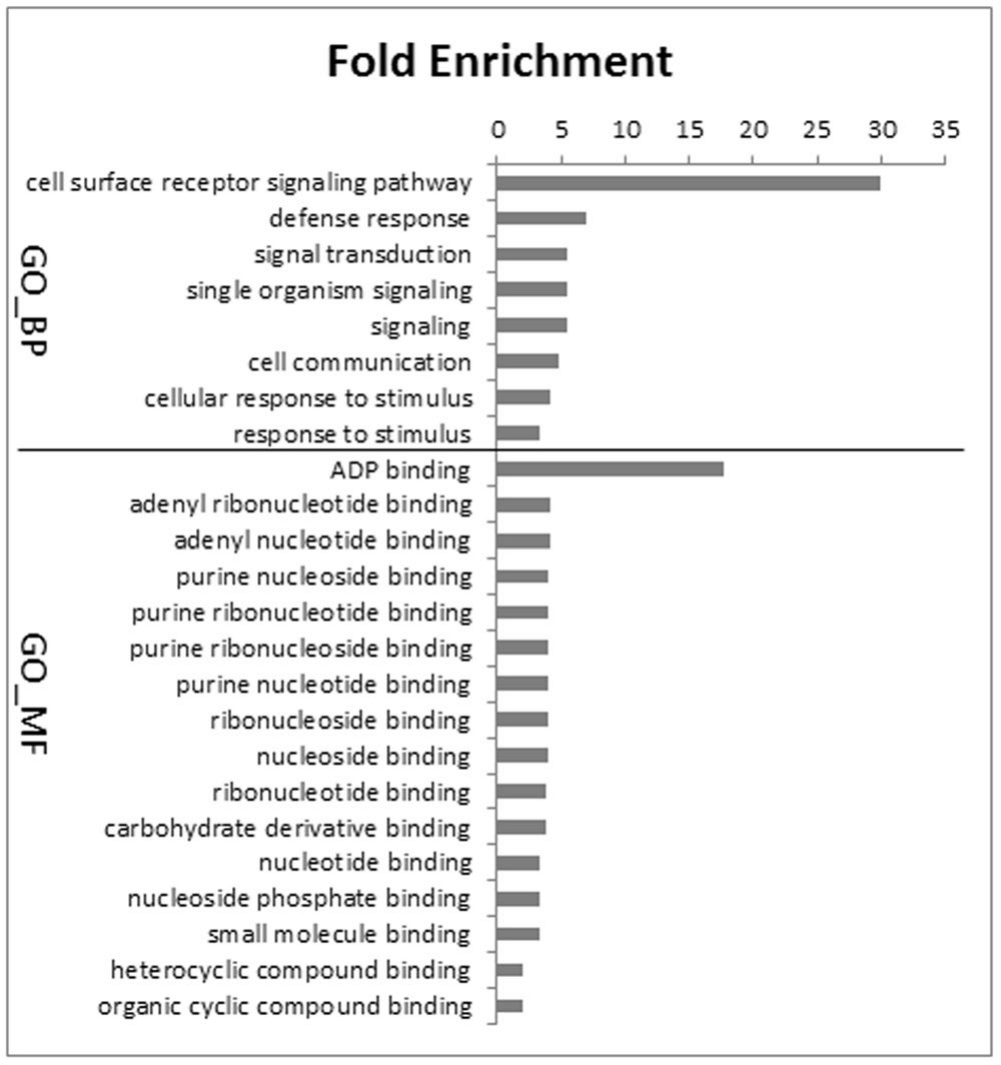
GO functional enrichment analysis of the target genes of *B. cinerea*-responsive phasiRNAs.

To identify the downstream regulatory pathway of the phasiRNAs, their targets were searched by identifying complementary regions from all tomato transcripts. Using psRNAtarget with default parameters, 164 putative targets were found (Table S6) and further validated with degradome data. The analysis showed that the cleavage start positions of 94 targets overlapped with degradome fragments, and these findings were considered as experimental validation (Table S6). GO enrichment analysis indicated that all of the targets are binding proteins related to defense responses or signaling regulation (Fig. 5). Among them, 11 targets were also the site of a *PHAS* gene locus.

## Discussion

PhasiRNAs have been shown to have important roles in developmental regulation and stress responses. Genome-wide identification of phasiRNAs in a given organism is an important tool that can be used to investigate gene regulation involving small RNAs and is as important as mining genes that code for proteins. The establishment of a comprehensive list of phasiRNAs from any organism will be instrumental not only for gene regulation studies but also for genome organization, phylogenetic comparison, comparative development, and other evolutionary analyses. In this study, the PhaseTank pipeline identified 912 phasiRNAs from 91 *PHAS* loci in the tomato genome. However, only seven *PHAS* loci (*PHAS23*, *PHAS43*, *PHAS07*, *PHAS39*, *PHAS71*, *PHAS48*, and *PHAS51*) were identified as the targets of five miRNA triggers and subsequently confirmed based on degradome data. Trigger miRNAs were not found for the remaining 84 *PHAS* loci, and possible explanations are as follows. 1) The tomato miRNAs deposited into the miRBase database is not complete, and a large number of miRNAs may still be found. Thus far, 713 and 427 miRNAs have been reported in rice and *Arabidopsis*, respectively. In potato, which is in the same family as tomato, 343 miRNAs have been reported. In contrast, only 110 miRNAs have been deposited into miRBase (version 21.0) for tomato^26^. Therefore, incomplete information about tomato miRNAs may hinder the identification of trigger miRNAs. 2) Detection failure may also reflect the possibility that many of these *PHAS* loci are triggered by siRNAs rather than miRNAs.

As first described in Arabidopsis^5^, phasiRNAs are generated from both protein-coding regions^5, 6, 15, 16^ and long none-coding regions^27–29^ in many plant genomes. In this study, 51 coding and 40 non-coding regions were identified as the sites of *PHAS* loci in tomato. Among the coding regions identified as *PHAS* loci, NB-LRR was predominantly represented. NB-LRR regulation by secondary siRNAs has been reported to exist widely in Solanaceae species^6, 11, 12^. Most recently, an examination of numerous NB-LRRs in a wide variety of plant species demonstrated significant levels of secondary siRNAs in Norway spruce (*Picea abies*, a gymnosperm), *Amborella* (a basal angiosperm), cotton (*Gossypium hirsutum*), poplar (*Populus spp*.), grapevine (*Vitis vinifera*), apple, and peach, indicating broad conservation and an ancient origin for the role of phasiRNAs in the regulation of NB-LRRs^13^.

PhasiRNAs have also recently been shown to be involved in biotic stress responses^12^. In this study, *B. cinerea*-responsive phasiRNAs, including 27 with increased abundance and two with decreased abundance, were identified and validated in *B. cinerea*-infected tomato leaves. Moreover, 22 of the 27 phasiRNAs with increased abundance and one of the two phasiRNAs with decreased abundance also had the same expression patterns in *B. cinerea*-infected tomato fruits. Three miRNA-PHAS regulation cascades (miR482d-PHAS, miR482e-tasLRR, and miR6024-tasLRR) were also identified and confirmed in response to *B. cinerea* infection. The miR482 family is well-known as a trigger miRNA involved in phasiRNA biogenesis^11, 19, 30^. In addition, miR6024 may also trigger the production of phasiRNAs at NB-LRR loci in Solanaceae plants^12^. With the focus on responsiveness to *B. cinerea* in mind, many phasiRNAs were predicted to target *R* genes such as *NBS-LRR* or *RLP* genes, which are important for pathogen resistance in higher plants. The *NBS-LRR* gene family is a huge gene family with dozens to hundreds of members per plant genome^31, 32^. While maintaining a large number of *NBS-LRR* genes may be beneficial for plant defense against different pathogens, maintaining their expression under a pathogen-absent environment may incur a fitness cost^33^. Therefore, phasiRNA-targeted *R* genes may play important roles in biotic stress responses. Putative targets were also found for these *B. cinerea*-responsive phasiRNAs, including a NAC transcription factor; MADS-box transcription factor; zinc finger CCCH domain-containing protein; and a series of plant signal transduction factors, such as an auxin response factor, serine/threonine protein kinase, and F-box protein. Because the identified phasiRNAs clearly responded to *B. cinerea* infection in tomato, their validity warrants further investigation, and confirming their differential expression will provide clues about their biological functions.

**Figure 6:**
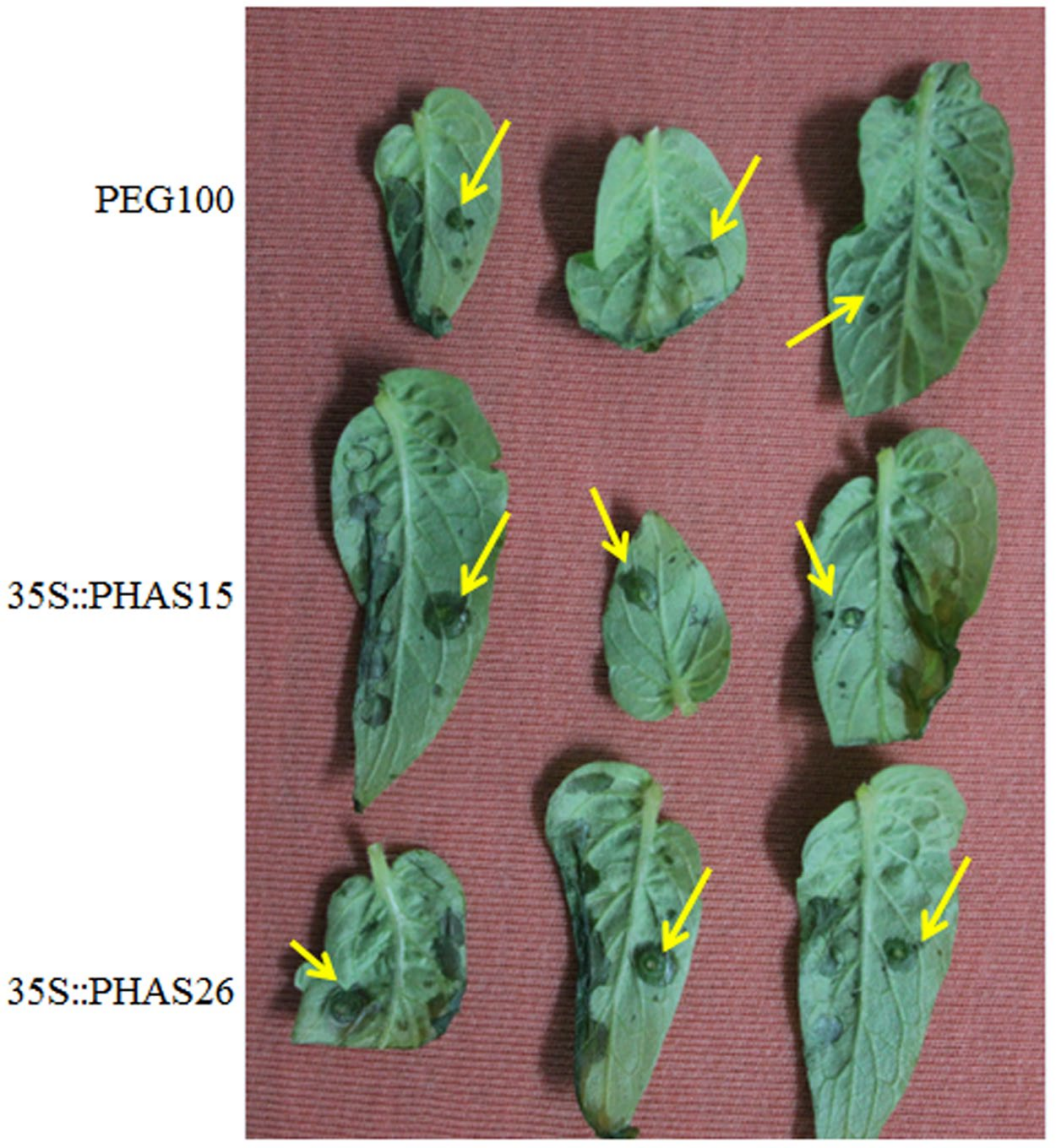
Biological impact of *PHAS15* and *PHAS26*. *Agrobacterium tumefaciens* LBA4404 cells containing the overexpression vectors were injected into tomato leaves on one side of the midvein. LBA4404 cells with blank pEG100 vector were also cultured and injected into tomato leaves on one side of the midvein as the control. After 24 h, the opposite sides of the injected leaves were used for inoculation with *B. cinerea* agar discs (4 mm in diameter) for 48 h. The yellow arrows indicate the inoculation sites of the *B. cinerea* agar discs.

Finally, to gain insight into the biological effect of the identified *B. cinerea*-responsive phasiRNAs, *PHAS*-overexpressing leaves confirmed by qRT-PCR (Fig. 3B and C) were also inoculated with *B. cinerea* agar discs. After 48 h, the results showed that the phasiRNAs led to larger pathogenic spots in the leaves overexpressing *PHAS15* or *PHAS26* compared to control leaves (Fig. 6). Although the transient expression results need to be validated by other experiments, the findings suggest that these *B. cinerea*-responsive phasiRNAs might be involved in the regulation of *B. cinerea* infection in tomato.

## Materials and Methods

### Data retrieval

Three small RNA libraries (GSM803579, GSM803580, and GSM803581) produced from tomato leaves, flowers, and fruit of *S. lycopersicum* were retrieved from the NCBI Gene Expression Omnibus database under the accession number GSE28755 from Chávez Montes *et al*.^34^. Their study examined the conservation and divergence of microRNAs from 99 different tissues from three algal species and 31 representative vascular plant species. Two additional small RNAs libraries (SRR1482408 and SRR1463412) came from seven day post-inoculation leaves of *B. cinerea*-inoculated and mock-inoculated plants; these data were produced by Jin *et al*.^35^ to identify *B. cinerea*-responsive miRNAs in tomato and retrieved from the NCBI Sequence Read Archive (SRA) database.

Three degradome datasets (GSM553688, GSM553689, and GSM5536903) produced by Lopez-Gomollon *et al*.^36^ from tomato leaves, roots, and fruits were retrieved from the NCBI Gene Expression Omnibus database under the accession number GSE22236. In addition, four paired-end mRNA sequencing libraries (SRX1227045, SRX1227046, ERX1604048, and SRX1227066) produced from the seedlings, flowers, roots, and fruits were downloaded from the NCBI SRA database.

### Identification of phasiRNA candidate genes

To detect phased sRNAs in tomato, PhaseTank^21^ was used with default parameters on five sRNA libraries (GSM803579, GSM803580, GSM803581, SRR1482408, and SRR1463412) downloaded from the NCBI SRA database and tomato genome sequences (Version SL2.50) downloaded from the ftp site (ftp://ftp.solgenomics.net/genomes/Solanum_lycopersicum/annotation/).

### Transcription activity analysis of 91 *PHAS* loci

Four paired-end mRNA sequencing libraries (SRX1227045, SRX1227046, ERX1604048, and SRX1227066) produced from tomato leaves, flowers, roots, and fruits were downloaded from the NCBI SRA database. These RNA-seq data were used to analyze the transcription of 91 *PHAS* loci through Tophat^23^ and Cufflinks^24^. Low quality reads were removed with fastq_quality_filter using the options –q 20 and –p 80 (http://hannonlab.cshl.edu/fastx_toolkit/). Filtered reads from each sample were aligned to the tomato reference genome (SL2.50) using Tophat^23^. The transcriptome of each sample was assembled separately using Cufflinks, and Cuffmerge was used to merge the assemblies produced by Cufflinks for all samples^24^. If the genomic coordinate of a given *PHAS* locus overlapped with a merged assembly locus, the transcription of the *PHAS* locus was considered confirmed.

### Protein-coding-score test and open reading frame assay

The Coding Potential Calculator^37^, which detects the quality, completeness, and sequence similarity of an ORF to proteins in current protein databases, was used to predict putative protein-encoding transcripts of PHAS loci with default parameters. Only loci that did not pass the protein-coding-score test were further analyzed for ORF prediction. A Perl script was written to investigate the intergenic PHAS loci that encode ORFs of 100 or fewer amino acids through six-frame translation.

### Target prediction and validation of miRNAs and phasiRNAs

In this report, the targets of miRNAs and phasiRNAs were initially predicted using the online tool psRNATarget^38^. Next, the cleavage start position was validated by mapping the target sequences with three degradome datasets (GSM553688, GSM553689, and GSM5536903) produced from tomato leaves, roots, and fruits. If any RNA degradome fragment was complementary to the target PHAS of a miRNA and the 5’-end of the degradome fragment overlapped the PHAS cleavage start position, the start position of miRNA-guided *PHAS* cleavage was considered validated^6, 39^. Validation of the phasiRNA cleavage site was also performed using the RNA degradome. If any RNA degradome fragment was complementary to the target transcript of a phasiRNA and the 5’-end of the degradome fragment overlapped with the 8–12 positions of the phasiRNA, the cleavage site was considered confirmed.

### GO enrichment analyses

GO enrichment analysis was applied to target transcripts using an online tool of the PANTHER Classification System^40^. GO terms with P < 0.05 were considered significantly enriched.

### RNA extraction and RT-PCR analysis

Total RNAs were extracted using TRNzol-A^+^ Reagent (TIANGEN, Beijing, China), followed by RNase-free DNase treatment (Takara, Dalian, China). The concentrations were quantified using a NanoDrop ND-1000 spectrophotometer.

Reverse transcription was performed using the PrimeScript RT^®^ reagent kit (Perfect Real Time) (TaKaRa, Dalian, China). All *PHAS* and target genes were subjected to RT-PCR validation and quantitation using specific primers (Table S7), which were designed using Primer Premier 5. The reaction conditions were as follows: 95 °C for 3 min; 30 cycles of 95 °C for 30 s, 49–62 °C for 30 s, and 72 °C for 40 s; and final elongation at 72 °C for 10 min.

Reverse transcription was also performed using the One Step PrimeScript miRNA cDNA Synthesis Kit (TaKaRa, Dalian, China) according to the manufacturer’s protocol for phasiRNA quantitation. All phasiRNAs were subjected to qRT-PCR validation using specific primers (Table S1), which were designed using Primer Premier 5. The reaction conditions were as follows: 94 °C for 4 min; 30 cycles of 94 °C for 30 s, 60 °C for 40 s, and 72 °C for 40 s; and final elongation at 72 °C for 5 min.

### Construction of the expression vectors

The full-length cDNAs of *PHAS* genes were amplified and cloned into pGEM-T. After sequencing validation and digestion, the *PHAS* sequence was introduced into the pEG100 vector using the Xho I and Spe I restriction sites. The resulting construct contained the *PHAS* gene driven by the cauliflower mosaic virus 35S (CaMV35S) promoter and terminated by nos (Fig. 3A). The construct was introduced into *A. tumefaciens* LBA4404 for transient expression in tomato leaves.

### Transient overexpression

*A. tumefaciens* LBA4404 cells transformed with recombinant pEG100-PHAS plasmid was cultured at 28 °C in LB medium supplemented with kanamycin (50 μg/ml) and rifampicin (20 μg/ml). When turbidity at 600 nm reached 1.0, the cells were collected by centrifugation at 5,000 rpm for 5 min and washed twice in resuspension buffer containing 10 mmol MES (pH 5.6), 10 mmol MgCl2, and 200 μmol acetosyringone. The cell pellets were resuspended in resuspension buffer at an OD600 of 0.5 then injected into the tomato leaves. LBA4404 cells transformed with the empty pEG100 vector were also cultured and used in the same manner to serve as the control.

For the transient overexpression of *PHAS*, tomatoes (*Solanum lycopersicum* cv. micro-Tom) were used as the host plant. They were grown in a greenhouse with a 16-h day/8-h night cycle at 22–28 °C. At the age of 6 weeks, LBA4404 cells containing the overexpression vectors were injected into tomato leaves on one side of the midvein. LBA4404 cells with blank pEG100 vector were also cultured and injected into tomato leaves on one side of the midvein as the control. After 24 h, the other sides of the injected leaves were harvested in three biological replicates. The samples were frozen in liquid nitrogen and stored at −70 °C for the transcript expression analysis.

### Quantitative real-time PCR analysis

Expression profiles of the *PHAS* genes and phasiRNAs were assayed by quantitative reverse transcription PCR (qRT-PCR). Total RNA was treated with RNase-free DNase I (TaKaRa, Dalian, China) to remove genomic DNA. For the PHAS genes, first-strand cDNA synthesis was performed using SuperScript II Reverse Transcriptase (Invitrogen, USA). For the phasiRNAs, the reverse transcription reaction was performed using the One Step PrimeScript miRNA cDNA Synthesis Kit (TaKaRa, Dalian, China) according to the manufacturer’s protocol^41^. All of the oligos used in this study are listed in Supplemental Table S7.

SYBR Green PCR was performed as per the manufacturer’s instructions (Takara, Japan). Briefly, 2 μl of cDNA template was added to 12.5 μl of 2 × SYBR Green PCR master mix (Takara), 1 μM concentration of each primer, and ddH2O to a final volume of 25 μl. The reactions were amplified for 10 s at 95 °C, followed by 40 cycles of 95 °C for 10 s and 60 °C for 30 s. All reactions were performed in triplicate, and the controls (no template and no RT) were included for each gene. The threshold cycle (CT) values were automatically determined by the instrument, and the fold change was calculated using the following equation: 2^−ΔΔCt^, where ΔΔC_T_ = (C_T,target_ − C_T,inner_)_Infection_ − (C_T,target_ − C_T,inner_)_Mock_^42^.

### Identification of *B. cinerea*-responsive phasiRNAs

The deep sequencing data for small RNAs in 7 days post-inoculation leaves of *B. cinerea*-infected (TD7d) and control (TC7d) plants were used (accession numbers SRR1482408 and SRR1463412). The frequency of phasiRNAs was normalized as ‘reads per million’ (RPM = mapped reads/total reads × 1,000,000). The fold_change between the TD7d and TC7d libraries was calculated using the following equation: fold_change = log_2_ (RPM_TD7d_/RPM_TC7d_). PhasiRNAs with fold-changes >1 or <−1 and *p*-values ≤ 0.001 were considered to have increased abundance or decreased abundance in response to *B. cinerea* stress, respectively. The *p*-value was calculated according to previously established methods^43^.

## Electronic supplementary material

Supplementary files
