# Supplementary material for: Genome-wide identification and characterization of phased small interfering RNA genes in response to Botrytis cinerea infection in Solanum lycopersicum
Source: Sci Rep. 2017 Jun 8;7:3019. doi: 10.1038/s41598-017-02233-x (PMC5465084; doi:10.1038/s41598-017-02233-x)
Supplement: Supplementary file 1 — Supplementary files [file 41598_2017_2233_MOESM1_ESM.pdf]

Genome-wide identification and characterization of phased small interfering RNA genes in response to *Botrytis cinerea* infection in *Solanum lycopersicum*

Fangli Wu, Yue Chen, Xing Tian, Xiaole Zhu, Weibo Jin*

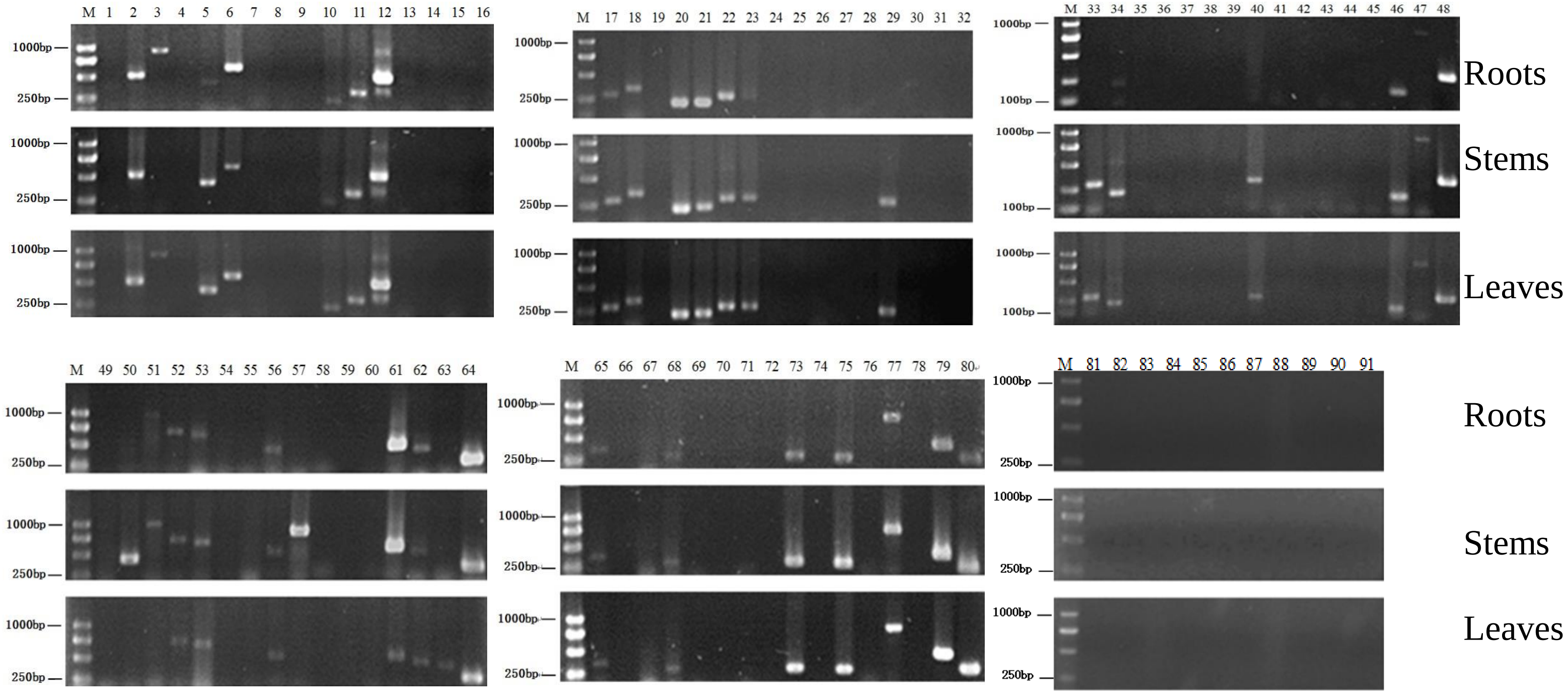

Figure S1. Transcriptional activities of the 91 *PHAS* loci detected by RT-PCR

Genome-wide identification and characterization of phased small interfering RNA genes in response to *Botrytis cinerea* infection in *Solanum lycopersicum*

Fangli Wu, Yue Chen, Xing Tian, Xiaole Zhu, Weibo Jin[1,*]

Figure S2. Sequence analysis of the two full-length cDNAs of *PHAS15* and *PHAS26*

```
>SL2.50ch06         Length = 49751636

 Score = 2325 bits (1173), Expect = 0.0
 Identities = 1189/1193 (99%)
 Strand = Plus / Minus


PHAS15: 1       tatgggtttgcgtaggcaggtgctgaagcagatattcgtgtatgaccataaactggacct 60
                ||||||||||||||||||||||||||||||||||||||||||||||||||||||||||||
Sbjct: 37326628 tatgggtttgcgtaggcaggtgctgaagcagatattcgtgtatgaccataaactggacct 37326569

PHAS15: 61      actccagctgcagagagagaagtttggcatggcactggacctgctccagctgcagattga 120
                ||||||||||||||||||||||||||||||||||||||||||||||||||||||||||||
Sbjct: 37326568 actccagctgcagagagagaagtttggcatggcactggacctgctccagctgcagattga 37326509

PHAS15: 121     gtagtttggtatggcactggacaagtagaaccctgcaccagataatttaatacttgccag 180
                ||||||||||||||||||||||||||||||||||||||||||||||||||||||||||||
Sbjct: 37326508 gtagtttggtatggcactggacaagtagaaccctgcaccagataatttaatacttgccag 37326449

PHAS15: 181     ataaataatgcatttcaggaaagatctatatcaacagctgccacaaacacttataatatc 240
                ||||||||||||||||||||||||||||||||||||||||||||||||||||||||||||
Sbjct: 37326448 ataaataatgcatttcaggaaagatctatatcaacagctgccacaaacacttataatatc 37326389

PHAS15: 241     cagataaggagtccaatccgacacgggaaccactctagggggaaaaagcataggaccata 300
                ||||||||||||||||||||||||||||||||||||||||||||||||||||||||||||
Sbjct: 37326388 cagataaggagtccaatccgacacgggaaccactctagggggaaaaagcataggaccata 37326329

PHAS15: 301     cgtgtcaggaatatagaaacctggaagcagagcaggccctataactggccagtaaccatt 360
                |||||||||||||||||||||||||||||||||||||||||||||||||||||||||||
Sbjct: 37326328 tgtgtcaggaatatagaaacctggaagcagagcaggccctataactggccagtaaccatt 37326269

PHAS15: 361     ccatattgttcagctgaatcaacaggaggaagaggttgcagttctggataagtggctggc 420
                ||||||||||||||||||||||||||||||||||||||||||||||||||||||||||||
Sbjct: 37326268 ccatattgttcagctgaatcaacaggaggaagaggttgcagttctggataagtggctggc 37326209

PHAS15: 421     ggtggaacagtcgctggcaaaggaaaaggtccagctgctaatgctggaacttgcacacca 480
                |||||||||| |||||||||||||||||||||||||||||||||||||||||||||||||
Sbjct: 37326208 ggtggaacagccgctggcaaaggaaaaggtccagctgctaatgctggaacttgcacacca 37326149

PHAS15: 481     gctggctgtggatggttaaatttacaggtgataccaaatttacagtgccctgttttcata 540
                ||||||||||||||||||||||||||||||||||||||||||||||||||||||||||||
Sbjct: 37326148 gctggctgtggatggttaaatttacaggtgataccaaatttacagtgccctgttttcata 37326089

PHAS15: 541     tagtgtgcacattccttttcattctgtgaaacaaactcaatgaaccagatcagaaggata 600
                ||||||||||||||||||||||||||||||||||||||||||||||||||||||| ||||
Sbjct: 37326088 tagtgtgcacattccttttcattctgtgaaacaaactcaatgaaccagatcagaaagata 37326029

PHAS15: 601     atttaatggtcaaacatttcaaaggagacgagcaaaagaattgaaaacacaaatcaatgg 660
                ||||||||||||||||||||||||||||||||||||||||||||||||||||||||||||
Sbjct: 37326028 atttaatggtcaaacatttcaaaggagacgagcaaaagaattgaaaacacaaatcaatgg 37325969
```

```
PHAS15: 661     agatagcgatacaaggagacagaaggaaaacataatgaacatgaaaagaagacagagggt 720
                ||||||||||||||||||||||||||||||||||||||||||||||||||||||||||||
Sbjct: 37325968 agatagcgatacaaggagacagaaggaaaacataatgaacatgaaaagaagacagagggt 37325909


PHAS15: 721     cgcagcgggtaaccataaatgttatgtatcaccaggcttggtgattcctctccctgtctt 780
                ||||||||||||||||||||||||||||||||||||||||||||||||||||||||||||
Sbjct: 37325908 cgcagcgggtaaccataaatgttatgtatcaccaggcttggtgattcctctccctgtctt 37325849


PHAS15: 781     ggatggtgatatttgcatgaagcaccaaacttgcacatccccgtcctcatatagtactgt 840
                ||||||||||||||||||||||||||||||||||||||||||||||||||||||||||||
Sbjct: 37325848 ggatggtgatatttgcatgaagcaccaaacttgcacatccccgtcctcatatagtactgt 37325789


PHAS15: 841     aaatcataaccaggaacaactagcattataacattacagtcttatacatgaaagttttgc 900
                ||||||||||||||||||||||||||||||||||||||||||||||||||||||||||||
Sbjct: 37325788 aaatcataaccaggaacaactagcattataacattacagtcttatacatgaaagttttgc 37325729

PHAS15: 901     gagaatttcaaatgcatgacatatagccatataacttgtattcaacagtcacttgggtta 960
                |||||||||||||||||||||||||||||||||||||||||||||||||||||||||||
Sbjct: 37325728 aagaatttcaaatgcatgacatatagccatataacttgtattcaacagtcacttgggtta 37325669


PHAS15: 961     attagcagcaagtccaatattagaaaacctcacaatgtaaatcacagtttggaatagaag 1020
                ||||||||||||||||||||||||||||||||||||||||||||||||||||||||||||
Sbjct: 37325668 attagcagcaagtccaatattagaaaacctcacaatgtaaatcacagtttggaatagaag 37325609


PHAS15: 1021    acctgggctaccgtctacctgaatgtcattattacatgaagtatggggattgcaattttg 1080
                ||||||||||||||||||||||||||||||||||||||||||||||||||||||||||||
Sbjct: 37325608 acctgggctaccgtctacctgaatgtcattattacatgaagtatggggattgcaattttg 37325549


PHAS15: 1081    gatctttgtgtaaatactcttatcatcagtgctatgggtctcctgacctgtaagcaactt 1140
                ||||||||||||||||||||||||||||||||||||||||||||||||||||||||||||
Sbjct: 37325548 gatctttgtgtaaatactcttatcatcagtgctatgggtctcctgacctgtaagcaactt 37325489


PHAS15: 1141    gccatgtaatccttagattgaaattcttcactgcaatttaatgttttatcttt 1193
                |||||||||||||||||||||||||||||||||||||||||||||||||||||
Sbjct: 37325488 gccatgtaatccttagattgaaattcttcactgcaatttaatgttttatcttt 37325436
```

```
>SL2.50ch11
        Length = 56302525

 Score = 1586 bits (800), Expect = 0.0
 Identities = 966/1020 (94%), Gaps = 1/1020 (0%)
 Strand = Plus / Minus


PHAS26: 1       ttagcatgggataacattataggatttcggtcctattacgttggccttcgggatcggagt 60
                |||||||||||||||||||||| |||| ||||||||||| ||||||||||||||||||||
Sbjct: 18740598 ttagcatgggataacattatagaatttgggtcctattacattggccttcgggatcggagt 18740539


PHAS26: 61      aatgattaacagggacagtcgggggcattcgtatttcatagtcagaggtgaaattcttgg 120
                |||||||||||||||||||||||||||||||||||||||| ||| |||||||||||||||
Sbjct: 18740538 aatgattaacagggacagtcgggggcattcgtatttcatattcataggtgaaattcttgg 18740479


PHAS26: 121     atttatgaaagacgaaaaactgcgaacgcatttgccaaggatgttttcattaatcaagaa 180
                ||||||||||||||||||||||||||||||||||||||||||||||||||||||||||||
Sbjct: 18740478 atttatgaaagacgaaaaactgcgaacgcatttgccaaggatgttttcattaatcaagaa 18740419


PHAS26: 181     cgaaagttgggggctcaaagacgatcagatactgtcccagtctcaaccataaacgatgcc 240
                ||||||||||||||||||||||||||||||||||||||||||||||||||||||||||||
Sbjct: 18740418 cgaaagttgggggctcaaagacgatcagatactgtcccagtctcaaccataaacgatgcc 18740359


PHAS26: 241     gaccagggatcggcggatgttgcttttaggactccgccggcaccttatgagaaatcaaag 300
                 |||||||||| |  |||||||||||||||||||||||||||||||||||||||||||||
Sbjct: 18740358 aaccagggatctgatgatgttgcttttaggactccgccggcaccttatgagaaatcaaag 18740299
```

```
PHAS26: 301     tttttgggttccggggggagtatggtcgcaaggctgaaacttaaaggaattgacggaagg 360
                |||||||||||||||||||||||||||| ||||| |||||||||||||||||||||||||
Sbjct: 18740298 tttttgggttccggggggagtatggtcgtaaggccgaaacttaaaggaattgacggaagg 18740239

PHAS26: 361     gcaccaccaggagtggagcctgcggcttaatttgactcaacacggggaaacttaccaggt 420
                ||||||||| ||||||||||| | ||||||||||||||||||||||||||||||||||||
Sbjct: 18740238 gcaccaccaagagtggagcctactgcttaatttgactcaacacggggaaacttaccaggt 18740179

PHAS26: 421     ccagacatagtaaggattgacagactgagagctctttcttgattctatgggtggtggtgc 480
                | |||||||||||||||||||||||| |||||||||||||||||||| || | |||||||
Sbjct: 18740178 ctagacatagtaaggattgacagactaagagctctttcttgattctacgg-tagtggtgc 18740120

PHAS26: 481     atggccgttcttagttggtggagcgatttgtctggttaattccgttaacgaacgagacct 540
                |||| |||||||||||||||||||||||||||||||||||||  ||||||||||||||||
Sbjct: 18740119 atggtcgttcttagttggtggagcgatttgtctggttaattcttttaacgaacgagacct 18740060

PHAS26: 541     cagcctgctaactagctatgcggaggtatcccttcgcggccagcttcttagagggactac 600
                |||||| |||||||||||||||||||||||||||||||||||||||||||||||||||||
Sbjct: 18740059 cagcctactaactagctatgcggaggtatcccttcgcggccagcttcttagagggactac 18740000

PHAS26: 601     ggccttttaggccgcggaagtttgaggcaataacaggtctgtgatgcccttagatgttct 660
                |||||||||||||| | |||||||||||||||||||||||| ||||||||||||| || |
Sbjct: 18739999 ggccttttaggccgtgcaagtttgaggcaataacaggtctgcgatgcccttagattttat 18739940

PHAS26: 661     gggccgcacgcgcgctacactgatgtattcaacgagcttatagccttggccgacaggccc 720
                | |||||||||||||||||||||||||||||| ||||||||||||| ||| |||||| ||
Sbjct: 18739939 gagccgcacgcgcgctacactgatgtattcaatgagcttatagcctcggctgacaggacc 18739880

PHAS26: 721     gggtaatctttgaaatttcatcgtgatggggatagatcattgcaattgttggtcttcaac 780
                |||||||||||||||||||||||||||||||||| |||||||||||||||||||||||||
Sbjct: 18739879 gggtaatctttgaaatttcatcgtgatggggataaatcattgcaattgttggtcttcaac 18739820

PHAS26: 781     gaggaattcctagtaggcgcgagtcatcagctcgcgttgactacgtccctgccctttgta 840
                |||||||| |||||| ||| ||||||  |||| |||||||| |||||| |||||||| ||
Sbjct: 18739819 gaggaatttctagtaagcgtgagtcacaagcttgcgttgacaacgtccttgccctttata 18739760

PHAS26: 841     cacaccgcccgtcgctcctaccgattgaatgatccggtgaaatgttcggatcgcggcgac 900
                ||||||||||||||||||||||  |||||||||| |||||||||||||||||||||||||
Sbjct: 18739759 cacaccgcccgtcgctcctaccatttgaatgatctggtgaaatgttcggatcgcggcgac 18739700

PHAS26: 901     gtgggcggttcgctgcccgcgacgtcgcgagaagtccattgaaccttatcatttagagga 960
                |||||||||||||||||| | ||||| |||||||||||||| ||||||| ||||||||||
Sbjct: 18739699 gtgggcggttcgctgcccacaacgtcacgagaagtccattggaccttattatttagagga 18739640

PHAS26: 961     aggagaagtcgtaacaaggtttccgtaggtggacctgcggaaggatcattgtcgaaacct 1020
                ||||||||||||||||||||||||||||||| ||||| ||||||||||||| ||||||||
Sbjct: 18739639 aggagaagtcgtaacaaggtttccgtaggtgaacctgtggaaggatcattgccgaaacct 18739580
```

Genome-wide identification and characterization of phased small interfering RNA genes in response to Botrytis cinerea infection in Solanum lycopersicum
Fangli Wu, Yue Chen, Xing Tian, Xiaole Zhu, Weibo Jin

Table S1 Sequences of the 91 PHASs identified via PhaseTank pipeline.

```
//
>PHAS11 excise_beg:3823470	excise_end:3824094	excise_len:625	Phased_ratio:0.457	Phased_num:14	phased_abun:865
...AGAAGGCAATGTAGCTGAGGC...
...TAATTTGAAGAACTCACGAGA...
...
...
...
[illegible]
[illegible]
...CGCAAACACGTGTGTACGGAA...
...AGACGGAACTACAGTTCTCAT...
...CGGACAACGCAGAGTTGCTAT...
...TCACAAACATACAAGGTTTCT...
...TCTCGTTACATGGAGTTTACT...
...CTTTTAGCTGACTTGAACTAA...
...
...
...

//
>PHAS05 excise_beg:33139124	excise_end:33139433	excise_len:310	Phased_ratio:0.528	Phased_num:4	phased_abun:1052
[illegible]
[illegible]
...AGGGCTGTTAAAGTTCGTGAG...
...ATGAACAAGCGATAGCCAGAG...
...AGCGGTCATAAATCGGAACCT...
...CCACGCTGTCCCAGGCCCGTG...

//
>PHAS31 excise_beg:2704350	excise_end:2704554	excise_len:205	Phased_ratio:0.315	Phased_num:4	phased_abun:139
...GGATAAGCATATGGATTGGTG...
[illegible]
[illegible]
...ACCGTTATACACTTAAGAAAC...
...GACAAAGGCAGAGTTCAAGAA...
...ACAAGATGAGTTCCAGCGTCT...

//
>PHAS72 excise_beg:2717464	excise_end:2717710	excise_len:247	Phased_ratio:0.353	Phased_num:8	phased_abun:397
...AGGTATACACGTGTAGCAGTG...
...TGAAGATATATGAACATGAGG...
...ATATTCCTTGCTCACTGTTTA...
...ATACTTTAGCTCATGACCTGG...
[illegible]
[illegible]
...ACTTAGACAAGACCGTCTCAT...
...ATAGGGTAAGAGTTTAGGCTT...
...CCTATAAGGAACGAGTGACAA...
...ATTATGAAATCGAGTACTGGA...

//
>PHAS53 excise_beg:11186857	excise_end:11187145	excise_len:289	Phased_ratio:0.953	Phased_num:5	phased_abun:1978
...TGATTAGGCTGTGGATTGCTG...
...AGCAATTCATAAAGGCAAGAA...
...TGGCAGAGGAGTATCTACAAG...
...TTTTGGCTGGTAAACAAAGGG...
[illegible]
[illegible]
...AACGTTTTAAGTACTAGAAGA...

//
>PHAS09 excise_beg:990328	excise_end:990679	excise_len:352	Phased_ratio:0.975	Phased_num:5	phased_abun:124
...ACAAGAGACAAAGATATCACA...
...TATTATCTGAACTCGCGGTTA...
[illegible]
[illegible]
...AATCATACTGATGTTCAGAGA...
...TTGGAATTATAGTGTTAGGTT...
...TACGGGAAGGTTTTGTCGGTT...

//
>PHAS55 excise_beg:13623048	excise_end:13623420	excise_len:373	Phased_ratio:0.902	Phased_num:7	phased_abun:278
...TTCGAGACGTGATTATCATTC...
...TACTGCCGACTTTTGAGTCAC...
...ATTCAGGAAGGACAAGTTTCA...
...AGAGTGATCGCAGGTTGTCCT...
...AAACGTAGACCAGTCCAGAAC...
[illegible]
[illegible]
...GACAAGGCAGTCAGGTTAAAC...
...ACTTTGCATCTGGTCAGGTCT...

//
>PHAS24 excise_beg:18645430	excise_end:18645718	excise_len:289	Phased_ratio:0.326	Phased_num:7	phased_abun:1372
...CTTGCGACCATACTCCCCCCG...
...CCCTGGTCGGCATCGTTTATG...
[illegible]
[illegible]
...GCAGTTAAGGAAATTCAAAGT...
...CGGAACGCTGGTATGAGGGGG...
...GCCTTGGGTTTTTGAAACTAA...
...TAGGGACCAGCCGTAGCAAAT...
...GACTAGTAGAAGCTCGGGGGT...

//
>PHAS10 excise_beg:63987877	excise_end:63988291	excise_len:415	Phased_ratio:0.587	Phased_num:7	phased_abun:245
...TTATGAAATTCAGGAAAAGGT...
[illegible]
[illegible]
...GTGTGAAAGCTCGATAGGAAA...
...AAAGCTGTAGGAGATCAAGTA...
...GACCCGTTGCAGGAAGAGGCT...
...GGGTTGAAAGAAGACTAGAAT...
...AGCGTCATAGGGTGTACAGTA...
...CAGGTGTATCAG

//
```

```
>PHAS48 excise_beg:54263089     excise_end:54263986     excise_len:898  Phased_ratio:0.455      Phased_num:17   phased_abun:1238
............................................................TGCGGGTAAGACAACACTAGC......................................................................................................................................................................................................................................................................
.................................................................................TAAAGCGGTTTACAATGATGA.....................................................................................................................................................................................................................................................
..........................................................................................................................................................................................TCAAGAAATTGGCTCATTTGA........................................................................................................................................
...................................................................................................................................................................................................................................................................................................TGATGTGTGGAATGATGACTA...............................
.......................................................................................................................................................................................................................................................................................................................................................
.......................................................................................................................................................................................................................................................................................................................................................
.......................................................................................................................................................................................................................................................................................................................................................
.......................................................................................................................................................................................................................................................................................................................................................
.......................................................................................................................................................................................................................................................................................................................................................
.......................................................................................................................................................................................................................................................................................................................................................
.......................................................................................................................................................................................................................................................................................................................................................
.......................................................................................................................................................................................................................................................................................................................................................
ATTGTCTGAAGATGCAAGTGGACGAAACCTCACTGTAGTTCCTATTGTAGGAATGGGTGGTGCGGGTAAGACAACACTAGCTAAAGCGGTTTACAATGATGACAAGGTGAAGAATCATTTTAGTTTGAAAGGTTGGTATTGTGTTTCTGAGGCATATGATGCTTTGAGAATAACCAAAGGTTTACTTCAAGAAATTGGCTCATTTGAGCCAAAGGATGACGGTAATTTTAATAAGCTACAAGTCAAATTGAAGGAAAGCCTGAAGGGAAAGAAGTTCCTTGTTGTCCTCGATGATGTGTGGAATGATGACTATAGTGAGTGGACCAACCTGAGAAATGTTTT
TAACAGACTTCTACGTTCACCTGCTTTGGAGTGACATCAAGGATAACATCCTTACCCACCACGCCATTCTGTTGTGATCGATTTCGCCAAATGTTACTACTGTTCCACTTCTTAGTAAAATCAAACTTTCCAACCATAACACAAAGACTCCGTATACTACGAAACTCTTATTGGTTTCCAAATGAAGTTCTTTAACCGAGTAAACTCGGTTTCCTACTGCCATTAAAATTATTCGATGTTCAGTTTAACTTCCTTTCGGACTTCCCTTTCTTCAAGGAACAACAGGAGCTACTACACCTTACTACTGATATCACTCACCCTGTTGGACTCTTTACAAAA
.............................................................................................................................................................................................................CTCGGTTTCCTACTGCCATTA.....................................................................................................................
..................................................................................................................................................................................................................................AAATTATTCGATGTTCAGTTT................................................................................................
.......................................................................................................................................................................................................................................................................................................................................................
.......................................................................................................................................................................................................................................................................................................................................................
.......................................................................................................................................................................................................................................................................................................................................................



//
>PHAS13 excise_beg:2161857      excise_end:2162313      excise_len:457  Phased_ratio:0.773      Phased_num:12   phased_abun:227
......................................................................................................TTGATCTTCTAGCGCAGACAC............................................................................................................................................................................................................................
...............................................................................................................................................................................................................ATTGCTCTCAAAGGTACGGAA...................................................................................................................
.....................................................................................................................................................................................................................................TTGAGGTCCTTGAGGGATGCA.............................................................................................
..........................................................................................................................................................................................................................................................TCCTTGGAGATAATTGTGGGA........................................................................
.......................................................................................................................................................................................................................................................................................................................................................
CGAGATCAAGAAATATATTATGGATATGCCAATACACAGTCCACTTCCATAGCCCATCAGAGCTGCTTTCCAAAAATCATTGAAAAATTTAGAATTGCTTTCTTGATCTTCTAGCGCAGACACTGTATAGTTTGTCTCTGACACAGGATCTTTGCCACAACCTTTTGAAACTGGATATCCACGTAATCCATCATTACCTTCATATGAATTGCTCTCAAAGGTACGGAATTGAGGTCCTTGAGGGATGCATCCTTGGAGATAATTGTGGGAGATTTAGGAATTCAAGAAACGTAAGAGAAGCAAGTTGTTGTGGTATCTCTCCAGAAAGTTGGTTAAACGAA
GCTCTAGTTCTTTATATAATACCTATACGGTTATGTGTCAGGTGAAGGTATCGGGTAGTCTCGACGAAAGGTTTTTAGTAACTTTTTAAATCTTAACGAAAGAACTAGAAGATCGCGTCTGTGACATATCAAACAGAGACTGTGTCCTAGAAACGGTGTTGGAAAACTTTGACCTATAGGTGCATTAGGTAGTAATGGAAGTATACTTAACGAGAGTTTCCATGCCTTAACTCCAGGAACTCCCTACGTAGGAACCTCTATTAACACCCTCTCTAAATCCTTAAGTTCTTTGCATTCTCTTCGTTCAACAACACCATAGAGAGGTCTTTCAACCAATTTGCTT
..........................................................TCTCGACGAAAGGTTTTTAGT........................................................................................................................................................................................................................................................................
...............................................................................AACTTTTTAAATCTTAACGAA...................................................................................................................................................................................................................................................
........................................................................................................................................................................................TTAGGTAGTAATGGAAGTATA..........................................................................................................................................
.................................................................................................................................................................................................................................................................................................TTGCATTCTCTTCGTTCAACA.................................
...........................................................................................................................................................................................................................................................................................................................................ACCAATTTGCTT
.......................................................................................................................................................................................................................................................................................................................................................
.......................................................................................................................................................................................................................................................................................................................................................



//
>PHAS59 excise_beg:66918286     excise_end:66918700     excise_len:415  Phased_ratio:0.595      Phased_num:8    phased_abun:361
............................................................ATATGCGATGACATGACTGGC......................................................................................................................................................................................................................................................................
..........................................................................................................................................................................................AATGAATTCCACGGTTCTCAT........................................................................................................................................
....................................................................................................................................................................................................................................TTCTCGAGCTTGACACATCTT..............................................................................................
CCTATCCAAAAACGAATTCATGGAACATGAGTAGAGATTGTTGTTTATGGGATGGAGTCATATGCGATGACATGACTGGCCATGTCATTGAACTTGACCTCGGTTGCAGCCGTCTTGTTGGGACAATTGATTCCAATAGCAGCCTATTCCAACTCTCTCATCTCCAAAGACTCAACCTTTCTTGGAATGAATTCCACGGTTCTCATATCTCGCCTGAATTTGGTAGGTTCTCGAGCTTGACACATCTTGATCTTTACAACTCCAATTTCTCAGGTCAAATTCCTTCTGAAATCTCTCATCTTTCCAAGTTACACTCTCTTCGTCTCTATGCTACTGGTAGTC
GGAATAGGTTTTTGCTTAAGTACCTTGTACTCATCTCTAACAACAAATACCCTACCTCAGTATACGCTACTGTACTGACCGGTACAGTAACTTGAACTGGAGCCAACGTCGGCAGAACAACCCTGTTAACTAAGGTTATCGTCGGATAAGGTTGAGAGAGTAGAGGTTTCTGAGTTGGAAAGAACCTTACTTAAGGTGCCAAGAGTATAGAGCGGACTTAAACCATCCAAGAGCTCGAACTGTGTAGAACTAGAAATGTTGAGGTTAAAGAGTCCAGTTTAAGGAAGACTTTAGAGAGTAGAAAGGTTCAATGTGAGAGAAGCAGAGATACGATGACCATCAG
....................................................................................................AGCCAACGTCGGCAGAACAAC..............................................................................................................................................................................................................................
..............................................................................................................................................CGGATAAGGTTGAGAGAGTAG....................................................................................................................................................................................
...................................................................................................................................................................AGGTTTCTGAGTTGGAAAGAA...............................................................................................................................................................
......................................................................................................................................................................................................................................................................................................................ATGTGAGAGAAGCAGAGATAC............
...........................................................................................................................................................................................................................................................................................................................................GATGACCATCAG



//
>PHAS77 excise_beg:4340342      excise_end:4340651      excise_len:310  Phased_ratio:0.775      Phased_num:4    phased_abun:102
............................................................TATGGATTGCAGAAGGTTTCA....................................................................................................................................................................................................................................
.................................................................................TAAGGACTTGTGAAAATGGTA...............................................................................................................................................................................................................
................................................................................................................................................TTAGCAGGAACTTGTTACAGG................................................................................................................................................
....................................................................................................................................................................................................................................TCTGTTTGACAGAAGCTGAAA............................................................
TTCTATCTATGAGTAGTTTCCAAGAGGATTTTCATTTTGAGACTCAGATATTGATACAATTATGGATTGCAGAAGGTTTCATAAGGACTTGTGAAAATGGTAAAGTTTGGAGGAAGTGGCAATAGATTATTTGGAGGACCTTATTAGCAGGAACTTGTTACAGGCTAGAGAAAGGAGATTCAATGGTGAGATAAAAACATGCGGAATACATGATCTACTGCGTGAGTTCTGTTTGACAGAAGCTGAAATGACAAAGCATATGCATGTTGAGAGAACTTACCCTACTCTTCCAACACAAATGCATAATG
AAGATAGATACTCATCAAAGGTTCTCCTAAAAGTAAAACTCTGAGTCTATAACTATGTTAATACCTAACGTCTTCCAAAGTATTCCTGAACACTTTTACCATTTTCAAACCTCCTTCACCGTTATCTAATAAACCTCCTGGAATAATCGTCCTTGAACAATGTCCGATCTCTTTCCTCTAAGTTACCACTCTATTTTTGTACGCCTTATGTACTAGATGACGCACTCAAGACAAACTGTCTTCGACTTTACTGTTTCGTATACGTACAACTCTCTTGAATGGGATGAGAAGGTTGTGTTTACGTATTAC



//
>PHAS62 excise_beg:1125523      excise_end:1125895      excise_len:373  Phased_ratio:0.508      Phased_num:7    phased_abun:998
.....................................................................................................................................................................TTTTCAGATTGCAGATAGATG.............................................................................................................................................................
...............................................................................................................................................................................................................TATTGTGTGAAAGGAGAAGGA...................................................................................................................
..........................................................................................................................................................................................................................................................GGAGTGAATTCGGAATAGGAC........................................................................
AGCTAATGGCCTTGAAAGAGTTTCCAATACTAAAAGTTGTATTGATTGTCCCACTAAGTCTGTTGTTGCTCAAATCCAAATCCAAAAGGTATTCGTTCCTCTCACCCACGCATTGTGGGATTGTTCCCTCCAAATTGTTACTACCCAAGTCTAACACCATCAATGTTTTCAGATTGCAGATAGATGATGAAATATATCCACTGATATTATTGTGTGAAAGGAGAAGGAATTGTAGGCTCTCCTGGTTTAGGAGTGAATTCGGAATAGGACCTTCCAGCTGATTTTGTTTTAGAGTAACGGTACTTAATGTTTTGGACTTGAACTCTTGAATTTTTCCACTGAA
TCGATTACCGGAACTTTCTCAAAGGTTATGATTTTCAACATAACTAACAGGGTGATTCAGACAACAACGAGTTTAGGTTTAGGTTTTCCATAAGCAAGGAGAGTGGGTGCGTAACACCCTAACAAGGGAGGTTTAACATGATGGGTTCAGATTGTGGTAGTTACAAAAGTCAACGTCTATCTACTACTTTATATAGGTGACTATAATAACACACTTTCCTCTCCTTAACATCCGAGAGGACCAAATCCTCACTTAAGCCTTATCCTGGAAGGTCGACTAAAACAAAATCTCATTGCCATGAATTACAAAACCTGAACTTGAGAACTTAAAAAGGTGACT
..........................................................AGACAACAACGAGTTTAGGTT........................................................................................................................................................................................................................................................................
...............................................................................TAGGTTTTCCATAAGCAAGGA...................................................................................................................................................................................................................................................
..............................................................................................................................................TGGGTTCAGATTGTGGTAGTT....................................................................................................................................................................................
.................................................................................................................................................................................................................................................................................................ATCTCATTGCCATGAATTACA.................................



//
>PHAS67 excise_beg:4209688      excise_end:4209997      excise_len:310  Phased_ratio:0.602      Phased_num:5    phased_abun:1155
............................................................CAGCGGCTGGAAGAGCACCGC....................................................................................................................................................................................................................................
.....................................................................................................................................................................TAGAAGCGACGCATGCGCCCG...................................................................................................................
..........................................................................................................................................................................................CCGTCCGCTTGCCGACCCGCA......................................................................................................
....................................................................................................................................................................................................................................GGGCACGTGTCGTTGGCTAAG............................................................
GTTATCTTTTCTGTTTAACAGCCTGCCCACCCTGGAATCGGCTCAGCCGGAGGTAGGGTCCAGCGGCTGGAAGAGCACCGCACGTCGCGCTTGGTTGAAAAGCCAGTGGCGCGAAGCTACCGTGTGCTGGATTATGACTGAACGCCTCTAAATCAGAATCCGGGCTAGAAGCGACGCATGCGCCCGCGTCCGCTTGCCGACCCGCAGTAAGGGCCTATGGCCCCTAAGGGCACGTGTCGTTGGCTAAGTCGCCGCGACGGAAGCGTCGCGGTGACCGCCTTGACGACTAAAGTTGATATTTAAATATT
CAATAGAAAAGACAAATTGTCGGACGGGTGGGACCTTAGCCGAGTCGGCCTCCATCCCAGGTCGCCGACCTTCTCGTGGCGTGCAGCGCGAACCAACTTTTCGGTCACCGCGCTTCGATGGCACACGACCTAATACTGACTTGCGGAGATTTAGTCTTAGGCCCGATCTTCGCTGCGTACGCGGGCGGCAGGCGAACGGCTGGGCGTCATTCCCGGATACCGGGGATTCCCGTGCACAGCAACCGATTCAGCGGCGCTGCCTTCGCAGCGCCACTGGCGGAACTGCTGATTTCAACTATAAATTTATAA
..................................................................................................................................................................................................................................TTCCCGTGCACAGCAACCGAT..............................................................



//
>PHAS42 excise_beg:51458570     excise_end:51459425     excise_len:856  Phased_ratio:0.545      Phased_num:21   phased_abun:7460
............................................................TCAATTAATGGAGTTTGTGTA......................................................................................................................................................................................................................................................................
.................................................................................CAGAGGTCTGAAGGGTAGGAG.....................................................................................................................................................................................................................................................
...........................................................................................................................TATTTGGAGTACTGAGGCATG.......................................................................................................................................................................................................
..............................................................................................................................................................................................................................................................................AAGTCGAGATGATAGTTGGAA....................................................
...................................................................................................................................................................................................................................................................................................TCTATTCATCGAAAGACTATT...............................
.........................................................................................................................................................................................................................................................................................................................CAAAAAAGATTTGTGTCCTCC.........
..............................................................................................................................................................................................................................................................................................................................................TTTACTTGTA
.......................................................................................................................................................................................................................................................................................................................................................
.......................................................................................................................................................................................................................................................................................................................................................
.......................................................................................................................................................................................................................................................................................................................................................
.......................................................................................................................................................................................................................................................................................................................................................
.......................................................................................................................................................................................................................................................................................................................................................
ACAAATGCTTCTTGAAGTTGCCTCTTCGATTACCGAAAGCAATCAAGAAATGAGTGATGATCAATTAATGGAGTTTGTGTACAGAGGTCTGAAGGGTAGGAGATTTCTAATTGTCATAGATGATATTTGGAGTACTGAGGCATGGGACCTAATGCAAACAATATGTCCAAATGATGACAATAAAAGCCGAATTCTATTAACTACTCGGCTCAAGTATGTTGCTGATTATGTCAACTGCCCTGATTTTCCGCCTCATAGTAAGTCTTTGCTAAGTCGAGATGATAGTTGGAATCTATTCATCGAAAGACTATTCAAAAAAGATTTGTGTCCTCCTTTACTTGTA
TGTTTACGAAGAACTTCAACGGAGAAGCTAATGGCTTTCGTTAGTTCTTTACTCACTACTAGTTAATTACCTCAAACACATGTCTCCAGACTTCCCATCTCTAAAGATTAACAGTATCTACTATAAACCTCATGACTCCGTACCCTGGATTACGTTTGTTATACAGGTTTACTACTGTTATTTTCGGCTTAAGATAATTGATGAGCCGAGTTCATACAACGACTAATACAGTTGACGGGACTAAAAGGCGGAGTATCATTCAGAAACGATTCAGCTCTACTATCAACCTTAGATAAGTAGCTTTCTGATAAGTTTTTTCTAAACACAGGAGGAAATGAACAT
........................................................................................................................................................................................TCGGCTTAAGATAATTGATGA..........................................................................................................................................
.............................................................................................................................................................................................................GCCGAGTTCATACAACGACTA.....................................................................................................................
..................................................................................................................................................................................................................................ATACAGTTGACGGGACTAAAA................................................................................................
........................................................................................................................................................................................................................................................GGCGGAGTATCATTCAGAAAC..........................................................................
......................................................................................................................................................................................................................................................................................................................AAGTTTTTTCTAAACACAGGA............
.......................................................................................................................................................................................................................................................................................................................................................
.......................................................................................................................................................................................................................................................................................................................................................
.......................................................................................................................................................................................................................................................................................................................................................
.......................................................................................................................................................................................................................................................................................................................................................



//
>PHAS84 excise_beg:18430364     excise_end:18430673     excise_len:310  Phased_ratio:0.573      Phased_num:4    phased_abun:105
TGGGATATTGCTTTTCAACAAGGAGCAGGATGCACAAAGCTGCTATCACTCTTTCAAAAGCAATAACAAATCTTCAGTTGATTCATTTCTTACCTCATTCTAGGTGGTTTACTTTTAAGCTATCACAAGCATATCGATTTAGGTCAACAATCCATTTATTTAAAAGTTGAAAACTGTAAACATTAGCATCCAGACACAGCCTAGTAGACATTTCACTAAACAAAAATAGAGATCCGAGAGCTTCTTTTCTCGCTTTAGTTCATTGACCTCCATAACACCATTGTCACTTGATTTGATAGAATCTGATTC
ACCCTATAACGAAAAGTTGTTCCTCGTCCTACGTGTTTCGACGATAGTGAGAAAGTTTTCGTTATTGTTTAGAAGTCAACTAAGTAAAGAATGGAGTAAGATCCACCAAATGAAAATTCGATAGTGTTCGTATAGCTAAATCCAGTTGTTAGGTAAATAAATTTTCAACTTTTGACATTTGTAATCGTAGGTCTGTGTCGGATCATCTGTAAAGTGATTTGTTTTTATCTCTAGGCTCTCGAAGAAAAGAGCGAAATCAAGTAACTGGAGGTATTGTGGTAACAGTGAACTAAACTATCTTAGACTAAG
..........................................................TCGTTATTGTTTAGAAGTCAA......................................................................................................................................................................................................................................
..............................................................................................................................................CAGTTGTTAGGTAAATAAATT..................................................................................................................................................
........................................................................................................................................................................................TCGTAGGTCTGTGTCGGATCA........................................................................................................
..................................................................................................................................................................................................................................ATCTCTAGGCTCTCGAAGAAA..............................................................
```

```
//
>PHAS87 excise_beg:3344007	excise_end:3344274	excise_len:268	Phased_ratio:0.327	Phased_num:5	phased_abun:194
.................................................................................TCCTGCACATGTCATCGAGGA......................................................................................................................................................................
...........................................................................................................................AAATTTGAGCTTATGATTATA............................................................................................................................
.....................................................................................................................................................................GCATTAACACTAGGACTCGGG..................................................................................
..........................................................................................................................................................................................TCAATTTTTCTTGAGACGGCC.............................................................
CAAAAATAAAAAAGCAAGCAATATGCAGGGCATTCGTCTAGGGAAAGATCTTGAATCACTAACAATGCCTAGTATATAAAATCCTGCACATGTCATCGAGGAAATTGCAAGGAGTTGCCAAATAAATTTGAGCTTATGATTATAGGGTCTTGTGATGTGCTGTTTGCATTAACACTAGGACTCGGGTCAATTTTTCTTGAGACGGCCTGTCGACATATACGCAAGCTTCCTTGGGATTTGATGAAATTCGATTGCAATAACAACAAC
GTTTTTATTTTTTCGTTCGTTATACGTCCCGTAAGCAGATCCCTTTCTAGAACTTAGTGATTGTTACGGATCATATATTTTAGGACGTGTACAGTAGCTCCTTTAACGTTCCTCAACGGTTTATTTAAACTCGAATACTAATATCCCAGAACACTACACGACAAACGTAATTGTGATCCTGAGCCCAGTTAAAAAGAACTCTGCCGGACAGCTGTATATGCGTTCGAAGGAACCCTAAACTACTTTAAGCTAACGTTATTGTTGTTG
..........................................................GATTGTTACGGATCATATATT.............................................................................................................................................................................................


//
>PHAS36 excise_beg:2992369	excise_end:2992699	excise_len:331	Phased_ratio:0.659	Phased_num:6	phased_abun:124
............................................................TGGACCTTAGTTGTAGTCAGC..........................................................................................................................................................................................................................................................
..........................................................................................................................................................................................TTGGCCGATTGACGAATTTGA............................................................................................................................
.........................................................................................................................................................................................................................................................CAACAGAAATCTCATACCTTT.............................................................
AAGATTGTTGTACTTGGGACGGAGTTACTTGTGACGTGTTAACCGGTCATGTTATTGGCCTGGACCTTAGTTGTAGTCAGCTTGTTGGCAATTTTCATCCCAATAGCAGTCTCTTCCAACTTCATCATCTGCAGACACTAAATCTTGCTTACAATGACTTTTATCCTTCTTTGATCCCACATAACATTGGCCGATTGACGAATTTGAGGCATCTCAACCTTTCTTATTTTACCTTCGATGGGAAAATCCCAACAGAAATCTCATACCTTTCTAATTTGGTTTCCCTTGATCTTTCTTATGGTTTTGGATTACAACTTAATGAGAGAACAT
TTCTAACAACATGAACCCTGCCTCAATGAACACTGCACAATTGGCCAGTACAATAACCGGACCTGGAATCAACATCAGTCGAACAACCGTTAAAAGTAGGGTTATCGTCAGAGAAGGTTGAAGTAGTAGACGTCTGTGATTTAGAACGAATGTTACTGAAAATAGGAAGAAACTAGGGTGTATTGTAACCGGCTAACTGCTTAAACTCCGTAGAGTTGGAAAGAATAAAATGGAAGCTACCCTTTTAGGGTTGTCTTTAGAGTATGGAAAGATTAAACCAAAGGGAACTAGAAAGAATACCAAAACCTAATGTTGAATTACTCTCTTGTA
.........................................................................................................................AGTAGTAGACGTCTGTGATTT.............................................................................................................................................................................................
...................................................................................................................................................................AGGAAGAAACTAGGGTGTATT...................................................................................................................................................
.......................................................................................................................................................................................................................................................GGGTTGTCTTTAGAGTATGGA...............................................................


//
>PHAS44 excise_beg:2778979	excise_end:2779351	excise_len:373	Phased_ratio:0.687	Phased_num:15	phased_abun:4968
............................................................AGTAAGCATCAGAAAGATTGA......................................................................................................................................................................................................................................................................
...........................................................................................................................TAGAAGGATAGAAGTAGTTCT.......................................................................................................................................................................................................
................................................................................................................................................TAGCAAGGTTTAGTGTCCGGA..................................................................................................................................................................
.....................................................................................................................................................................GATGATGAAGTTGGATGAGGC.............................................................................................................................................
..........................................................................................................................................................................................TGCTGTTGGGATAAATAGTTC........................................................................................................................
...............................................................................................................................................................................................................CATTAAGCCGACTACAACTAA...................................................................................................
....................................................................................................................................................................................................................................GGTCCAATCCGATAACGTGAC..............................................................................
AAAGATCAAGTGAAACCAGATTGGAAAGGTATGAGATTTCTGTTGGGATTTCCCCTTGAAAGTAAGCATCAGAAAGATTGAGATGCCTCAAATTCCTCAACCGGCTAACGTCATTTGGGATCGTAGAAGGATAGAAGTAGTTCTTAGCAAGGTTTAGTGTCCGGAGATGATGAAGTTGGATGAGGCTGCTGTTGGGATAAATAGTTCCATTAAGCCGACTACAACTAAGGTCCAATCCGATAACGTGACCGGTTAACATGTCACAAGTGACCCCGTCCCAAGTGCAGCAATCGTTACTCTCATTCCATGAAACTGTTTTCGGGAATGACGTATCACACGGGGA
TTTCTAGTTCACTTTGGTCTAACCTTTCCATACTCTAAAGACAACCCTAAAGGGGAACTTTCATTCGTAGTCTTTCTAACTCTACGGAGTTTAAGGAGTTGGCCGATTGCAGTAAACCCTAGCATCTTCCTATCTTCATCAAGAATCGTTCCAAATCACAGGCCTCTACTACTTCAACCTACTCCGACGACAACCCTATTTATCAAGGTAATTCGGCTGATGTTGATTCCAGGTTAGGCTATTGCACTGGCCAATTGTACAGTGTTCACTGGGGCAGGGTTCACGTCGTTAGCAATGAGAGTAAGGTACTTTGACAAAAGCCCTTACTGCATAGTGTGCCCCT
..........................................................TTTCATTCGTAGTCTTTCTAA........................................................................................................................................................................................................................................................................
...............................................................................CTCTACGGAGTTTAAGGAGTT...................................................................................................................................................................................................................................................
........................................................................................................................................................................................CGACGACAACCCTATTTATCA..........................................................................................................................................
.............................................................................................................................................................................................................AGGTAATTCGGCTGATGTTGA.....................................................................................................................
..................................................................................................................................................................................................................................TTCCAGGTTAGGCTATTGCAC................................................................................................
.......................................................................................................................................................................................................................................................TGGCCAATTGTACAGTGTTCA...........................................................................
............................................................................................................................................................................................................................................................................CTGGGGCAGGGTTCACGTCGT......................................................
.................................................................................................................................................................................................................................................................................................TAGCAATGAGAGTAAGGTACT.................................


//
>PHAS41 excise_beg:66333637	excise_end:66334345	excise_len:709	Phased_ratio:0.643	Phased_num:21	phased_abun:1973
............................................................TCCCTTTGGATGTCTTCTTGT......................................................................................................................................................................................................................................................................
.................................................................................TCAGTAAGTTTTGAAGCATGT.................................................................................................................................................................................................................................................
......................................................................................................AAGATGCTAGCTCAGAAGATG............................................................................................................................................................................................................................
...........................................................................................................................CCTAGGCTTAATGTGGAAGTC.......................................................................................................................................................................................................
................................................................................................................................................ATAGATGAGAGGGGTCCTCCA..................................................................................................................................................................
.....................................................................................................................................................................GATACAAGGCCAGAAAGCTGC.............................................................................................................................................
...............................................................................................................................................................................................................TATCGAACAGTAGCTGGACGA...................................................................................................
....................................................................................................................................................................................................................................AGATTTGATACACCTGGTTAT..............................................................................
..............................................................................................................................................................................................................................................................................GCAGCTGTAAGCTTGACCTGA....................................................
........................................................................................................................................................................................................................................................................................................................TCCAGCAAGACTTCACATGGA..........
.............................................................................................................................................................................................................................................................................................................................................AAGATGGGCA
GATTGTCCGTTTGGAGACAAGGCTCTCTTGGCTAATGCTGCAAAGCTCGAGACAATGCGATCCCTTTGGATGTCTTCTTGTTCAGTAAGTTTTGAAGCATGTAAGATGCTAGCTCAGAAGATGCCTAGGCTTAATGTGGAAGTCATAGATGAGAGGGGTCCTCCAGATACAAGGCCAGAAAGCTGCCCTGTGGAGAAACTTTACATATATCGAACAGTAGCTGGACGAAGATTTGATACACCTGGTTATGTTTGGACAATGGATGAAGATGCAGCTGTAAGCTTGACCTGAGCCATACATCAAATGGGAAATTCCAGCAAGACTTCACATGGAAAGATGGGCA
CTAACAGGCAAACCTCTGTTCCGAGAGAACCGATTACGACGTTTCGAGCTCTGTTACGCTAGGGAAACCTACAGAAGAACAAGTCATTCAAAACTTCGTACATTCTACGATCGAGTCTTCTACGGATCCGAATTACACCTTCAGTATCTACTCTCCCCAGGAGGTCTATGTTCCGGTCTTTCGACGGGACACCTCTTTGAAATGTATATAGCTTGTCATCGACCTGCTTCTAAACTATGTGGACCAATACAAACCTGTTACCTACTTCTACGTCGACATTCGAACTGGACTCGGTATGTAGTTTACCCTTTAAGGTCGTTCTGAAGTGTACCTTTCTACCCGT
...............................................................................CAAGTCATTCAAAACTTCGTA...................................................................................................................................................................................................................................................
....................................................................................................CATTCTACGATCGAGTCTTCT..............................................................................................................................................................................................................................
.........................................................................................................................ACGGATCCGAATTACACCTTC.........................................................................................................................................................................................................
...................................................................................................................................................................GTCTATGTTCCGGTCTTTCGA...............................................................................................................................................
.................................................................................................................................................................................................................................................................................................CTCGGTATGTAGTTTACCCTT.................................
.......................................................................................................................................................................................................................................................................................................................................................
.......................................................................................................................................................................................................................................................................................................................................................
.......................................................................................................................................................................................................................................................................................................................................................
.......................................................................................................................................................................................................................................................................................................................................................
.......................................................................................................................................................................................................................................................................................................................................................


//
>PHAS90 excise_beg:58954978	excise_end:58955287	excise_len:310	Phased_ratio:0.318	Phased_num:4	phased_abun:181
............................................................ACAGGAAATGGAGTCGAGCCG.....................................................................................................................................................................................................................................
................................................................................................................................................TATTTCTCGAAGACATTCTGT.................................................................................................................................................
....................................................................................................................................................................................................................................TTAATTGACTGTCGGTCAACA.............................................................
AATTCATATAATCCTCAATAAGTTTCGGCGAAACTTGATCTGTAATGCCATATCCTTCGAACAGGAAATGGAGTCGAGCCGTATCCTTCAATTTCTCTTTTCCCTTCTCGCTTGACCCAAAGAATTACAATATGGAGATTGAATATTTCTCGAAGACATTCTGTTTCTATGCGCAGGAGTGACTGAATCGGTTCTAACTCCAATCTCAGCTATTGGAATGTCAGTGGTTAATTGACTGTCGGTCAACAAGTACTGTTGCTAGTTATATTCTGTGGATTGTAAGAATATAATGGTCTAGCATTAGTAGG
TTAAGTATATTAGGAGTTATTCAAAGCCGCTTTGAACTAGACATTACGGTATAGGAAGCTTGTCCTTTACCTCAGCTCGGCATAGGAAGTTAAAGAGAAAAGGGAAGAGCGAACTGGGTTTTCTTAATGTTATACCTCTAACTTATAAAGAGCTTCTGTAAGACAAAGATACGCGTCCTCACTGACTTAGCCAAGATTGAGGTTAGAGTCGATAACCTTACAGTCACCAATTAACTGACAGCCAGTTGTTCATGACAACGATCAATATAAGACACCTAACATTCTTATATTACCAGATCGTAATCATCC
.............................................................................................................................................................................................................GAGTCGATAACCTTACAGTCA....................................................................................


//
>PHAS54 excise_beg:87425595	excise_end:87425883	excise_len:289	Phased_ratio:0.907	Phased_num:6	phased_abun:735
TCTACTCTTCTTTTTGTTGTTTTTTGCTGGTGGGGACTCCCTCTGCGAATACTGACGGACCTTCTTACCATGGGTCAGTATTATGGAAAGACCGTCCCCACCGTCCGCAATTCTGATGGAGCAATCACGGATTAATCAGTGATTCCGATCATCCGTCGCCGCAGACGCCAGTCAGCAGCAACAATTTGCCGTCTGTGAAAGACACTCCGGCTCGATCCTCTGCCAACGGTCCTTGACCTAGCCCTTACCCTCATGGTATAGTTGGTCAAAAGCTCCCACAAATTAGTA
AGATGAGAAGAAAAACAACAAAAAACGACCACCCCTGAGGGAGACGCTTATGACTGCCTGGAAGAATGGTACCCAGTCATAATACCTTTCTGGCAGGGGTGGCAGGCGTTAAGACTACCTCGTTAGTGCCTAATTAGTCACTAAGGCTAGTAGGCAGCGGCGTCTGCGGTCAGTCGTCGTTGTTAAACGGCAGACACTTTCTGTGAGGCCGAGCTAGGAGACGGTTGCCAGGAACTGGATCGGGAATGGGAGTACCATATCAACCAGTTTTCGAGGGTGTTTAATCAT
..........................................................TGGAAGAATGGTACCCAGTCA..................................................................................................................................................................................................
...............................................................................TAATACCTTTCTGGCAGGGGT.............................................................................................................................................................................
....................................................................................................CAGGCGTTAAGACTACCTC
```

```
//
>PHAS75 excise_beg:705061	excise_end:705328	excise_len:268	Phased_ratio:0.493	Phased_num:4	phased_abun:2433
.........................................................................TAGCTTGACGTATAGAACAAA..................................
.................................................................................TTTCATATATCATAACGACTA..........................
AAATGTTTCTAATGGAGGCATAGTGATGAAGTTCATCAGAATCAATCAAGTGATTAACGGGTACAATCCTTGCTCTATCTCTAGCTTGACGTATAGAACAAATTTCATATATCATAACGACTACTATCACCGGGACAACATGT[illegible]
TTTACAAAGATTACCTCCGTATCACTACTTCAAGTAGTCTTAGTTAGTTCACTAATTGCCCATGTTAGGAACGAGATAGAGATCGAACTGCATATCTTGTTTAAAGTATATAGTATTGCTGATGATAGTGGCCCTGTTGTACA[illegible]
....................................................CCCATGTTAGGAACGAGATAG.......................................................
.............................................................................................................CAGTTTTATAATACGGTGGTT..........

//
>PHAS52 excise_beg:37335120	excise_end:37335597	excise_len:478	Phased_ratio:0.367	Phased_num:14	phased_abun:1408
..........................................................................................TCGCAGTTCTGGATAAGTGGC.....................
......................................................................................................CAAAGGAAAAGGTCCAGCTGC.........
[illegible]
[illegible]
....................................................CCTGGTATACACAGTCCTCAT...........................................................
..................................................................................AGTCGACTTAGTTGTCCTTCT...............................
..........................................................................................CCAGCGTCAAGACCTATTCAC.....................
...................................................................................................CGGCCGCCACCTTGTTGGCGA............
.........................................................................................................CCGTTTCCTTTTCCAGGTCGA.......
...................................................................................................................TTAAATGTTTACTATGGTTTA...
.......................................................................................................................AATGTCACGGGACAAAAGTAT
..............................................................................................................................ATCACACGCGTAAGGAAAAGT...
....................................................................................................................................AAGACACTGCTT

//
>PHAS15 excise_beg:37325903	excise_end:37326401	excise_len:499	Phased_ratio:0.854	Phased_num:11	phased_abun:34411
..........................................................................................TGAAAAGGAATGTGCACACTA.....................
...................................................................................................TATGAAAACAGGGCACTGTAA...........
..................................................................................................................................AGCTGGACCTTTTCCTTTGCC...
[illegible]
[illegible]
....................................................GCGATAGAGGTAACTAAACAC...........................................................
.........................................................................................................TTTAAACCATAGTGGACATTT.......
..............................................................................................................AAATTGGTAGGTGTCGGTCGA....
...................................................................................................................CCACACGTTCAAGGTCGTAAT.
.......................................................................................................................CGTCGACCTGGAAAAGGAAAC
..............................................................................................................................CGGTGAATAGGTCTTGACGTT....

//
>PHAS73 excise_beg:491972	excise_end:492344	excise_len:373	Phased_ratio:0.614	Phased_num:4	phased_abun:863
...................................................................................................................TTTGGATTTGAGCAACAACAG...
.........................................................................................................................AACTTTTAGTATTGGAAACTC
[illegible]
[illegible]
....................................................CTTCCAGGATAAGGCTTAAGT...........................................................
..................................................................................AGTAGATAGACGTTAGACTTT...............................

//
>PHAS85 excise_beg:29736845	excise_end:29737175	excise_len:331	Phased_ratio:0.306	Phased_num:5	phased_abun:640
..........................................................................AAGGTGATTACTGCGGGACAA.....................................
..............................................................................................................AGAAGATTGGGTTTTGGACTA.......
...................................................................................................................TTAAACAAAGTTGCATTGTTA....
[illegible]
[illegible]
....................................................AACTTTTTGACACGTATGGT............................................................
..........................................................................................CTCAAGATTTCGCACCAAGAT.....................

//
>PHAS19 excise_beg:38677752	excise_end:38678040	excise_len:289	Phased_ratio:0.363	Phased_num:4	phased_abun:2614
.........................................................................CATGCTAATGTATACAGAGCG.......................................
..........................................................................................TTAAGGCCAGGAGCGCATCGC.....................
....................................................................................................CGGTGCACACCTAGGGCGGAC............
[illegible]
[illegible]
....................................................TGGCTTTAGGATATTACAATA...........................................................

//
>PHAS88 excise_beg:9815931	excise_end:9816387	excise_len:457	Phased_ratio:0.307	Phased_num:6	phased_abun:103
.....................................................TAAGGCCACAGGAGTTGATAA..........................................................
..................................................................................ATACGCAATTCTAAACAGATT...............................
...................................................................................................................AGAAAGAATATTGGACATTGA...
[illegible]
[illegible]
.............................................................................................CACTAGCTAGTTCTATCACTA..................

//
>PHAS26 excise_beg:18740259	excise_end:18740610	excise_len:352	Phased_ratio:0.647	Phased_num:5	phased_abun:10225
.....................................................CCGGCGGAGTCCTAAAAGCAA..........................................................
[illegible]
[illegible]
....................................................ACGGCCGCCTCAGGATTTCG............................................................
..........................................................................................AACTAATTACTTTTGTAGGAA.....................
.........................................................................................................AGCAGAAAGTATTTAGGTTCT.......
...................................................................................................................GACAATTAGTAATGAGGCTAG...

//
>PHAS47 excise_beg:53108590	excise_end:53109361	excise_len:772	Phased_ratio:0.460	Phased_num:17	phased_abun:1248
.....................................................AAGAGATTGGCTCATTTGACT..........................................................
..........................................................................................ATGTGTGGAATGATGACTATA.....................
..............................................................................................................TGGGAAGTAAGATCATCGTAA.......
..............................................................................................................................GAATCTGTCTAGTGAAGACTC..
....................................................................................................................................TTGGGCTTTA
[illegible]
[illegible]
.......................................................................GAGTTTCCTACTGCCGTTAAA........................................
..................................................................................CTTCAAAGAACAACAGGAACT...............................
...................................................................................................................ACGAGCCTGCTACACACGACT...
```

```
......

//
>PHAS89 excise_beg:32069642	excise_end:32069888	excise_len:247	Phased_ratio:0.334	Phased_num:4	phased_abun:223
......TAAACTAAGAAACAATGTGCA......
......AGGAATATCCACATGTTTGTA......
......AACAGATTCACTGCCACACGT......
ACGTTATGTGAAGTAGAAAGACAATCCATTGGCTAAAGAATTGTTGCTTACCATTTCTTTCAACTGTTCCAGGTCATGAGCTAAACTAAGAAACAATGTGCAAGGAATATCCACATGTTTGTATATCTTCAACTTCGGATTAGAGAATGGGATATACTCCGCCAGAACAGATTCACTGCCACACGTGTATACCTTCATTTGCAAAGGCAGATCACAATTAGAGGAACCAAATATACATCTAAACGT
TGCAATACACTTCATCTTTCTGTTAGGTAACCGATTTCTTAACAACGAATGGTAAAGAAAGTTGACAAGGTCCAGTACTCGATTTGATTCTTTGTTACACGTTCCTTATAGGTGTACAAACATATAGAAGTTGAAGCCTAATCTCTTACCCTATATGAGGCGGTCTTGTCTAAGTGACGGTGTGCACATATGGAAGTAAACGTTTCCGTCTAGTGTTAATCTCCTTGGTTTATATGTAGATTTGCA
......AAGTTGACAAGGTCCAGTACT......

//
>PHAS02 excise_beg:1897637	excise_end:1898156	excise_len:520	Phased_ratio:0.476	Phased_num:9	phased_abun:1635
......GGGACTGAGACACGGCCCAGA......
......AAAGCCTGACGGAGCAATGCC......
......AACTCTGTGCCAGCAGCCGCG......
......GTAATACAGAGGATGCAAGCG......
......TTATCCGGAATGATTGGGCGT......
......AAAGCGTCTGTAGGTGGCTTT......
......
......
......
AGGCAATAGCTTACCAAGGCGATGATCAGTAGCTGGTCCGAGAGGATGATCAGCCACACTGGGACTGAGACACGGCCCAGACTCCTACGGGAGGCAGCAGTGGGGAATTTTCCGCAATGGGCGAAAGCCTGACGGAGCAATGCCGCGTGGAGGTAGAAGGCCCACGGGTCGTGAACTTCTTTTCCCGGAGAAGAAGCAATGACGGTATCTGGGGAATAAGCATCGGCTAACTCTGTGCCAGCAGCCGCGGTAATACAGAGGATGCAAGCGTTATCCGGAATGATTGGGCGTAAAGCGTCTGTAGGTGGCTTTTTAAGTCCGCCGTCAAATCCCAGGGCTCAAC
TCCGTTATCGAATGGTTCCGCTACTAGTCATCGACCAGGCTCTCCTACTAGTCGGTGTGACCCTGACTCTGTGCCGGGTCTGAGGATGCCCTCCGTCGTCACCCCTTAAAAGGCGTTACCCGCTTTCGGACTGCCTCGTTACGGCGCACCTCCATCTTCCGGGTGCCCAGCACTTGAAGAAAAGGGCCTCTTCTTCGTTACTGCCATAGACCCCTTATTCGTAGCCGATTGAGACACGGTCGTCGGCGCCATTATGTCTCCTACGTTCGCAATAGGCCTTACTAACCCGCATTTCGCAGACATCCACCGAAAAATTCAGGCGGCAGTTTAGGGTCCCGAGTTG

//
>PHAS38 excise_beg:54896607	excise_end:54897252	excise_len:646	Phased_ratio:0.709	Phased_num:15	phased_abun:2731
......ATGAGAGAGTGCAGCCAAGTG......
......TCCGGAACTTGAAGAGGTCGG......
......TAAGACGCTCGCTGGCATGTT......
......TGAGTGGAAATGTATTTTGAG......
......AAGTGAAATATGGGAGCTGCG......
......
......
......
AGGGAAAAAATCTGGCTGCAGTTCCTATTGTTGGAATGGGCGGCTTGGGTAAGACAACACTTGCTAAAGCCGCTTACAATGATGAGAGAGTGCAGCCAAGTGCAAAGGACATCCTATGGGACATCCGGAACTTGAAGAGGTCGGAAGACAAATTGCAGCCAAGTGCAAAGGACTGCCCTTAGCTCTTAAGACGCTCGCTGGCATGTTACGCCCCAAATCAGAGATTGATGAGTGGAAATGTATTTTGAGAAGTGAAATATGGGAGCTGCGAGACAATGACATATTACCAGCGTTAATGTTGAGCTACAATGATCTTCCCGCACATTTAAAGCGATGCTTTTCT
TCCCTTTTTTAGACCGACGTCAAGGATAACAACCTTACCCGCCGAACCCATTCTGTTGTGAACGATTTCGGCGAATGTTACTACTCTCTCACGTCGGTTCACGTTTCCTGTAGGATACCCTGTAGGCCTTGAACTTCTCCAGCCTTCTGTTTAACGTCGGTTCACGTTTCCTGACGGGAATGAGAATTCTGCGAGCGACCGTACAATGCGGGGTTTAGTCTCTAACTACTCACCTTTACATAAAACTCTTCACTTTATACCCTCGACGCTCTGTTACTGTATAATGGTCGCAATTACAACTCGATGTTACTAGAGGGCGTGTAAATTTCGCTACGAAAAGA
......TGAACGATTTCGGCGAATGTT......
......AATGCGGGGTTTAGTCTCTAA......
......GCTCTGTTACTGTATAATGGT......
......CGCAATTACAACTCGATGTTA......
......
......
......

//
>PHAS22 excise_beg:2347115	excise_end:2347403	excise_len:289	Phased_ratio:0.875	Phased_num:5	phased_abun:200
......TTCTACGTTCGTGCTTGGATT......
......CATAGAGAAATGTTGTTGGGC......
......AAAAGTACTGAGCAATTGGCA......
......AAAGGAAGGAGATATCTCATT......
GGGATTGGCAAAACGACTCTTGCTAGAAAAGTTTATGATGATATTTACATGGAGCATCACTTCTACGTTCGTGCTTGGATTACTGTATCTCAAATGCATCAACATAGAGAAATGTTGTTGGGCATCTTGCGGTGTTTTTCACTGGTCAATGACTACACTTACCTAAAAAGTACTGAGCAATTGGCAGAACAAGTTTACAGAAGTTTGAAAGGAAGGAGATATCTCATTGCAATGGATGATGTGTGGGACACCACTGCATGGGATGTCGTGAAAAGATCTTTTCCAGAT
CCCTAACCGTTTGCTGAGAACGATCTTTTCAAATACTACTATAAATGTACCTCGTAGTGAAGATGCAAGCACGAACCTAATGACATAGAGTTTACGTAGTTGTATCTCTTTACAACAACCCGTAGAACGCCACAAAAAGTGACCAGTTACTGATGTGAATGGATTTTTCATGACTCGTTAACCGTCTTGTTCAAATGTCTTCAAACTTTCCTTCCTCTATAGAGTAACGTTACCTACTACACACCCTGTGGTGACGTACCCTACAGCACTTTTCTAGAAAAGGTCTA
......TGAAGATGCAAGCACGAACCT......

//
>PHAS66 excise_beg:30150665	excise_end:30150890	excise_len:226	Phased_ratio:0.634	Phased_num:4	phased_abun:4744
......CGGATTCTGACTTAGAGGCGT......
AAAGAAAGAAAACGTGCAACATTTATTAAGTTGATGGAAGGCTCCTAAAAAGTTAAATATAGCCAACGACACGTGCCCTTAGGGGCCATAGGCCCCTGCTGCGGGTCGGCAAGCGGACGGCGGGCGCATGCGTCGCTTCTAGCCCGGATTCTGACTTAGAGGCGTTCAGTCATAATCCAGCACACGATAGCTTCGCGCCACTGGCTTTTTAACCAAGCGTGATGG
TTTCTTTCTTTTGCACGTTGTAAATAATTCAACTACCTTCCGAGGATTTTTCAATTTATATCGGTTGCTGTGCACGGGAATCCCCGGTATCCGGGGACGACGCCCAGCCGTTCGCCTGCCGCCCGCGTACGCAGCGAAGATCGGGCCTAAGACTGAATCTCCGCAAGTCAGTATTAGGTCGTGTGCTATCGAAGCGCGGTGACCGAAAAATTGGTTCGCACTACC
......TATCGGTTGCTGTGCACGGGA......
......CCCGCGTACGCAGCGAAGATC......
......GGGCCTAAGACTGAATCTCCG......

//
>PHAS29 excise_beg:54529614	excise_end:54529902	excise_len:289	Phased_ratio:0.625	Phased_num:5	phased_abun:335
......TTTGGTTTGAAAGCTTGGTAT......
......TGTGTTTCTGAGGTATATGAT......
......TTACTTCAAGAAATTGGATCA......
......TTTGACTCAAAGGATGACGGC......
GGTGCGGGTAAGACAACACTAGCTAAAGCGGTTTACAATGATGCCAAGGTGAAGAACCATTTTGGTTTGAAAGCTTGGTATTGTGTTTCTGAGGTATATGATGCTTTGAGAATAACAAAAGGATTACTTCAAGAAATTGGATCATTTGACTCAAAGGATGACGGCAATTTTAAAGGAAAACCTGAAGGGAAAGAAGTTCCTTGTTGTCCTTGATGATGTGTGGAATGATACCTATAGTGAGTGGGATAACCTGAGAAATGTTTTTGTCCAAGGAGATATGGGAAGTAA
CCACGCCCATTCTGTTGTGATCGATTTCGCCAAATGTTACTACGGTTCCACTTCTTGGTAAAACCAAACTTTCGAACCATAACACAAAGACTCCATATACTACGAAACTCTTATTGTTTTCCTAATGAAGTTCTTTAACCTAGTAAACTGAGTTTCCTACTGCCGTTAAAATTTCCTTTTGGACTTCCCTTTCTTCAAGGAACAACAGGAACTACTACACACCTTACTATGGATATCACTCACCCTATTGGACTCTTTACAAAAACAGGTTCCTCTATACCCTTCATT
......CAGGAACTACTACACACCTTA......

//
>PHAS04 excise_beg:33137543	excise_end:33138062	excise_len:520	Phased_ratio:0.454	Phased_num:10	phased_abun:742
......GCGGCTTAATTTGACTCAACA......
......GACATAGTAAGGATTGACAGA......
......GTTCTTAGTTGGTGGAGCGAT......
......AGGGACTACGGCCTTTTAGGC......
......CGCGGAAGTTTGAGGCAATAA......
......CAGGTCTGTGATGCCCTTAGA......
......
......
NNNNNNNNNNNNNNNNNNNNNNNNNGGAATTGACCGAAGGGAACCACCAGGAGTGGAGCCTGCGGCTTAATTTGACTCAACACGGGGAAACTTACCAGGTCCAGACATAGTAAGGATTGACAGACTGAGAGCTCTTTCTTGATTCTATGGGTGGTGGTGCATGGTCGTTCTTAGTTGGTGGAGCGATTTGTCTTGTTAATTCCGTTAACGAATGAGACCTCAGCCTGCTAACTAGCTATGCGGAGGTATCCCTTCGCGGCAAGCTTCTTAGAGGGACTACGGCCTTTTAGGCCGCGGAAGTTTGAGGCAATAACAGGTCTGTGATGCCCTTAGATGTTCTGGGC
NNNNNNNNNNNNNNNNNNNNNNNNNCCTTAACTGGCTTCCCTTGGTGGTCCTCACCTCGGACGCCGAATTAAACTGAGTTGTGCCCCTTTGAATGGTCCAGGTCTGTATCATTCCTAACTGTCTGACTCTCGAGAAAGAACTAAGATACCCACCACCACGTACCAGCAAGAATCAACCACCTCGCTAAACAGAACAATTAAGGCAATTGCTTACTCTGGAGTCGGACGATTGATCGATACGCCTCCATAGGGAAGCGCCGTTCGAAGAATCTCCCTGATGCCGGAAATCCGGCGCCTTCAAACTCCGTTATTGTCCAGACACTACGGGAATCTACAAGACCCG
......
......

//
>PHAS79 excise_beg:33695519	excise_end:33695807	excise_len:289	Phased_ratio:0.594	Phased_num:4	phased_abun:362
......ATGAGTGGGATGACTTGAGAA......
......TCGGAAGTAAGATCATTGTAA......
......TATTCAAACGACATTCACTAG......
GGAAATCATAAGGAAAAAAGGTTTTTGTTGTCCTTGATGATGTGCGGAATGATAACTATAATGAGTGGGATGACTTGAGAAATACTTTTGTACAAAGAGATTTCGGAAGTAAGATCATTGTAACTACATATAAGGAGAATGTTTCCTTGATTATGGGTGGAGGAGCAATCAACATGGGAACTCTATCTAGTGAAGTCTCTTGGGCTTTATTCAAACGACATTCACTAGAAAACAGGGATCATGAGTAATATACAAAATTTGAAGAGATTGGAAGAAAAATTGCTGACA
CCTTTAGTATTCCTTTTTTCCAAAAACAACAGGAACTACTACACGCCTTACTATTGATATTACTCACCCTACTGAACTCTTTATGAAAACATGTTTCTCTAAAGCCTTCATTCTAGTAACATTGATGTATATTCCTCTTACAAAGGAACTAATACCCACCTCCTCGTTAGTTGTACCCTTGAGATAGATCACTTCAGAGAACCCGAAATAAGTTTGCTGTAAGTGATCTTTTGTCCCTAGTACTCATTATATGTTTTAAACTTCTCTAACCTTCTTTTTAACGACTGT
......AAATAAGTTTGCTGTAAGTGA......

//
>PHAS16 excise_beg:37334710	excise_end:37335040	excise_len:331	Phased_ratio:0.319	Phased_num:6	phased_abun:555
......GTAGTTTGGGATGGCACTGGA......
......GAAGATTGGCATGGCACTGGA......
CATGAAGATCCAAATTTGCAATCCCATACTTCATGTAATAATGACATTCAGGCTGCCCAGGTCTTTCCGGAAATGCAAGTCCATCCTGGATGCTGCTTGACGCACCACGAGAAGCAGTAAGAGATGCATATGGGCTTGCGTAGGCAGGTGCTGAAGCAGATAGTCCAGTATGACCATAAATTGGACCTGCTCCAGCTGCAGACTGAGTAGTTTGGGATGGCACTGGACCTGTTCCAGCTGCAGAGTGAAGATTGGCATGGCACTGGACCTGCTCCAGCTGCAGACTGAGTAGTTTGGTATTGCACTGGACTAGTAGAACCCTGCACC
GTACTTCTAGGTTTAAACGTTAGGGTATGAAGTACATTATTACTGTAAGTCCGACGGGTCCAGAAAGGCCTTTACGTTCAGGTAGGACCTACGACGAACTGCGTGGTGCTCTTCGTCATTCTCTACGTATACCCGAACGCATCCGTCCACGACTTCGTCTATCAGGTCATACTGGTATTTAACCTGGACGAGGTCGACGTCTGACTCATCAAACCCTACCGTGACCTGGACAAGGTCGACGTCTCACTTCTAACCGTACCGTGACCTGGACGAGGTCGACGTCTGACTCATCAAACCATAACGTGACCTGATCATCTTGGGACGTGG
......GTCCAGAAAGGCCTTTACGTT......
......TGCGTGGTGCTCTTCGTCATT......
......CTCTACGTATACCCGAACGCA......
......CTGGACAAGGTCGACGTCTCA......

//
>PHAS60 excise_beg:2986223	excise_end:2986595	excise_len:373	Phased_ratio:0.913	Phased_num:5	phased_abun:372
```

```
............................................................TGAGGAATTTGAGGCATCTCA.......................................................................................................................................................................................................................................................................
.........................................................................................................................................................................................................................................................TTGGCAACATCTCATCACCCA..........................................................................
CACTAAACCTTGCTTACAATGACTTTTATCCTTCTTCGATCCCACAGAACATTAGCCAATTGAGGAATTTGAGGCATCTCAACCTTTCTGATGCTTGGTTTCAAGGGAAAATCCCAACAGAAATATCATACCTTTCCAATTTGGTTTCACTTGATCTTTCTAATAGTTATCATAGATTACAACTTGATGAGAGAACATTTGAAACAATGCTTCACAACTTGACAAATCTTGAGTTACTAGCTCTCTCTCTTGGCAACATCTCATCACCCATTCATCCCAATAGCAGCCTCTTCCAGCTTCATCATCTCCACACACTAAACCTTTATAACGATTACTTTCCTCC
GTGATTTGGAACGAATGTTACTGAAAATAGGAAGAAGCTAGGGTGTCTTGTAATCGGTTAACTCCTTAAACTCCGTAGAGTTGGAAAGACTACGAACCAAAGTTCCCTTTTAGGGTTGTCTTTATAGTATGGAAAGGTTAAACCAAAGTGAACTAGAAAGATTATCAATAGTATCTAATGTTGAACTACTCTCTTGTAAACTTTGTTACGAAGTGTTGAACTGTTTAGAACTCAATGATCGAGAGAGAGAACCGTTGTAGAGTAGTGGGTAAGTAGGGTTATCGTCGGAGAAGGTCGAAGTAGTAGAGGTGTGTGATTTGGAAATATTGCTAATGAAAGGAGG
.........................................................................................................................TTATAGTATGGAAAGGTTAAA..........................................................................................................................................................................................................
.............................................................................................................................................................................................................TTACGAAGTGTTGAACTGTTT......................................................................................................................
.................................................................................................................................................................................................................................................................................................GAAGGTCGAAGTAGTAGAGGT..................................



//
>PHAS18 excise_beg:3097032      excise_end:3097299      excise_len:268  Phased_ratio:0.977      Phased_num:6    phased_abun:379
......................................................................................................ATCAATAGACTTGCTACTACT.................................................................................................................................................
AATAATTGTAAGAACTCAAAAAACACTTACCCTCCACCTTTTCAACCGATCAATAGGTGCACTCTTAGTCCTTGGAATATCAAAAGGACTTCCACAATCATCATCAATAGACTTGCTACTACTTCCAGCTTCCAAATCCTCATTCTGATGCCGCCTATACGGCGACCCTTTCACATTCTCTTCACTCATCGTTTCCACTTCCAAAATTCCTCTACTGTCGCAGGAGAGATGACACCAGATTTGACAATAATTTTTTTCTCAGATTTT
TTATTAACATTCTTGAGTTTTTTGTGAATGGGAGGTGGAAAAGTTGGCTAGTTATCCACGTGAGAATCAGGAACCTTATAGTTTTCCTGAAGGTGTTAGTAGTAGTTATCTGAACGATGATGAAGGTCGAAGGTTTAGGAGTAAGACTACGGCGGATATGCCGCTGGGAAAGTGTAAGAGAAGTGAGTAGCAAAGGTGAAGGTTTTAAGGAGATGACAGCGTCCTCTCTACTGTGGTCTAAACTGTTATTAAAAAAAGAGTCTAAAA
..........................................................CGTGAGAATCAGGAACCTTAT.............................................................................................................................................................................................
...............................................................................AGTTTTCCTGAAGGTGTTAGT........................................................................................................................................................................
.........................................................................................................................GAAGGTCGAAGGTTTAGGAGT..............................................................................................................................
...................................................................................................................................................................CTGGGAAAGTGTAAGAGAAGT....................................................................................
........................................................................................................................................................................................GAGTAGCAAAGGTGAAGGTTT...............................................................



//
>PHAS58 excise_beg:844677       excise_end:845112       excise_len:436  Phased_ratio:0.306      Phased_num:13   phased_abun:1421
...............................................................................................................................................................................................................TCCGAAAATTGTGGCATAGCC....................................................................................................................
.........................................................................................................................................................................................................................................................AGTCCTTCCAGCGTCGAGTTC..........................................................................
...................................................................................................................................................................................................................................................................................................CAACCAATGGCTTGATTCGCT................................
GAGCTACCTCCTTCACTAACTGTTTTTTTAGCCAAATCTCTCCATTTTGTAGCATTTTTTCTAATCTCATCACTCTTTTCTCCTTCCATTACCTCTTTTAAACAAAACAACAACTCTTCTCTTTTGACAATTCCTAACATGTCATCCAACTTAGGTCTCACACCAATCTCCCATATCTCATCAATAAACTTTGCATCCGTCATTTGATCCGAAAATTGTGGCATAGCCACCATTGGCACGCCGAGGCTTAGTCCTTCCAGCGTCGAGTTCCATCCACAGTGAGTTACAAAACAACCAATGGCTTGATTCGCTAGCGTCTCGAGCTGATTGCACCATGACACAA
CTCGATGGAGGAAGTGATTGACAAAAAAATCGGTTTAGAGAGGTAAAACATCGTAAAAAAGATTAGAGTAGTGAGAAAAGAGGAAGGTAATGGAGAAAATTTGTTTTGTTGTTGAGAAGAGAAAACTGTTAAGGATTGTACAGTAGGTTGAATCCAGAGTGTGGTTAGAGGGTATAGAGTAGTTATTTGAAACGTAGGCAGTAAACTAGGCTTTTAACACCGTATCGGTGGTAACCGTGCGGCTCCGAATCAGGAAGGTCGCAGCTCAAGGTAGGTGTCACTCAATGTTTTGTTGGTTACCGAACTAAGCGATCGCAGAGCTCGACTAACGTGGTACTGTGTT
..........................................................AAGATTAGAGTAGTGAGAAAA.........................................................................................................................................................................................................................................................................
...............................................................................GAGGAAGGTAATGGAGAAAAT....................................................................................................................................................................................................................................................
...................................................................................................................................................................GTTAGAGGGTATAGAGTAGTT................................................................................................................................................................
........................................................................................................................................................................................ATTTGAAACGTAGGCAGTAAA...........................................................................................................................................
.............................................................................................................................................................................................................CTAGGCTTTTAACACCGTATC......................................................................................................................
.......................................................................................................................................................................................................................................................AATCAGGAAGGTCGCAGCTCA............................................................................
............................................................................................................................................................................................................................................................................ATGGTAGGTGTCACTCAATGTT.......................................................
......................................................................................................................................................................................................................................................................................................................GATCGCAGAGCTCGACTAACG.............
............................................................................................................................................................................................................................................................................................................................................TGGTACTGTGTT
........................................................................................................................................................................................................................................................................................................................................................



//
>PHAS01 excise_beg:88580778     excise_end:88581066     excise_len:289  Phased_ratio:0.600      Phased_num:5    phased_abun:464
.................................................................................TCCAGAGGCTTTGGACGGATG...........................................................................................................................................................................................
ATTGACGACTTTGTCATCCCGGACATTCTGCCCGAAGTGGCAAAAAGTCATCTGCTGGTTTTGACGAGTCTCGCAAGCTTTTCCAGAGGCTTTGGACGGATGAAGACGAAATTGAGCTGTTGCGTGGATTTTTGGACTACACCACCCAGCGGGGCTTGAACACGTCTTCCCCGCAGCACCATTACGACACGACTGCTTTTTATGAGCAGATCAAGAGCAAATTTCAGCTGGATTTTAACAAAAATCAGCTTGTGGAAGTCAATGACTATTCATAGCTATTACCTTGAT
TAACTGCTGAAACAGTAGGGCCTGTAAGACGGGCTTCACCGTTTTTCAGTAGACGACCAAAACTGCTCAGAGCGTTCGAAAAGGTCTCCGAAACCTGCCTACTTCTGCTTTAACTCGACAACGCACCTAAAAACCTGATGTGGTGGGTCGCCCCGAACTTGTGCAGAAGGGGCGTCGTGGTAATGCTGTGCTGACGAAAAATACTCGTCTAGTTCTCGTTTAAAGTCGACCTAAAATTGTTTTTAGTCGAACACCTTCAGTTACTGATAAGTATCGATAATGGAACTA
..........................................................AAAACTGCTCAGAGCGTTCGA..................................................................................................................................................................................................................
...............................................................................AAAGGTCTCCGAAACCTGCCT.............................................................................................................................................................................................
........................................................................................................................................................................................GCTGTGCTGACGAAAAATACT....................................................................................
.............................................................................................................................................................................................................CGTCTAGTTCTCGTTTAAAGT...............................................................



//
>PHAS12 excise_beg:423568       excise_end:424045       excise_len:478  Phased_ratio:0.944      Phased_num:19   phased_abun:46628
............................................................CTCTTATAGCTATGGCAAGAA.......................................................................................................................................................................................................................................................................
...........................................................................................................................ATCACTACTTCTCCCAATATC........................................................................................................................................................................................................
...............................................................................................................................................................................................................TCTTTGCTCTAAGGCCACATA....................................................................................................................
....................................................................................................................................................................................................................................TCAAATGTTTGTGTTGGATTG...............................................................................................
.........................................................................................................................................................................................................................................................TAATGATGATTAAACAACCCG..........................................................................
..............................................................................................................................................................................................................................................................................AACCAAACCAACTTCTTTCTC.....................................................
...................................................................................................................................................................................................................................................................................................TTGTTAAGGACTCTAATCGGC................................
........................................................................................................................................................................................................................................................................................................................TTAGATGATCCACGTCATCAA...........
........................................................................................................................................................................................................................................................................................................................................................
........................................................................................................................................................................................................................................................................................................................................................
ACAAAATGTATATGCCTAATACTTTCATGTTTAAGCTAAGTAGAGAAATGGAGAACTTGCCTCTTATAGCTATGGCAAGAAGTTGCAATCGTCTCCTTATTGTCAGAGTTTTCATTCTGCTTCATCACTACTTCTCCCAATATCAACTCTTCTTTCTTTAAAATACAAGATTTCTTGAAATCTTTAGAATATCTAGCAACCAATTAGTCTTTGCTCTAAGGCCACATATCAAATGTTTGTGTTGGATTGTAATGATGATTAAACAACCCGAACCAAACCAACTTCTTTCTCTTGTTAAGGACTCTAATCGGCTTAGATGATCCACGTCATCAAGAATAATTAA
TGTTTTACATATACGGATTATGAAAGTACAAATTCGATTCATCTCTTTACCTCTTGAACGGAGAATATCGATACCGTTCTTCAACGTTAGCAGAGGAATAACAGTCTCAAAAGTAAGACGAAGTAGTGATGAAGAGGGTTATAGTTGAGAAGAAAGAAATTTTATGTTCTAAAGAACTTTAGAAATCTTATAGATCGTTGGTTAATCAGAAACGAGATTCCGGTGTATAGTTTACAAACACAACCTAACATTACTACTAATTTGTTGGGCTTGGTTTGGTTGAAGAAAGAGAACAATTCCTGAGATTAGCCGAATCTACTAGGTGCAGTAGTTCTTATTAATT
...............................................................................TTCAACGTTAGCAGAGGAATA....................................................................................................................................................................................................................................................
....................................................................................................ACAGTCTCAAAAGTAAGACGA...............................................................................................................................................................................................................................
.........................................................................................................................AGTAGTGATGAAGAGGGTTAT..........................................................................................................................................................................................................
..............................................................................................................................................AGTTGAGAAGAAAGAAATTTT.....................................................................................................................................................................................
........................................................................................................................................................................................ATCTTATAGATCGTTGGTTAA...........................................................................................................................................
.............................................................................................................................................................................................................TCAGAAACGAGATTCCGGTGT......................................................................................................................
..................................................................................................................................................................................................................................ATAGTTTACAAACACAACCTA.................................................................................................
............................................................................................................................................................................................................................................................................GCTTGGTTTGGTTGAAGAAAG.......................................................
........................................................................................................................................................................................................................................................................................................................................................



//
>PHAS07 excise_beg:356043       excise_end:356919       excise_len:877  Phased_ratio:0.606      Phased_num:33   phased_abun:19615
.................................................................................GAGAAAATCAGACAAATGGCG..................................................................................................................................................................................................................................................
......................................................................................................AAAAAAGAAAGGTTGTTTAAA.............................................................................................................................................................................................................................
.....................................................................................................................................................................AGAATTCAGGATGAGATTGCA..............................................................................................................................................................
.........................................................................................................................................................................................................................................................AGGTTAATGGATCAGAACAGT..........................................................................
...................................................................................................................................................................................................................................................................................................GATGTTTGGAAGGCTCTTGAA................................
........................................................................................................................................................................................................................................................................................................................CTAGAGAAACTTGGAATTCCT...........
........................................................................................................................................................................................................................................................................................................................................................
........................................................................................................................................................................................................................................................................................................................................................
........................................................................................................................................................................................................................................................................................................................................................
........................................................................................................................................................................................................................................................................................................................................................
........................................................................................................................................................................................................................................................................................................................................................
........................................................................................................................................................................................................................................................................................................................................................
........................................................................................................................................................................................................................................................................................................................................................
........................................................................................................................................................................................................................................................................................................................................................
........................................................................................................................................................................................................................................................................................................................................................
........................................................................................................................................................................................................................................................................................................................................................
GTCATGGCAGCTTTGAGAGATGACGAGGTTGCTATTATTGGGATATGTGGTATGGGTGGAGTTGGTAAGACAACACTGACTGAGAAAATCAGACAAATGGCGAAAAAAGAAAGGTTGTTTAAAGATGTTGTCATGGTAATTGTCAGTCAACAAATAGACTTTAAAAGAATTCAGGATGAGATTGCAGGAGGAGTTGGGCTGACATTAGAAGGGGATGATTTGTGGAGTCGTGGAGATCTGCTGCGTACAAGGTTAATGGATCAGAACAGTCGCATCCTTATAATCTTGGATGATGTTTGGAAGGCTCTTGAACTAGAGAAACTTGGAATTCCTAGTGGTAGCA
CAGTACCGTCGAAACTCTCTACTGCTCCAACGATAATAACCCTATACACCATACCCACCTCAACCATTCTGTTGTGACTGACTCTTTTAGTCTGTTTACCGCTTTTTTCTTTCCAACAAATTTCTACAACAGTACCATTAACAGTCAGTTGTTTATCTGAAATTTTCTTAAGTCCTACTCTAACGTCCTCCTCAACCCGACTGTAATCTTCCCCTACTAAACACCTCAGCACCTCTAGACGACGCATGTTCCAATTACCTAGTCTTGTCAGCGTAGGAATATTAGAACCTACTACAAACCTTCCGAGAACTTGATCTCTTTGAACCTTAAGGATCACCATCGT
..........................................................CTCAACCATTCTGTTGTGACT.........................................................................................................................................................................................................................................................................
...............................................................................GACTCTTTTAGTCTGTTTACC....................................................................................................................................................................................................................................................
....................................................................................................GCTTTTTTCTTTCCAACAAAT...............................................................................................................................................................................................................................
.........................................................................................................................TTCTACAACAGTACCATTAAC..........................................................................................................................................................................................................
..............................................................................................................................................AGTCAGTTGTTTATCTGAAAT.....................................................................................................................................................................................
...................................................................................................................................................................TTTCTTAAGTCCTACTCTAAC................................................................................................................................................................
........................................................................................................................................................................................GTCCTCCTCAACCCGACTGTA...........................................................................................................................................
.............................................................................................................................................................................................................ATCTTCCCCTACTAAACACCT......................................................................................................................
..................................................................................................................................................................................................................................CAGCACCTCTAGACGACGCAT.................................................................................................
.......................................................................................................................................................................................................................................................GTTCCAATTACCTAGTCTTGT............................................................................
............................................................................................................................................................................................................................................................................CAGCGTAGGAATATTAGAACC.......................................................
........................................................................................................................................................................................................................................................................................................................................................
........................................................................................................................................................................................................................................................................................................................................................
........................................................................................................................................................................................................................................................................................................................................................
........................................................................................................................................................................................................................................................................................................................................................
........................................................................................................................................................................................................................................................................................................................................................
........................................................................................................................................................................................................................................................................................................................................................



//
>PHAS27 excise_beg:1182678      excise_end:1183680      excise_len:1003 Phased_ratio:0.500      Phased_num:20   phased_abun:2880
............................................................AACTCTGAAACAGTGGAATAA.......................................................................................................................................................................................................................................................................
...............................................................................................................................................................................................................................................................................TCACTTAGGCATAGCTGGCGA....................................................
....................................................................................................................................................................................................................................................................................................AGAAGATCATGAATTTTGCAA...............................
........................................................................................................................................................................................................................................................................................................................................................
........................................................................................................................................................................................................................................................................................................................................................
........................................................................................................................................................................................................................................................................................................................................................
........................................................................................................................................................................................................................................................................................................................................................
........................................................................................................................................................................................................................................................................................................................................................
```

```
GGTATTACACAAGAGAAATCGTAATATACTGATGATACGTCCAACACCTTAAGCAACTTGAACTCTGAAACAGTGGAATAAGTCTTTAAATATTGCGTTGAAATAAAGGTACGAGTTATACTTGTTATATCATTGCTATGCGCTGGACGATAAACTTGCTTCTCTTTATGTTTATGCAGACCTATCACTCGCCGCTGATCATCTATTACTTCTACTGAAGACACGACGACATTCCCATTGATTACATGCACAACATTTTCAGTATGAGTTTCACTTAGGCATAGCTGGCGAAGAAGATCATGAATTTTGCAACTTCTCATCCTTCCATTAGCACTTTGTTTAC
CCATAATGTGTTCTCTTTAGCATTATATGACTACTATGCAGGTTGTGGAATTCGTTGAACTTGAGACTTTGTCACCTTATTCAGAAATTTATAACGCAACTTTATTTCCATGCTCAATATGAACAATATAGTAACGATACGCGACCTGCTATTTGAACGAAGAGAAATACAAATACGTCTGGATAGTGAGCGGCGACTAGTAGATAATGAAGATGACTTCTGTGCTGCTGTAAGGGTAACTAATGTACGTGTTGTAAAAGTCATACTCAAAGTGAATCCGTATCGACCGCTTCTTCTAGTACTTAAAACGTTGAAGAGTAGGAAGGTAATCGTGAAACAAATG
..............................................................................................................................................................GACCTGCTATTTGAACGAAGA..........................................................................................................................................................................................................
.....................................................................................................................................................................................GAAATACAAATACGTCTGGAT...................................................................................................................................................................................
....................................................................................................................................................................................................................................AATGAAGATGACTTCTGTGCT....................................................................................................................................
.................................................................................................................................................................................................................................................................................................................................................................................GTGAAACAAATG
.............................................................................................................................................................................................................................................................................................................................................................................................
.............................................................................................................................................................................................................................................................................................................................................................................................
.............................................................................................................................................................................................................................................................................................................................................................................................
.............................................................................................................................................................................................................................................................................................................................................................................................
.............................................................................................................................................................................................................................................................................................................................................................................................
.............................................................................................................................................................................................................................................................................................................................................................................................
.............................................................................................................................................................................................................................................................................................................................................................................................
.............................................................................................................................................................................................................................................................................................................................................................................................



//
>PHAS61 excise_beg:3344921      excise_end:3345398      excise_len:478  Phased_ratio:0.470      Phased_num:7    phased_abun:2545
...................................................................CAATTTCCTCGATGACATGTG.....................................................................................................................................................................................................................................................................................................
..........................................................................................TAGGATTTTATATACTAGGCA..............................................................................................................................................................................................................................................................................
.........................................................................................................................................TCCATAGACGAATACCCTGCA...............................................................................................................................................................................................................................
...........................................................................................................................................................................................................................................................................................................................................................TTTGCTGTACGGTTTCTTGAT.............
.............................................................................................................................................................................................................................................................................................................................................................................................
GCAAACAACATATCATAGGACCCTATAATCATAAGCTCAAATTTATTTGGCAACTCCTTGCAATTTCCTCGATGACATGTGTAGGATTTTATATACTAGGCATTGTTAGTGATTCAAGATCATTCCATAGACGAATACCCTGCATATTGCTTGCTTTTTTATTTTGTTCCAAGAAGGCATAACAAGAATTTTAAGCCGTGGACACCTGAAAAAGAGGAGATCTAATTAGCTGTCTTGTTCATTTTCACAGTAATTTTTGTTTCTCAAAGAATAATGGAAAATCATAAGGATCTGAATATCTTTGAAACGTTGTTTGCTGTACGGTTTCTTGATTAAGTTATAA
CGTTTGTTGTATAGTATCCTGGGATATTAGTATTCGAGTTTAAATAAACCGTTGAGGAACGTTAAAGGAGCTACTGTACACATCCTAAAATATATGATCCGTAACAATCACTAAGTTCTAGTAAGGTATCTGCTTATGGGACGTATAACGAACGAAAAAATAAAACAAGGTTCTTCCGTATTGTTCTTAAAATTCGGCACCTGTGGACTTTTTCTCCTCTAGATTAATCGACAGAACAAGTAAAAGTGTCATTAAAAACAAAGAGTTTCTTATTACCTTTTAGTATTCCTAGACTTATAGAAACTTTGCAACAAACGACATGCCAAAGAACTAATTCAATATT
................................................................ACGTTAAAGGAGCTACTGTAC........................................................................................................................................................................................................................................................................................................
...........................................................................................................................................................................................................................................................ATCGACAGAACAAGTAAAAGT.............................................................................................................



//
>PHAS57 excise_beg:60064409     excise_end:60064886     excise_len:478  Phased_ratio:0.731      Phased_num:9    phased_abun:139
...................................................................CTAGGAAAGACGACACTTGCC.....................................................................................................................................................................................................................................................................................................
.................................................................................................................AGAGTGACTAAGCATTTCGAT.......................................................................................................................................................................................................................................................
......................................................................................................................................................................................................................................CCTCATGTCGAGGACTTGGCT..................................................................................................................................
.............................................................................................................................................................................................................................................................TCATTTCAGAAGAAGCTCCAG...........................................................................................................
............................................................................................................................................................................................................................................................................................................TACTTGCTTGTCTTAGATGAT............................................................
...........................................................................................................................................................................................................................................................................................................................................................AAGTGGGCTAAGTTAAGAGCA.............
.............................................................................................................................................................................................................................................................................................................................................................................................
ATAAACAATATTAACGTTGCCGAAGAACTTCCAGTCTTCCCTATAATTGGTATGGGGGGACTAGGAAAGACGACACTTGCCCAAATGATCTTCAATGATCAGAGAGTGACTAAGCATTTCGATCCCAAAATATGGGTTTGTGTCTCAGATGATTTTGATGAGAAGAGGTTAATTAAGACAATTGTAGGAAATATTGAAAGAAGTTCTCCTCATGTCGAGGACTTGGCTTCATTTCAGAAGAAGCTCCAGGAGTTATTGAATAAAAAACGATACTTGCTTGTCTTAGATGATGTTTGGAATGATGATCTAGAGAAGTGGGCTAAGTTAAGAGCAGTCTTAAATG
TATTTGTTATAATTGCAACGGCTTCTTGAAGGTCAGAAGGGATATTAACCATACCCCCCTGATCCTTTCTGCTGTGAACGGGTTTACTAGAAGTTACTAGTCTCTCACTGATTCGTAAAGCTAGGGTTTTATACCCAAACACAGAGTCTACTAAAACTACTCTTCTCCAATTAATTCTGTTAACATCCTTTATAACTTTCTTCAAGAGGAGTACAGCTCCTGAACCGAAGTAAAGTCTTCTTCGAGGTCCTCAATAACTTATTTTTTGCTATGAACGAACAGAATCTACTACAAACCTTACTACTAGATCTCTTCACCCGATTCAATTCTCGTCAGAATTTAC
.................................................................................................................................................................................................................................................................................................................................................................................GTCAGAATTTAC
.............................................................................................................................................................................................................................................................................................................................................................................................



//
>PHAS68 excise_beg:58953865     excise_end:58954258     excise_len:394  Phased_ratio:0.502      Phased_num:6    phased_abun:1047
...................................................................TACTTTACAATCATGAAAGCA.....................................................................................................................................................................................................................................................................................................
...............................................................................................................................................................................................................AAAGGAAGCATTTTAGACAAC.........................................................................................................................................................
...........................................................................................................................................................................................................................................................................................................................................................TCTTGATCGCAGTTAATCGGA.............
CTTTACCCAATGTATTGTCTGCATTTAATTAAAAACAACACACTTATTGAGGTTATTGTGTACTTTACAATCATGAAAGCAACTTGAGGCTCCAAAAGTGAACCAGTAATGTTGTCTTTGTATGCATGACAATCACCAAATTTTGTGTGCTCAGTTTCTTCAAAGAATCCACTGTCCAGTAAGTAAAAAGGAAGCATTTTAGACAACATTTTTATCTCATGAGAAATGACTTTTTTCCTTGTGCATATTGATGAGTCAAAAACTCTTATCACCCTCTTTTTCAACTCAACAACTGCCAGCACCCAATGGAATTCTTGATCGCAGTTAATCGGAATGTAGACCT
GAAATGGGTTACATAACAGACGTAAATTAATTTTTGTTGTGTGAATAACTCCAATAACACATGAAATGTTAGTACTTTCGTTGAACTCCGAGGTTTTCACTTGGTCATTACAACAGAAACATACGTACTGTTAGTGGTTTAAAACACACGAGTCAAAGAAGTTTCTTAGGTGACAGGTCATTCATTTTTCCTTCGTAAAATCTGTTGTAAAAATAGAGTACTCTTTACTGAAAAAAGGAACACGTATAACTACTCAGTTTTTGAGAATAGTGGGAGAAAAAGTTGAGTTGTTGACGGTCGTGGGTTACCTTAAGAACTAGCGTCAATTAGCCTTACATCTGGA
...............................................................................................................TTGGTCATTACAACAGAAACA.........................................................................................................................................................................................................................................................
...........................................................................................................................................................................................................................................................ACTGAAAAAAGGAACACGTAT.............................................................................................................
..........................................................................................................................................................................................................................................................................................................AGTGGGAGAAAAAGTTGAGTT..............................................................



//
>PHAS30 excise_beg:54580204     excise_end:54580912     excise_len:709  Phased_ratio:0.330      Phased_num:19   phased_abun:1254
..........................................................................................TGCAGGAAGATCATTGTAGCT..............................................................................................................................................................................................................................................................................
......................................................................................................................................................................................................................................AAGAGTCTTCAGAGCTAAGGG..................................................................................................................................
.............................................................................................................................................................................................................................................................................................................................................................................................
.............................................................................................................................................................................................................................................................................................................................................................................................
.............................................................................................................................................................................................................................................................................................................................................................................................
.............................................................................................................................................................................................................................................................................................................................................................................................
.............................................................................................................................................................................................................................................................................................................................................................................................
.............................................................................................................................................................................................................................................................................................................................................................................................
CCACAGATGAACGACTTGTTCTTTACTAAATGGATAATCTTTGGGAAAAATTGCACAGTAGGAAAAACATTGCTTTAAATGTGCAGGAAGATCATTGTAGCTCAACATCAGCGCTGGTAATATGTCATTCTGTGGCAGCTCCCATATTTCACTTTTCAAAATACGTTTCCACTCTTCAACCTCTGATTTGGAGCGTAACATGCCAGCAAGAGTCTTCAGAGCTAAGGGCAGTCCCTTGCACTTAGCTGCAATTTGTTTTCCGACCTCTTCAAGTTCTGGATGTTCCATAGGATTCATGTTTTCAAATGCATGTCTTTTAAATAAAGACCAAGAGGCTTCAATA
GGTGTCTACTTGCTGAACAAGAAATGATTTACCTATTAGAAACCCTTTTTAACGTGTCATCCTTTTTGTAACGAAATTTACACGTCCTTCTAGTAACATCGAGTTGTAGTCGCGACCATTATACAGTAAGACACCGTCGAGGGTATAAAGTGAAAAGTTTTATGCAAAGGTGAGAAGTTGGAGACTAAACCTCGCATTGTACGGTCGTTCTCAGAAGTCTCGATTCCCGTCAGGGAACGTGAATCGACGTTAAACAAAAGGCTGGAGAAGTTCAAGACCTACAAGGTATCCTAAGTACAAAAGTTTACGTACAGAAAATTTATTTCTGGTTCTCCGAAGTTAT
................................................................ATCCTTTTTGTAACGAAATTT........................................................................................................................................................................................................................................................................................................
......................................................................................................................................TACAGTAAGACACCGTCGAGG..................................................................................................................................................................................................................................
....................................................................................................................................................................................................................................CGTTCTCAGAAGTCTCGATTC....................................................................................................................................
...........................................................................................................................................................................................................................................................CCGTCAGGGAACGTGAATCGA.............................................................................................................
..........................................................................................................................................................................................................................................................................................................AGTTCAAGACCTACAAGGTAT..............................................................
.................................................................................................................................................................................................................................................................................................................................CCTAAGTACAAAAGTTTACGT.......................................
.............................................................................................................................................................................................................................................................................................................................................................................................
.............................................................................................................................................................................................................................................................................................................................................................................................
.............................................................................................................................................................................................................................................................................................................................................................................................
.............................................................................................................................................................................................................................................................................................................................................................................................
.............................................................................................................................................................................................................................................................................................................................................................................................



//
>PHAS50 excise_beg:264321       excise_end:264861       excise_len:541  Phased_ratio:0.470      Phased_num:14   phased_abun:698
.........................................................................................................................................AAGACTTGTTACTAACCATTC...............................................................................................................................................................................................................................
................................................................................................................................................................TTCGGAGTGTTAAGAAGGATA........................................................................................................................................................................................................
.....................................................................................................................................................................................................................................................................................TTGATGATGTCTGGGAAACTA...................................................................................
...........................................................................................................................................................................................................................................................................................................................................................ATGGAAGTAGACTTATCCTGA.............
.............................................................................................................................................................................................................................................................................................................................................................................................
.............................................................................................................................................................................................................................................................................................................................................................................................
CAATTGTTGGAATGGCCGGATTGGGTAAGACAACTCTCGCTAACAAGCTATTTCTTGATCAGTTTGTTGTATCTCATTTTGATGTCCGTGCACAATGTTGTGTATCTCAAGTATATACACGTAAAGACTTGTTACTAACCATTCTTCGGAGTGTTAAGAAGGATATAGTTATCAGTGATAAACTACCAGAGAATGAATTAGCAGATAAGTTGCGTAAACTTCTATTTGGTCAGAGGTACCTTATCCTCATTGATGATGTCTGGGAAACTACTGCATGTGATGAACTAATGCCATGCTTCTATGATGCCAATAATGGAAGTAGACTTATCCTGACAACTCGCCA
GTTAACAACCTTACCGGCCTAACCCATTCTGTTGAGAGCGATTGTTCGATAAAGAACTAGTCAAACAACATAGAGTAAAACTACAGGCACGTGTTACAACACATAGAGTTCATATATGTGCATTTCTGAACAATGATTGGTAAGAAGCCTCACAATTCTTCCTATATCAATAGTCACTATTTGATGGTCTCTTACTTAATCGTCTATTCAACGCATTTGAAGATAAACCAGTCTCCATGGAATAGGAGTAACTACTACAGACCCTTTGATGACGTACACTACTTGATTACGGTACGAAGATACTACGGTTATTACCTTCATCTGAATAGGACTGTTGAGCGGT
................................................................AGTCAAACAACATAGAGTAAA........................................................................................................................................................................................................................................................................................................
........................................................................................ACTACAGGCACGTGTTACAAC................................................................................................................................................................................................................................................................................
......................................................................................................................................ATTTCTGAACAATGATTGGTA..................................................................................................................................................................................................................................
............................................................................................................................................................................................................TGGTCTCTTACTTAATCGTCT............................................................................................................................................................
..........................................................................................................................................................................................................................................................................................................ATGACGTACACTACTTGATTA..............................................................
.................................................................................................................................................................................................................................................................................................................................CGGTACGAAGATACTACGGTT.......................................
.................................................................................................................................................................................................................................................................................................................................................................................CTGTTGAGCGGT
.............................................................................................................................................................................................................................................................................................................................................................................................



//
>PHAS28 excise_beg:51384504     excise_end:51385716     excise_len:1213 Phased_ratio:0.345      Phased_num:50   phased_abun:19905
...................................................................TGATGCTTTTGGAATAACGAA.....................................................................................................................................................................................................................................................................................................
..........................................................................................AGGGTTACTTCAAGAAATTGA..............................................................................................................................................................................................................................................................................
.................................................................................................................CTCATTTGACTTATATGTTGG.......................................................................................................................................................................................................................................................
.........................................................................................................................................TGACAATCTTAAAAAGCTATA...............................................................................................................................................................................................................................
.......................................................................................................................................................................................AAGGGAAGAAGTTTCTTGTTG.................................................................................................................................................................................
...............................................................................................................................................................................................................CCCTGGATGATGTGTGGAATG.........................................................................................................................................................
......................................................................................................................................................................................................................................ATAACTATAATGAGTGGGATG..................................................................................................................................
.....................................................................................................................................................................................................................................................................................AAGGAGATATAGGAAGTAAGA...................................................................................
...................................................................................................................................................................................................................................................................................................................................AGAATGTTGCCTCGATGATGG.....................................
...........................................................................................................................................................................................................................................................................................................................................................TAGCAGAGCAATAAACGTGGG.............
...................................................................................................................................................................................................................................................................................................................................................................................AACTCTGTTC
.............................................................................................................................................................................................................................................................................................................................................................................................
.............................................................................................................................................................................................................................................................................................................................................................................................
.............................................................................................................................................................................................................................................................................................................................................................................................
.............................................................................................................................................................................................................................................................................................................................................................................................
.............................................................................................................................................................................................................................................................................................................................................................................................
.............................................................................................................................................................................................................................................................................................................................................................................................
.............................................................................................................................................................................................................................................................................................................................................................................................
.............................................................................................................................................................................................................................................................................................................................................................................................
.............................................................................................................................................................................................................................................................................................................................................................................................
.............................................................................................................................................................................................................................................................................................................................................................................................
.............................................................................................................................................................................................................................................................................................................................................................................................
```

```
.......................................................................................................................................................................................................................................................................................................................................................
.......................................................................................................................................................................................................................................................................................................................................................
.......................................................................................................................................................................................................................................................................................................................................................
GTAAGACAACACTTGCTAAAGGTTTCGTTTGAAAGCTTGGTTTTGTGTTTCCAAGGCATATGATGCTTTTGGAATAACGAAAGGGTTACTTCAAGAAATTGACTCATTTGACTTATATGTTGGTGACAATCTTAAAAAGCTATAGGTCAATTGAAAGAAAGCCCGAAGGGAAGAAGTTTCTTGTTGCCCTGGATGATGTGTGGAATGATAACTATAATGAGTGGGATGACTTGAGAAATCTTTCTGTACAAGGAGATATAGGAAGTAAGATCATTGTAACTACACGTAAGGAGAATGTTGCCTCGATGATGGTAGCAGAGCAATAAACGTGGGAACTCTGTTC
CATTCTGTTGTGAACGATTTCCAAAGCAAACTTTCGAACCAAAACACAAAGGTTCCGTATACTACGAAAACCTTATTGCTTTCCCAATGAAGTTCTTTAACTGAGTAAACTGAATATACAACCACTGTTAGAATTTTTCGATATCCAGTTAACTTTCTTTCGGGCTTCCCTTCTTCAAAGAACAACGGGACCTACTACACACCTTACTATTGATATTACTCACCCTACTGAACTCTTTAGAAAGACATGTTCCTCTATATCCTTCATTCTAGTAACATTGATGTGCATTCCTCTTACAACGGAGCTACTACCATCGTCTCGTTATTTGCACCCTTGAGACAAG
...............................................................................TTTCCCAATGAAGTTCTTTAA...................................................................................................................................................................................................................................................
....................................................................................................CTGAGTAAACTGAATATACAA..............................................................................................................................................................................................................................
.........................................................................................................................CCACTGTTAGAATTTTTCGAT.........................................................................................................................................................................................................
..............................................................................................................................................ATCCAGTTAACTTTCTTTCGG....................................................................................................................................................................................
...................................................................................................................................................................GCTTCCCTTCTTCAAAGAACA...............................................................................................................................................................
........................................................................................................................................................................................ACGGGACCTACTACACACCTT..........................................................................................................................................
.............................................................................................................................................................................................................ACTATTGATATTACTCACCCT.....................................................................................................................
..................................................................................................................................................................................................................................ACTGAACTCTTTAGAAAGACA................................................................................................
.......................................................................................................................................................................................................................................................TGTTCCTCTATATCCTTCATT...........................................................................
............................................................................................................................................................................................................................................................................CTAGTAACATTGATGTGCATT......................................................
.................................................................................................................................................................................................................................................................................................CCTCTTACAACGGAGCTACTA.................................
...........................................................................................................................................................................................................................................................................................................................................CCTTGAGACAAG
.......................................................................................................................................................................................................................................................................................................................................................
.......................................................................................................................................................................................................................................................................................................................................................
.......................................................................................................................................................................................................................................................................................................................................................
.......................................................................................................................................................................................................................................................................................................................................................
.......................................................................................................................................................................................................................................................................................................................................................
.......................................................................................................................................................................................................................................................................................................................................................
.......................................................................................................................................................................................................................................................................................................................................................
.......................................................................................................................................................................................................................................................................................................................................................
.......................................................................................................................................................................................................................................................................................................................................................
.......................................................................................................................................................................................................................................................................................................................................................
.......................................................................................................................................................................................................................................................................................................................................................
.......................................................................................................................................................................................................................................................................................................................................................
.......................................................................................................................................................................................................................................................................................................................................................



//
>PHAS63 excise_beg:50481440     excise_end:50481875     excise_len:436  Phased_ratio:0.489      Phased_num:8    phased_abun:455
............................................................AAAATAGAGATGAAGAAACTG......................................................................................................................................................................................................................................................................
.................................................................................CAACTTGGAAAGATGAGAGAT.................................................................................................................................................................................................................................................
..........................................................................................................................................................................................ATGAGAATTGGAGAAGTTATT........................................................................................................................................
..............................................................................................................................................................................................................................................................................CAACAAGACCGCTACAGCCGA....................................................
TATTAATTGGGTCATGTTCTGAAGGAGCAATTTAAAATCTTGAGCAATGAGTCATACATAAAAATAGAGATGAAGAAACTGCAACTTGGAAAGATGAGAGATTTCAGAAGGAATTTTACCTGAGAAATAAGAGGCAGAAAGATCAAGATGTGTCAAGCGTGAAAACTTACTGAATTCAGGGGGGATATGAGAATTGGAGAAGTTATTCGAAGAAAGATCAAGCCTTTGGAGATGAGAGTTGGAATAGACTGCTATTGGAAGCAATGGTCCCAACAAGACCGCTACAGCCGAGGTCAAGCTCAATAACATGGCCAGTCATGTCATCACATATGACTCCATCCCA
ATAATTAACCCAGTACAAGACTTCCTCGTTAAATTTTAGAACTCGTTACTCAGTATGTATTTTTATCTCTACTTCTTTGACGTTGAACCTTTCTACTCTCTAAAGTCTTCCTTAAAATGGACTCTTTATTCTCCGTCTTTCTAGTTCTACACAGTTCGCACTTTTGAATGACTTAAGTCCCCCCTATACTCTTAACCTCTTCAATAAGCTTCTTTCTAGTTCGGAAACCTCTACTCTCAACCTTATCTGACGATAACCTTCGTTACCAGGGTTGTTCTGGCGATGTCGGCTCCAGTTCGAGTTATTGTACCGGTCAGTACAGTAGTGTATACTGAGGTAGGGT
.............................................................................................................................................................................................................AAGCTTCTTTCTAGTTCGGAA.....................................................................................................................
............................................................................................................................................................................................................................................................................GGGTTGTTCTGGCGATGTCGG......................................................
......................................................................................................................................................................................................................................................................................................................CGGTCAGTACAGTAGTGTATA............
.......................................................................................................................................................................................................................................................................................................................................................



//
>PHAS32 excise_beg:2719362      excise_end:2719587      excise_len:226  Phased_ratio:0.572      Phased_num:4    phased_abun:276
.................................................................................TTGCTTCCGGAACGAAGTGGT...........................................................................................................................
......................................................................................................TGGAGAACTGAGTATACAGCA......................................................................................................
................................................................................................................................................TCACAGAGTAGAAAAAGTCAA............................................................
AGGGACCAAAAGGATTTGAGATGCATTATATTTGTTGAGAGAATTGTTACAGCCATTGTTCTTCGGTCTCTACTGAATGAGTTGCTTCCGGAACGAAGTGGTTGGAGAACTGAGTATACAGCAGGGCATGTTTCTGTGCTCCAGTCACAGAGTAGAAAAAGTCAAAATAAAATTGTTGAAGAATTTCGCAAGGGGCTGGTAAGTCTATTTTTTTTTTATAATTAA
TCCCTGGTTTTCCTAAACTCTACGTAATATAAACAACTCTCTTAACAATGTCGGTAACAAGAAGCCAGAGATGACTTACTCAACGAAGGCCTTGCTTCACCAACCTCTTGACTCATATGTCGTCCCGTACAAAGACACGAGGTCAGTGTCTCATCTTTTTCAGTTTTATTTTAACAACTTCTTAAAGCGTTCCCCGACCATTCAGATAAAAAAAAAATATTAATT
..........................................................AAGAAGCCAGAGATGACTTAC..................................................................................................................................................



//
>PHAS86 excise_beg:58951365     excise_end:58951674     excise_len:310  Phased_ratio:0.330      Phased_num:5    phased_abun:283
............................................................TAGCTGAGATTGGAGTTAGAA....................................................................................................................................................................................................................................
......................................................................................................ATAGAAACAGAATGTCTTAGA..........................................................................................................................................................................................
..........................................................................................................................................................................................AGGATACGGCTCAACTCCATT......................................................................................................
...............................................................................................................................................................................................................TCCCGTTCGAAGGATATGGCA.................................................................................
....................................................................................................................................................................................................................................TTACAGATCAAGTTTTGCCGA............................................................
AGAATATAACTAGCAGCCAGTACTTGTTGACCGACAGTCAATTACCCACTGACATTCCAATAGCTGAGATTGGAGTTAGAACCAATTCAGTCACTCCTGCGCATAGAAACAGAATGTCTTAGAGAAATATTCAATCTCCATATTGTAATTCTTTTGGGTCAAGCAAGAAGGGAAAAGAGAAATTGAAGGATACGGCTCAACTCCATTTCCCGTTCGAAGGATATGGCATTACAGATCAAGTTTTGCCGAAACTTATTGAGGATTATATGAATTGGTTGTCAAGGGGGCTCCTAAAAAATCATAACAATA
TCTTATATTGATCGTCGGTCATGAACAACTGGCTGTCAGTTAATGGGTGACTGTAAGGTTATCGACTCTAACCTCAATCTTGGTTAAGTCAGTGAGGACGCGTATCTTTGTCTTACAGAATCTCTTTATAAGTTAGAGGTATAACATTAAGAAAACCCAGTTCGTTCTTCCCTTTTCTCTTTAACTTCCTATGCCGAGTTGAGGTAAAGGGCAAGCTTCCTATACCGTAATGTCTAGTTCAAAACGGCTTTGAATAACTCCTAATATACTTAACCAACAGTTCCCCCGAGGATTTTTTAGTATTGTTAT



//
>PHAS74 excise_beg:704592       excise_end:704880       excise_len:289  Phased_ratio:0.418      Phased_num:5    phased_abun:2440
............................................................TTGGTGGCATAATATTTTGAC...............................................................................................................................................................................................................
..........................................................................................................................................................................................GATAGAGCAAGGATTGTACCC.................................................................................
TAGATGGCATTGATCATCATTACTTAAGCTGAATATTAGTGAGCATTTTTCTTGACTCATTTGGTGGCATAATATTTTGACATGATGGTGTCAAATTTTCACGTGCAGGGTGTCATTGATTTACATGTTGTCCCGGTGATAGTAGTCGTTATGATATATGAAATTTGTTCTATACGTCAAGCTAGAGATAGAGCAAGGATTGTACCCGTTAACCACTTGATTGATTCTGATGAACTTCATCACTATGTGCCTCCATTAGAAGCATTGCCCACCAGAAGACGATTCTAT
ATCTACCGTAACTAGTAGTAATGAATTCGACTTATAATCACTCGTAAAAAGAACTGAGTAAACCACCGTATTATAAAACTGTACTACCACAGTTTAAAAGTGCACGTCCCACAGTAACTAAATGTACAACAGGGCCACTATCATCAGCAATACTATATACTTTAAACAAGATATGCAGTTCGATCTCTATCTCGTTCCTAACATGGGCAATTGGTGAACTAACTAAGACTACTTGAAGTAGTGATACACGGAGGTAATCTTCGTAACGGGTGGTCTTCTGCTAAGATA
..............................................................................................................................................ATCAGCAATACTATATACTTT.............................................................................................................................
...................................................................................................................................................................AAACAAGATATGCAGTTCGAT........................................................................................................
.............................................................................................................................................................................................................GGCAATTGGTGAACTAACTAA..............................................................



//
>PHAS14 excise_beg:12309851     excise_end:12310139     excise_len:289  Phased_ratio:0.599      Phased_num:5    phased_abun:1073
.....................................................................................................................................................................AGCCTTGGACGGAATTCACCG......................................................................................................
ACCTAGAAAATTTCTTGATTGACTCTGTCATCCGATCAGGACGCATCGTCGGCCCGCATCCGCTTCCCTCCCGACAATTTCTAGCACTCTTTGAACACTCTTTTCAAAGTCCTTTTCATCTTTTCCTAGCGGTACTTGTTTGCTATCGGTATTTCACCAGTATTTAGCCTTGGACGGAATTCACCGCCCGATTTGGGTTGCATTCCCAAACAACCCGACTCGTAGACAGCACCTCGTGGTGCGATAGGGTCCGGGAACGACGGGGATCTCACCCTCTCTGGCGCCCCC
TGGATCTTTTAAAGAACTAACTGAGACAGTAGGCTAGTCCTGCGTAGCAGCCGGGCGTAGGCGAAGGGAGGGCTGTTAAAGATCGTGAGAAACTTGTGAGAAAAGTTTCAGGAAAAGTAGAAAAGGATCGCCATGAACAAACGATAGCCATAAAGTGGTCATAAATCGGAACCTGCCTTAAGTGGCGGGCTAAACCCAACGTAAGGGTTTGTTGGGCTGAGCATCTGTCGTGGAGCACCACGCTATCCCAGGCCCTTGCTGCCCCTAGAGTGGGAGAGACCGCGGGGG
..........................................................AGGCGAAGGGAGGGCTGTTAA.................................................................................................................................................................................................................
....................................................................................................AAAAGTTTCAGGAAAAGTAGA.......................................................................................................................................................................
...................................................................................................................................................................AATCGGAACCTGCCTTAAGTG........................................................................................................
.............................................................................................................................................................................................................GGTTTGTTGGGCTGAGCATCT..............................................................



//
>PHAS82 excise_beg:2700537      excise_end:2700762      excise_len:226  Phased_ratio:0.422      Phased_num:4    phased_abun:938
............................................................TGCTCTGATGTTGCATACCGA................................................................................................................................................
...........................................................................................................................CTGGAATGCTGCTACATGGCA.................................................................................
................................................................................................................................................ACAACTAGTGGACGATCATGA............................................................
GTGTATATGAATTATCCTAATGTTGTTGATCTTGTGATTTGATAATTATAAGCAAGGAGATGCTCTGATGTTGCATACCGACTTAAAAGTGGGCAAATATTGGGGAGAAAAGGGTGTTGATTACTGGAATGCTGCTACATGGCAACAACTAGTGGACGATCATGAGGTGTGTATGAACCATGTTTTATCTTCAGTGATAGATTATAAACCGTTGCTTGAGTTGGT
CACATATACTTAATAGGATTACAACAACTAGAACACTAAACTATTAATATTCGTTCCTCTACGAGACTACAACGTATGGCTGAATTTTCACCCGTTTATAACCCCTCTTTTCCCACAACTAATGACCTTACGACGATGTACCGTTGTTGATCACCTGCTAGTACTCCACACATACTTGGTACAAAATAGAAGTCACTATCTAATATTTGGCAACGAACTCAACCA
.........................................................................................................................ATGACCTTACGACGATGTACC...................................................................................



//
>PHAS20 excise_beg:2161869      excise_end:2162199      excise_len:331  Phased_ratio:0.450      Phased_num:7    phased_abun:2026
...........................................................................................................................TCTGGATGACGTGTGGAATGA..........................................................................................................................................................................................
..........................................................................................................................................................................................AGGAGATATAGGAAGTAAGAT...........................................................................................................................
.........................................................................................................................................................................................................................................................AAATGAGCAAATTAGCATGGA............................................................
TTTCAGAATAACGAAAGGGTTACTTCAAGAAATTGGCTCAACCGACTTAAAGGTTGATGACAATCTTAACCAGCTACAAGTCAAATTGAAGGAAAGCTTAAAGGGAAAGAAATTTCTTATTGTTCTGGATGACGTGTGGAATGACAACTACAATGAGTGGGATGACTTGAGAAATATTTTTGTACAAGGAGATATAGGAAGTAAGATCATTGTGACGACACGTAAAGAGAGTGTTGCCTTGATGATGGGAAATGAGCAAATTAGCATGGACAATTTGTCTACTGAAGCCTCTTGGTCTCTATTCAAAAGAAATGCATTTGAAAACATGGA
AAAGTCTTATTGCTTTCCCAATGAAGTTCTTTAACCGAGTTGGCTGAATTTCCAACTACTGTTAGAATTGGTCGATGTTCAGTTTAACTTCCTTTCGAATTTCCCTTTCTTTAAAGAATAACAAGACCTACTGCACACCTTACTGTTGATGTTACTCACCCTACTGAACTCTTTATAAAAACATGTTCCTCTATATCCTTCATTCTAGTAACACTGCTGTGCATTTCTCTCACAACGGAACTACTACCCTTTACTCGTTTAATCGTACCTGTTAAACAGATGACTTCGGAGAACCAGAGATAAGTTTTCTTTACGTAAACTTTTGTACCT
..........................................................CTGTTAGAATTGGTCGATGTT...........................................................................................................................................................................................................................
.........................................................................................................................CAAGACCTACTGCACACCTTA............................................................................................................................................................................................
.............................................................................................................................................................................................................TAGTAACACTGCTGTGCATTT........................................................................................................
..................................................................................................................................................................................................................................CTCTCACAACGGAACTACTAC...................................................................................



//
>PHAS21 excise_beg:2340887      excise_end:2341322      excise_len:436  Phased_ratio:0.725      Phased_num:8    phased_abun:134
......................................................................................................CATAGAGAAATGTTGTTGGGC............................................................................................................................................................................................................................
.....................................................................................................................................................................AAAAATATTGAGCAATTGGCA.............................................................................................................................................................
...............................................................................................................................................................................................................AAAGGAAGGAGATATCTCATT...................................................................................................................
.........................................................................................................................................................................................................................................................ACCAATGCATGGGATGTTGTG.........................................................................
GGAATTGGCAAAACGACTCTTGCTAGAAAAGTTTATGATGATATTTACATGGAGCATCACTTCTACATTCGTGCTTGGATTACTGTATCTCAAATGCATCAACATAGAGAAATGTTGTTGGGCATTTTGCGGTGTTTTTCACTGGTTAATGACAACACTTACCAAAAAAATATTGAGCAATTGGCAGAACAAGTATACAGAAGTTTGAAAGGAAGGAGATATCTCATTGGAATGGATGACGTGTGGGACACCAATGCATGGGATGTTGTGAAAAGATCTTTTCCAGATGAGAAAAATGGAAGCCGTGTCATACTTACTAGTCGACTTGCAAATGTTGGTATCT
CCTTAACCGTTTTGCTGAGAACGATCTTTTCAAATACTACTATAAATGTACCTCGTAGTGAAGATGTAAGCACGAACCTAATGACATAGAGTTTACGTAGTTGTATCTCTTTACAACAACCCGTAAAACGCCACAAAAAGTGACCAATTACTGTTGTGAATGGTTTTTTTATAACTCGTTAACCGTCTTGTTCATATGTCTTCAAACTTTCCTTCCTCTATAGAGTAACCTTACCTACTGCACACCCTGTGGTTACGTACCCTACAACACTTTTCTAGAAAAGGTCTACTCTTTTTACCTTCGGCACAGTATGAATGATCAGCTGAACGTTTACAACCATAGA
..........................................................TGAAGATGTAAGCACGAACCT........................................................................................................................................................................................................................................................................
........................................................................................................................................................................................GTCTTGTTCATATGTCTTCAA..........................................................................................................................................
...........................................................................................................................................................................................................................................................................................................................................TACAACCATAGA
```

```
................................................................................................................................................................................................................................................................................................................................................................................................................



//
>PHAS37 excise_beg:11691253     excise_end:11691898     excise_len:646  Phased_ratio:0.849      Phased_num:13   phased_abun:8756
......................................................................TTTCGTGGAACAAGAGTGCAG.....................................................................................................................................................................................................................................................................................
..............................................................................................ATTGTTGCTCATGGGATGGAG.............................................................................................................................................................................................................................................................
.......................................................................................................................TTCTTTGTGACAGGATGACGG....................................................................................................................................................................................................................................
.................................................................................................................................................................................................................................................TCAAAAGGCTTGATTGTTTGA..........................................................................................................................................
..........................................................................................................................................................................................................................................................................TAATGATTTTACCGGATCACT.................................................................................................................
...................................................................................................................................................................................................................................................................................................................................................................................TACAGGAATAATCCCAGCAGA................
................................................................................................................................................................................................................................................................................................................................................................................................................
GTCATTATGCTTTTGAACGTTGTTTCAACATAAGAGGTAAACCGTCATATCTAAAAACTCTTTCGTGGAACAAGAGTGCAGATTGTTGCTCATGGGATGGAGTTCTTTGTGACAGGATGACGGGACAAGTGATTGAGGTTGATCTCAGTTGAAGCCAACTTCGAGGAGAGTTTCATTTCAACAGTAGCCTCTTTAAGCTTTCCAATCTCAAAAGGCTTGATTGTTTGATAATGATTTTACCGGATCACTCATTTCAGCTAAATTTAGTGAGCTTTCTAGTTTGACGCATCTTGATTATCATATTCAATTTTTACAGGAATAATCCCAGCAGAAATATCTTT
CAGTAATACGAAAACTTGCAACAAAGTTGTATTCTCCATTTGGCAGTATAGATTTTTGAGAAAGCACCTTGTTCTCACGTCTAACAACGAGTACCCTACCTCAAGAAACACTGTCCTACTGCCCTGTTCACTAACTCAACTAGAGTCAACTTCGGTTGAAGCTCCTCTCAAAGTAAAGTTGTCATCGGAGAAATTCGAAAGGTTAGAGTTTTCCGAACTAACAAACTATTACTAAAATGGCCTAGTGAGTAAAGTCGATTTAAATCACTCGAAAGATCAAACTGCGTAGAACTAAATAGTATAAGTTAAAAATGTCCTTATTAGGGTCGTCTTTATAGAGAA
..............................................................................................................................................................................................TCCTCTCAAAGTAAAGTTGTC.............................................................................................................................................................................................
......................................................................................................................................................................................................................ATCGGAGAAATTCGAAAGGTT.....................................................................................................................................................................
................................................................................................................................................................................................................................................................................................GAGTAAAGTCGATTTAAATCA...........................................................................................
........................................................................................................................................................................................................................................................................................................................CTCGAAAGATCAAACTGCGTA...................................................................
................................................................................................................................................................................................................................................................................................................................................................................................................
................................................................................................................................................................................................................................................................................................................................................................................................................



//
>PHAS65 excise_beg:3979632      excise_end:3979920      excise_len:289  Phased_ratio:0.763      Phased_num:4    phased_abun:1468
..............................................................................................TTTGAAAGGCTCAAGGCGTTA.............................................................................................................................................................................................................................
........................................................................................................................................................................TCCTTTAACAGAAGCTGGACC...................................................................................................................................................
CATCTATGGAATCTCACCAACATAGTGTTTTTGGACCTTAATAATAACCACCTTGAAGGACCAATTCCCCAGCTCCCGAGATTTGAAAGGCTCAAGGCGTTATCACTTGGAAATAACAATTTGGATGGCGGAGTTGAGTTCTTATCCTTTAACAGAAGCTGGACCCAACTTGAATGGCTAGATTTATCATCCAATTCCCTAACTGGTCCAATTCCATCCAACGTAAGTGGACTACGTAACCTACAAATACTTTGGTTGTCATCAAACTACTTGAATGGGAGTATACCT
GTAGATACCTTAGAGTGGTTGTATCACAAAAACCTGGAATTATTATTGGTGGAACTTCCTGGTTAAGGGGTCGAGGGCTCTAAACTTTCCGAGTTCCGCAATAGTGAACCTTTATTGTTAAACCTACCGCCTCAACTCAAGAATAGGAAATTGTCTTCGACCTGGGTTGAACTTACCGATCTAAATAGTAGGTTAAGGATTGACCAGGTTAAGGTAGGTTGCATTCACCTGATGCATTGGATGTTTATGAAACCAACAGTAGTTTGATGAACTTACCCTCATATGGA
....................................................................CTGGTTAAGGGGTCGAGGGCT.......................................................................................................................................................................................................................................
...............................................................................................................................................................................................................................................CAGGTTAAGGTAGGTTGCATT............................................................................



//
>PHAS81 excise_beg:4127097      excise_end:4127343      excise_len:247  Phased_ratio:0.648      Phased_num:4    phased_abun:123
ATGATTCCTAGTATCAATTATATATGGGAAAAGGGACAGATTTACCCCCGAACTTTAATAAATGGTACGTCTATGTCCTCCGTTATACTTGCGTCCACATAAGTCCCTGCCATCCAATTATAGGTACACATATACCCTCTCACTAACGGACCCCTCATTTTTTACACGTGTCTTAATCCTATTGAACTACTGTTTGACTCTTCTTTTAACCCATAAAACAACCATCCGCCCCATAACCCAATAA
TACTAAGGATCATAGTTAATATATACCCTTTTCCCTGTCTAAATGGGGGCTTGAAATTATTTACCATGCAGATACAGGAGGCAATATGAAACGCAGGTGTATTCAGGGACGGTAGGTTAATATCCATGTGTATATGGGGAGAGTGATTGCCTGGGGAGTAAAAAATGTGCACAGAATTAGGATAACTTGATGACAAACTGAGAAGAAAATTGGGTATTTTGTTGGTAGGCGGGGTATTGGGTTATT
....................................................................ATTTACCATGCAGATACAGGA......................................................................................................................................................................................................
............................................................................................GGCAATATGAAACGCAGGTGT..............................................................................................................................................................................
....................................................................................................................ATTCAGGGACGGTAGGTTAAT......................................................................................................................................................
..............................................................................................................................................................................................AATGTGCACAGAATTAGGATA............................................................................



//
>PHAS83 excise_beg:11227196     excise_end:11227400     excise_len:205  Phased_ratio:0.444      Phased_num:4    phased_abun:568
................................................................................................................................................TTGCCTCAAACTTCCGCGGCC.........................................................................
ATCCCCATCACAATAAATTTTCAAAGATTACCTGGGCCTGTGGGCTAAGGCTATAAGCTCGTTGAATACATCAGTGTAGCACGCGTGCAGCCCAGAACATCTAAGGGCATCACAGACCTGTTATTGCCTCAAACTTCCGCGGCCTAAAAAGTTGTAGTCCCTCTAAGAAGCTGTCCGCGAAGGGATACCTCCGCATAGCTAGTT
TAGGGGTAGTGTTATTTAAAAGTTTCTAATGGACCCGGACACCCGATTCCGATATTCGAGCAACTTATGTAGTCACATCGTGCGCACGTCGGGTCTTGTAGATTCCCGTAGTGTCTGGACAATAACGGAGTTTGAAGGCGCCGGATTTTTCAACATCAGGGAGATTCTTCGACGGCGCTTCCCTATGGAGGCGTATCGATCAA
....................................................................AGCAACTTATGTAGTCACATC.....................................................................................................................................................
....................................................................................................................GATTCCCGTAGTGTCTGGACA.....................................................................................................
.............................................................................................................................................ATAACGGAGTTTGAAGGCGCC............................................................................



//
>PHAS17 excise_beg:61236491     excise_end:61236821     excise_len:331  Phased_ratio:0.659      Phased_num:4    phased_abun:164
......................................................................GTTGATCAGTCACGTCGGGGA......................................................................................................................................................................................................................................................................................................
................................................................................................................................................................................................AGAAGATTTTATGGAACTTTC............................................................................................................................................................................
..................................................................................................................................................................................................................................................................................................TTACAGACGAGACAAAAAGCA..........................................................................
AGGGACAGAGTGGTTTGGAATGGGTAGCAGAATTATTTTAACATCAAGAGACAAGCACCTGTTGATCAGTCACGTCGGGGATAACGTGTATGAAGTCCAACTATTATCCGAGGATGAAGCACTTGAATTGTTCAGCAGGCATGCTTTTAGAGAAAAATCACCAAAAGAAGATTTTATGGAACTTTCGAGAGAAGTGGTGAAACATGCTGGTGGACTCTCGTTAGCTCTTAAAGTTTTGGGTTCTTCATTTTACAGACGAGACAAAAAGCACTGGAGACACATAATTGATCGGCTGAAGAGAATCCCTCACAAGGATATTCTAGGAAAGCT
TCCCTGTCTCACCAAACCTTACCATCGTCTTAATAAAATTGTAGTTCTCTGTTCGTGGACAACTAGTCAGTGCAGCCCCTATTGCACATACTTCAGGTTGATAATAGGCTCCTACTTCGTGAACTTAACAAGTCGTCCGTACGAAAATCTCTTTTTAGTGGTTTCTTCTAAAATACCTTGAAAGCTCTCTCACCACTTTGTACGACCACCTGAGAGCAATCGAGAATTTCAAAACCCAAGAAGTAAAATGTCTGCTCTGTTTTTCGTGACCTCTGTGTATTAACTAGCCGACTTCTCTTAGGGAGTGTTCCTATAAGATCCTTTCGA
............................................................................................CTATTGCACATACTTCAGGTT................................................................................................................................................................................................................................................................................



//
>PHAS03 excise_beg:28004455     excise_end:28004806     excise_len:352  Phased_ratio:0.932      Phased_num:6    phased_abun:307
......................................................................ATTGGCAAAACTACGATAGCT.....................................................................................................................................................................................................................................................................................
.......................................................................................................................TTTCATAGATTTGAAGGCAGT....................................................................................................................................................................................................................................
........................................................................................................................................................................AAGTTTTAGAAAAGTATGAC...................................................................................................................................................................................................
...........................................................................................................................................................................................................................................................................................................................AGCTGCAAAAGAGTTCTTCTT................................................................
TTGTGGTTAAAAGAGGGATACAATTATGTCGATATTCTTGCTATACATGGTATGGGAGGTATTGGCAAAACTACGATAGCTAAAACAGCTTATAACCTGAATTTTCATAGATTTGAAGGCAGTAGCTTCCTTGCTGATGTGGGAAAAGTTTTAGAAAAGTATGACGGTTTAGGTCGTTTACAAAGACAACTTCTTTCCAATATTCTTGGGAAGGATGTCGAAAAGATATAATGTTAATGAAGGGTCCGTCAAGATTCAAGAAGCTATCAGCTGCAAAAGAGTTCTTCTTGTTCTTGATGATATAGACAACATAGATCAGCTAAATGCGGTACTCGGTATGA
AACACCAATTTTCTCCCTATGTTAATACAGCTATAAGAACGATATGTACCATACCCTCCATAACCGTTTTGATGCTATCGATTTTGTCGAATATTGGACTTAAAAGTATCTAAACTTCCGTCATCGAAGGAACGACTACACCCTTTTCAAAATCTTTTCATACTGCCAAATCCAGCAAATGTTTCTGTTGAAGAAAGGTTATAAGAACCCTTCCTACAGCTTTTCTATATATTACAATTACTTCCCAGGCAGTTCTAAGTTCTTCGATAGTCGACGTTTTCTCAAGAAGAACAAGAACTACTATATCTGTTGTATCTAGTCGATTTACGCCATGAGCCATACT
................................................................................................................................................................................................................................................................................................GGCAGTTCTAAGTTCTTCGAT...........................................................................................
........................................................................................................................................................................................................................................................................................................................AGTCGACGTTTTCTCAAGAAG...................................................................



//
>PHAS70 excise_beg:88582539     excise_end:88582806     excise_len:268  Phased_ratio:0.551      Phased_num:5    phased_abun:486
......................................................................TGAAATTTGCTCTTGATCTGC............................................................................................................................................................................................................................
..............................................................................................TCATAAAAAGCAGTCGTGTCG....................................................................................................................................................................................................
.........................................................................................................................................................................................................................TCCGTCAAAGCCTCTGGAAA..........................................................................
CTGTACTTCTTCTTCAGTCGGCGGAGTTTCTCCACAAGCTGGTTTTTGTTAAAATCCAGCTGAAATTTGCTCTTGATCTGCTCATAAAAAGCAGTCGTGTCGTGTAATGGTGCTGCGGGGAAGACGTGTTCAAGCCCCGCTGGGTGGTGTAGTCCAAAAATCCACGCAACAGCTCAATCTCGTCTTCATCCGTCCAAAGCCTCTGGAAAAGCTTCCGAGACTCGTCAAAACCAGCCGATGGCTTCTTTACCGCTCCGGGCAGAATGACC
GACATGAAGAAGAAGTCAGCCGCCTCAAAGAGGTGTTCGACCAAAAACAATTTTAGGTCGACTTTAAACGAGAACTAGACGAGTATTTTTCGTCAGCACAGCATTACCACGACGCCCCTTCTGCACAAGTTCGGGGCGACCCACCACATCAGGTTTTTAGGTGCGTTGTCGAGTTAGAGCAGAAGTAGGCAGGTTTCGGAGACCTTTTCGAAGGCTCTGAGCAGTTTTGGTCGGCTACCGAAGAAATGGCGAGGCCCGTCTTACTGG
..............................................................................................................................................................................................CGTTGTCGAGTTAGAGCAGAA....................................................................................................
......................................................................................................................................................................................................................GTAGGCAGGTTTCGGAGACCT............................................................................



//
>PHAS45 excise_beg:28907395     excise_end:28908334     excise_len:940  Phased_ratio:0.542      Phased_num:17   phased_abun:1070
................................................................................................................................................................................................AGAGTGTTGAAGATGGAATGA...........................................................................................................................................................................................
..........................................................................................................................................................................................................................................................................TGCTGGATGATGTTGATGAAG.................................................................................................................
...........................................................................................................................................................................................................................................................................................................................TTGGAATGCGAGATTGGTTTT................................................................
...................................................................................................................................................................................................................................................................................................................................................ATTCAGGCAGTAAAATTATAG........................................
................................................................................................................................................................................................................................................................................................................................................................................................................
................................................................................................................................................................................................................................................................................................................................................................................................................
................................................................................................................................................................................................................................................................................................................................................................................................................
................................................................................................................................................................................................................................................................................................................................................................................................................
................................................................................................................................................................................................................................................................................................................................................................................................................
................................................................................................................................................................................................................................................................................................................................................................................................................
................................................................................................................................................................................................................................................................................................................................................................................................................
................................................................................................................................................................................................................................................................................................................................................................................................................
................................................................................................................................................................................................................................................................................................................................................................................................................
GGAAGACAACAATCGCCAAATATGCATTTAACTCTAACTTTGAAAGTTTTGAAAGAAGCAGCTTCCTCTACAATGTTAGAGATTTTTCAGAGTCTACGGATGGACTAATTTATCTGCAGAAACAATTTCTTTGTGATATTCTTGACGGGAGGAAGATTGACATTCAGAGTGTTGAAGATGGAATGAATCAGATCAAAAGTGCTGTTTATGGTAAGAGGGTTCTCATTGTGCTGGATGATGTTGATGAAGTGGAACAATTAGCTGCTATCGTTGGAATGCGAGATTGGTTTTTATTCAGGCAGTAAAATTATAGTGACAACAAGGCACATAGAGTTACTAAGGGC
CCTTCTGTTGTTAGCGGTTTATACGTAAATTGAGATTGAAACTTTCAAAACTTTCTTCGTCGAAGGAGATGTTACAATCTCTAAAAAGTCTCAGATGCCTACCTGATTAAATAGACGTCTTTGTTAAAGAAACACTATAAGAACTGCCCTCCTTCTAACTGTAAGTCTCACAACTTCTACCTTACTTAGTCTAGTTTTCACGACAAATACCATTCTCCCAAGAGTAACACGACCTACTACAACTACTTCACCTTGTTAATCGACGATAGCAACCTTACGCTCTAACCAAAATAAGTCCGTCATTTTAATATCACTGTTGTTCCGTGTATCTCAATGATTCCCG
....................................................................GTCGAAGGAGATGTTACAATC.......................................................................................................................................................................................................................................................................................................
....................................................................................................................ACCTGATTAAATAGACGTCTT.......................................................................................................................................................................................................................................................................
.....................................................................................................................................................................ACTGCCCTCCTTCTAACTGTA......................................................................................................................................................................................................
..............................................................................................................................................................................................AGTCTCACAACTTCTACCTTA.............................................................................................................................................................................................



//
>PHAS35 excise_beg:65690087     excise_end:65690417     excise_len:331  Phased_ratio:0.464      Phased_num:4    phased_abun:125
..........................................................................................................................................................................................................................................................................TTGGACTTCATACACGTTATC..................................................................................................
CTTTCCTAGAATATCCTTGTGAGGGATTCTCTTCAGCCGATCAATTATGTGTCTCCAGTGCTTTTTGTCTCGTCTGTAAAATGAAGAACCCAAAAGTTTAAGAGCTAACGGGAGTCCACCAGCATGTTTCACCACTTCTCTCGAAAGTTCAATAAAATCTTCTTTTGGTGTTTTTTCTCTAAAAGCATGCCTACTGAACAATTCAAGTGCTTCAACCTCGGATAATAGTTGGACTTCATACACGTTATCCCCGACGTGACTGATCAACAGGTTCTTGTCTCTTGATTTAAAATAATTCTGCTACCCATTCCAAACCACTCTGTCCCTCCA
GAAAGGATCTTATAGGAACACTCCCTAAGAGAAGTCGGCTAGTTAATACACAGAGGTCACGAAAAACAGAGCAGACATTTTACTTCTTGGGTTTTCAAATTCTCGATTGCCCTCAGGTGGTCGTACAAAGTGGTGAAGAGAGCTTTCAAGTTATTTTAGAAGAAAACCACAAAAAAGAGATTTTCGTACGGATGACTTGTTAAGTTCACGAAGTTGGAGCCTATTATCAACCTGAAGTATGTGCAATAGGGGCTGCACTGACTAGTTGTCCAAGAACAGAGAACTAAATTTTATTAAGACGATGGGTAAGGTTTGGTGAGACAGGGAGGT
....................................................................ACGAAAAACAGAGCAGACATT................................................................................................................................................................................................................................................................................................
.....................................................................................................................................................................CTTTCAAGTTATTTTAGAAGA.......................................................................................................................................................................................
................................................................................................................................................................................................................................................................................................AGGGGCTGCACTGACTAGTTG............................................................................



//
>PHAS91 excise_beg:61676305     excise_end:61676559     excise_len:255  Phased_ratio:0.310      Phased_num:4    phased_abun:171
...............................................................................TGAAGGAAAATGGTAAGTCTG....................................................................................................................................................................................................
```

```
...TCAAGCTAGTGAGACAAGCCG...
...TGATAACTTGTAAGAAATCAT...
TATGGATGGTGACCATCTTCATGTGAGGTGTATGGCACATATCCTTAATCTTATTGTGCAAGATGGGTTGAAGGAAAATGGTAAGTCTGTCAAGCTAGTGAGACAAGCCGTGAAATACATTAAACAATCTCCCGCAAAACTTAGAAAGTTTAAAGAATGTTGTGAATCTGAACTGATAACTTGTAAGAAATCATTATGTTTAGATGTTCCTACTAGGTGGAACTCTACATATTCGATGCTTGATATAACACAAC
ATACCTACCACTGGTAGAAGTACACTCCACATACCGTGTATAGGAATTAGAATAACACGTTCTACCCAACTTCCTTTTACCATTCAGACAGTTCGATCACTCTGTTCGGCACTTTATGTAATTTGTTAGAGGGCGTTTTGAATCTTTCAAATTTCTTACAACACTTAGACTTGACTATTGAACATTCTTAGTAATACAAATCTACAAGGATGATCCACCTTGAGATGTATAAGCTACGAACTATATTGTGTTG
...CTCCACATACCGTGTATAGGA...


//
>PHAS49 excise_beg:1242990	excise_end:1243341	excise_len:352	Phased_ratio:0.694	Phased_num:8	phased_abun:6532
...TGAACTAATGGAGATTGTGTA...
...TAGAGGTCTGAAGGGTAGGAG...
...TATTTGGAGTACTGAGGCATG...
...AAGTCTAGAAGATAGTTGGAA...
AATGCTTCTTGAAGTTGTCTCTTCAATTACTACCAAAGGCAGTCAAGAAATGAGCAACGATGAACTAATGGAGATTGTGTATAGAGGTCTGAAGGGTAGGAGATTCCTAATTGTCATAGATGATATTTGGAGTACTGAGGCATGGAACCAAATGCAAAGAATATTTCCAAACGATGACAATAAAAGCCGAATTCTATTAACTACTCGGCTCAAGTATGTTGCTGATTATGTCAACTGCCCTGATTTTCCACCTCATAGTAAGTCTTTTCTAAGTCTAGAAGATAGTTGGAATCTATTCACCGAAAAATTATTCAAGAAAGACCAGTGTCCTCCTTTACTTGTA
TTACGAAGAACTTCAACAGAGAAGTTAATGATGGTTTCCGTCAGTTCTTTACTCGTTGCTACTTGATTACCTCTAACACATATCTCCAGACTTCCCATCCTCTAAGGATTAACAGTATCTACTATAAACCTCATGACTCCGTACCTTGGTTTACGTTTCTTATAAAGGTTTGCTACTGTTATTTTCGGCTTAAGATAATTGATGAGCCGAGTTCATACAACGACTAATACAGTTGACGGGACTAAAAGGTGGAGTATCATTCAGAAAAGATTCAGATCTTCTATCAACCTTAGATAAGTGGCTTTTAATAAGTTCTTTCTGGTCACAGGAGGAAATGAACAT
...AAAGGTTTGCTACTGTTATTT...
...TCGGCTTAAGATAATTGATGA...
...GCCGAGTTCATACAACGACTA...
...ATACAGTTGACGGGACTAAAA...


//
>PHAS71 excise_beg:421966	excise_end:422170	excise_len:205	Phased_ratio:0.653	Phased_num:5	phased_abun:179
...ATGTCATTGATACGAGCGTGT...
...TTTTCAAGCAACATCTAGAGG...
...TGTCTGGTTGGCCTCTTCTAG...
ACCAAATTGCTAAAATATTTGGTTATAGTCCCTTTTGTCAAAGTTGTTTTTCCTATTCCACCAATGCCATATATACCAACCATGTCATTGATACGAGCGTGTTTTCAAGCAACATCTAGAGGTGTCTGGTTGGCCTCTTCTAGGACTTGTTTTATGATATTCTCCATAAATTTAAATTCACACCTACAAATTGTTAGGGAAAA
TGGTTTAACGATTTTATAAACCAATATCAGGGAAAACAGTTTCAACAAAAAGGATAAGGTGGTTACGGTATATATGGTTGGTACAGTAACTATGCTCGCACAAAAAGTTCGTTGTAGATCTCCACAGACCAACCGGAGAAGATCCTGAACAAAATACTATAAGAGGTATTTAAATTTAAGTGTGGATGTTTAACAATCCCTTTT
...GTGGTTACGGTATATATGGTT...
...GGTACAGTAACTATGCTCGCA...


//
>PHAS80 excise_beg:59189313	excise_end:59189664	excise_len:352	Phased_ratio:0.320	Phased_num:7	phased_abun:401
...TTGAGAATGATTGCTTACCAG...
...AAACGACAGATTACACATAGC...
...CAGTACTACGAGAGAAGATCG...
...TGAAAAAGGAACGATGAAAAC...
...AACTGGTGAGAAGGGATGCAG...
AAGTAATCATCGTTATTTGTGTTAAATTAAATGTCATTGACCTGCATCTAGTCTTTCCACTTGAGAATGATTGCTTACCAGCAAAGCAGCATTTGTTGGTTGAAACGACAGATTACACATAGCTCCAACGACTAACCTAGAACTTGAACTAACCGACCCTTCCCGCAGTACTACGAGAGAAGATCGTGAAAAAGGAACGATGAAAACAATGTCTTCATTCAGGCCAATATTCCTCTACCTGATCCATTCACTCTCATCTGCAGCAATCACAACTGGTGAGAAGGGATGCAGCGAAGCAGTTCTCGTACCACTCTCAAACTTTGTGTCCCAAGTGGCAATTTGA
TTCATTAGTAGCAATAAACACAATTTAATTTACAGTAACTGGACGTAGATCAGAAAGGTGAACTCTTACTAACGAATGGTCGTTTCGTCGTAAACAACCAACTTTGCTGTCTAATGTGTATCGAGGTTGCTGATTGGATCTTGAACTTGATTGGCTGGGAAGGGCGTCATGATGCTCTCTTCTAGCACTTTTTCCTTGCTACTTTTGTTACAGAAGTAAGTCCGGTTATAAGGAGATGGACTAGGTAAGTGAGAGTAGACGTCGTTAGTGTTGACCACTCTTCCCTACGTCGCTTCGTCAAGAGCATGGTGAGAGTTTGAAACACAGGGTTCACCGTTAAACT
...CGAGGTTGCTGATTGGATCTT...
...TGTTACAGAAGTAAGTCCGGT...


//
>PHAS06 excise_beg:33140166	excise_end:33140412	excise_len:247	Phased_ratio:0.561	Phased_num:5	phased_abun:1610
...TTCAATGGACTTCTCGCGACG...
...TCACCGGATCATTCAATCGGT...
GAGTTCGCGGGTCGTTCTGTTGTGCAGGTTTCGACAATGATCCTTCCGCAGGTTCACCTACGGAAACCTTGTTACGACTTCTCCTTCCTCTAAATGATAAGGTTCAATGGACTTCTCGCGACGTTGCGGGCAGCGAACCTCCCACGTCGCTGTGATTCAAACATTTCACCGGATCATTCAATCGGTAGGAGCGACGGGCGGTGTGTGCAAAGGGCAGGGACGTAGTCAACGTGAGCTGATGACTCG
CTCAAGCGCCCAGCAAGACAACACGTCCAAAGCTGTTACTAGGAAGGCGTCCAAGTGGATGCCTTTGGAACAATGCTGAAGAGGAAGGAGATTTACTATTCCAAGTTACCTGAAGAGCGCTGCAACGCCCGTCGCTTGGAGGGTGCAGCGACACTAAGTTTGTAAAGTGGCCTAGTAAGTTAGCCATCCTCGCTGCCCGCCACACACGTTTCCGTCCTGCATCAGTTGCACTCGACTACTGAGC
...ATGCCTTTGGAACAATGCTGA...
...CCAAGTTACCTGAAGAGCGCT...
...AAAGTGGCCTAGTAAGTTAGC...


//
>PHAS56 excise_beg:70361711	excise_end:70362020	excise_len:310	Phased_ratio:0.785	Phased_num:5	phased_abun:4601
...TTGGGCTGTACCAGATGGTTG...
...GGCTTGGTGGGCTAATTTCTG...
...TGAAGAAATGGACTACTTGAT...
...TCGTAAAGTGGGGCGGAATGC...
...GCGGTGGAAGGATAACGAGTC...
TATGCCGAGCTTAGTAATGTGGGCTTGTAACCCAAATGGGGGCATTAATGTGGTGGAATGTTGGGCTGTACCAGATGGTTGGGCTTGGTGGGCTAATTTCTGACTTGCCCTATCCAAGCTTAGAGTTGGATCATGTGGCCCAATTGAAGAAATGGACTACTTGATTCGTAAAGTGGGGCGGAATGCGTGAGGCTGGTTTCACAGAGCAACGATAAGTTTCCGCTCTAAGCGGTGGAAGGATAACGAGTCGGTGTTCTCTGAGTCCAGTAACGCCTGATGGGTTGCTCCAATTAAACCACCATTTTTCTT
ATACGGCTCGAATCATTACACCCGAACATTGGGTTTACCCCCGTAATTACACCACCTTACAACCCGACATGGTCTACCAACCCGAACCACCCGATTAAAGACTGAACGGGATAGGTTCGAATCTCAACCTAGTACACCGGGTTAACTTCTTTACCTGATGAACTAAGCATTTCACCCCGCCTTACGCACTCCGACCAAAGTGTCTCGTTGCTATTCAAAGGCGAGATTCGCCACCTTCCTATTGCTCAGCCACAAGAGACTCAGGTCATTGCGGACTACCCAACGAGGTTAATTTGGTGGTAAAAAGAA


//
>PHAS51 excise_beg:65142532	excise_end:65142904	excise_len:373	Phased_ratio:0.751	Phased_num:8	phased_abun:778
...ATAGACAAGGAAGGAGAACGA...
...TGAAGAAGTCATGACGTAAGA...
...AAGACGTAAGAGATAACACGG...
...ATGGGGTAGGTAGAGGAACGA...
...AAGCGGTGAGAAAAGCTCAG...
AATTCAAATCTTTTTGTTGTGTTCAATTGTACATGGAATAAAATCAACAGCTCAGGAGGGATAGACAAGGAAGGAGAACGAGAGTTGAGTAGGAAAGAAGAAGGAGACCAAACAAAGCTTACATGAAGAAGTCATGACGTAAGATTTGCAAAAAAACAAAACATAAAGACGTAAGAGATAACACGGGGTCTTACAAGGTCAAGAAAAGGTCTTACAAGGTCAAGAAGAATGGGGTAGGTAGAGGAACGAAAGGCAACAAGTCAAAAAAGAGAATACACAACAGAAAAAAAAAAGCGGTGAGAAAAGCTCAGGTAGGATAGCACCGAGATTAAGATGAGGATG
TTAAGTTTAGAAAAACAACACAGTTAACATGTACCTTATTTTAGTTGTCGAGTCCTCCCTATCTGTTCCTTCCTCTCTTGCTCTCAACTCATCCTTTCTTCTTCCTCTGGTTTGTTTCGAATGTACTTCTTCAGTACTGCATTCTAAACGTTTTTTTGTTTTGTATTTCTGCATTCTCTATTGTGCCCCAGAATGTTCCAGTTCTTTTCCAGAATGTTCCAGTTCTTCTACCCCATCCATCTCCTTGCTTCCGTTGTTCAGTTTTTTCTCTTATGTGTTGTCTTTTTTTTTTCGCCACTCTTTTCGAGTCCATCCTATCGTGGCTCTAATTCTACTCCTAC
...CCCCAGAATGTTCCAGTTCTT...
...TTCCAGAATGTTCCAGTTCTT...
...CTTTCCGTTGTTCAGTTTTTT...


//
>PHAS25 excise_beg:18662824	excise_end:18663154	excise_len:331	Phased_ratio:0.687	Phased_num:4	phased_abun:464
...ACTAATTAGATGACGAGGCAT...
...CCAGTTCTGAGTTGGCTGTTC...
TGGTTTCGCTGGATAGTAGAAAGGCACACACGAAATCTCGTTAATCCATTCATGCTCGTCACTAATTAGATGACGAGGCATTTGACTACCATAAGAGAGTCATAGTTACTCCAGCCATTTACCCGCGCTTGGTTGAATGTCTTCACTTTGACATTCAGAGCACTGGGCAGAAATCACATTGCGTAAACATCCGTTGGGACCATCGCAATGCTTTGTTTTTAATTAAATAGTCAGATTCCCCTTGTTCGTACCAGTTCTGAGTTGGCTGTTCGACGTCCTGGGAAGTCCCCCGAAGGAACCGTTCCTTGTCCGTCCCAATCCGGCACGCGG
ACCAAAGCGACCTATCATCTTTCCGTGTGTGCTTTAGAGCAATTAGGTAAGTACGAGCAGTGATTAATCTACTGCTCCGTAAACTGATGGTATTCTCTCAGTATCAATGAGGTCGGTAAATGGGCGCGAACCAACTTACAGAAGTGAAACTGTAAGTCTCGTGACCCGTCTTTAGTGTAACGCATTTGTAGGCAACCCTGGTAGCGTTACGAAACAAAATTAATTTATCAGTCTAAGGGGAACAAGCATGGTCAAGACTCAACCGACAAGCTGCAGGACCCTTCAGGGGGCTTCCTTGGCAAGGAACAGGCAGGGGTTAGGCCGTGCGCC
...ACCCGTCTTTAGTGTAACGCA...
...ATGGTCAAGACTCAACCGACA...


//
>PHAS33 excise_beg:7149517	excise_end:7150225	excise_len:709	Phased_ratio:0.532	Phased_num:24	phased_abun:4421
...CTTTGCAAGGAATGCTGGTTG...
...GTTTGGTAATGGCAGTAGAAT...
...TACTGCAACAACTAGAGACAA...
...AGTGTAGTCATGGATTATTTG...
...ATAAGAGTTCAAATGTGAGAT...
...CTTGATAGTTGAATAGGAGAA...
[illegible]
...GCTCCAATCAGAGCGGAACGT...GGTTGGTTTG
[illegible]
AATGATATTCGAATTGTTGGTATATGGGGTATGGAAGGAGTAAGAAAGATAATAATTTTTGTTTTCTTGCTGATATTAAAGAATGCAAAAATAAAATGCGTTCTTTGCAAGGAATGCTGGTTGGTTTGGTAATGGCAGTAGAATTACTGCAACAACTAGAGACAAACATTTGATAGAGAAAAATGTCGTAGTATAAAATTCACTTTGAGTGTAGTCATGGATTATTTGATAAGAGTTCAAATGTGAGATCTTGATAGTTGAATAGGAGAAGCTCCAATCAGAGCGGAACGTAGGTTTGTTTCTTGGAGCATTTGATATCCTCTCATGGTTGACCGGTTGGTTTG
TTACTATAAGCTTAACAACCATATACCCCATACCTTCCTCATTCTTTCTATTATTAAAAACAAAAGAACGACTATAATTTCTTACGTTTTTATTTTACGCAAGAAACGTTCCTTACGACCAACCAAACCATTACCGTCATCTTAATGACGTTGTTGATCTCTGTTTGTAAACTATCTCTTTTTACAGCATCATATTTTAAGTGAAACTCACATCAGTACCTAATAAACTATTCTCAAGTTTACACTCTAGAACTATCAACTTATCCTCTTCGAGGTTAGTCTCGCCTTGCATCCAAACAAAGAACCTCGTAAACATAGGAGAGTACCAACTGGCCAACCAAAC
...AACAAAAGAACGACTATAATT...
...AAGAAACGTTCCTTACGACCA...
...TTTGTAAACTATCTCTTTTTA...
...CAGCATCATATTTTAAGTGAA...
...ACTCACATCAGTACCTAATAA...
...AAACATAGGAGAGTACCAACT...
...
...
...
...
...


//
```

```
>PHAS40 excise_beg:3821576      excise_end:3822410      excise_len:835  Phased_ratio:0.830      Phased_num:31   phased_abun:209390
............................................................TTATGGAATACGAGTAGATAT......................................................................................................................................................................................................................................................................
.................................................................................TGATGTACATGGGATGGATTA.................................................................................................................................................................................................................................................
......................................................................................................CAACTTGATTAGAAAACATTG............................................................................................................................................................................................................................
................................................................................................................................................CCAAAAACGGTCTTATGGAAT..................................................................................................................................................................................
.....................................................................................................................................................................GCGAGTAGAGATTGTTGTACT.............................................................................................................................................................
....................................................................................................................................................................................................................................TATGTCTTCCTCCTTAAGGTA..............................................................................................
.............................................................................................................................................................................................................................................................................................................................................CGACCATATG
.......................................................................................................................................................................................................................................................................................................................................................
.......................................................................................................................................................................................................................................................................................................................................................
.......................................................................................................................................................................................................................................................................................................................................................
.......................................................................................................................................................................................................................................................................................................................................................
.......................................................................................................................................................................................................................................................................................................................................................
.......................................................................................................................................................................................................................................................................................................................................................
.......................................................................................................................................................................................................................................................................................................................................................
AGATGGAAAATGAAAAATCTCCAATTACTATCCTGTGATACTTTTTCCCCAAAAACAGTCTTATGGAATACGAGTAGATATTGATGTACATGGGATGGATTACAACTTGATTAGAAAACATTGGACACAATACTTCAAAACTTCCCAAAAACGGTCTTATGGAATGCGAGTAGAGATTGTTGTACTTTCTCTCCCTGATGTCAACATCTCATCTTCTATACCTATGAATATGTCTTCCTCCTTAAGGTATGTGGATCTTGAATATACTAATATACAAATTAGGTATTTCAATTTCACCGACCATATATATATCTTTCACAATTTCAATTTCAGCGACCATATG
TCTACCTTTTACTTTTTAGAGGTTAATGATAGGACACTATGAAAAAGGGGTTTTTGTCAGAATACCTTATGCTCATCTATAACTACATGTACCCTACCTAATGTTGAACTAATCTTTTGTAACCTGTGTTATGAAGTTTTGAAGGGTTTTTGCCAGAATACCTTACGCTCATCTCTAACAACATGAAAGAGAGGGACTACAGTTGTAGAGTAGAAGATATGGATACTTATACAGAAGGAGGAATTCCATACACCTAGAACTTATATGATTATATGTTTAATCCATAAAGTTAAAGTGGCTGGTATATATATAGAAAGTGTTAAAGTTAAAGTCGCTGGTATAC
...............................................................................TAACTACATGTACCCTACCTA...................................................................................................................................................................................................................................................
....................................................................................................ATGTTGAACTAATCTTTTGTA..............................................................................................................................................................................................................................
.........................................................................................................................ACCTGTGTTATGAAGTTTTGA.........................................................................................................................................................................................................
..............................................................................................................................................AGGGTTTTTGCCAGAATACCT....................................................................................................................................................................................
...................................................................................................................................................................TACGCTCATCTCTAACAACAT...............................................................................................................................................................
.............................................................................................................................................................................................................TAGAGTAGAAGATATGGATAC.....................................................................................................................
...........................................................................................................................................................................................................................................................................................................................................TCGCTGGTATAC
.......................................................................................................................................................................................................................................................................................................................................................
.......................................................................................................................................................................................................................................................................................................................................................
.......................................................................................................................................................................................................................................................................................................................................................
.......................................................................................................................................................................................................................................................................................................................................................
.......................................................................................................................................................................................................................................................................................................................................................
.......................................................................................................................................................................................................................................................................................................................................................
.......................................................................................................................................................................................................................................................................................................................................................
.......................................................................................................................................................................................................................................................................................................................................................
.......................................................................................................................................................................................................................................................................................................................................................
.......................................................................................................................................................................................................................................................................................................................................................



//
>PHAS39 excise_beg:61096825     excise_end:61098289     excise_len:1465 Phased_ratio:0.689      Phased_num:51   phased_abun:122160
............................................................ATGATGTTGTTGTGTTAACAG......................................................................................................................................................................................................................................................................
.................................................................................TTACGTACGAAAATAAGTTTG.................................................................................................................................................................................................................................................
................................................................................................................................................TTTTTTTCCTCGCATAGCAAA..................................................................................................................................................................................
.....................................................................................................................................................................ACATATTTCAGAGTAATGCAA.............................................................................................................................................................
..........................................................................................................................................................................................TGGTTTGGTCGATTGCACGAG........................................................................................................................................
....................................................................................................................................................................................................................................GATAAGCGGGAAGATATGATT..............................................................................................
.........................................................................................................................................................................................................................................................TTTGGAAGGTGGAGAAGCCAG.........................................................................
........................................................................................................................................................................................................................................................................................................................TACTTGACAAGGTGGGTAATC..........
.............................................................................................................................................................................................................................................................................................................................................TGGTAGAAAT
.......................................................................................................................................................................................................................................................................................................................................................
.......................................................................................................................................................................................................................................................................................................................................................
.......................................................................................................................................................................................................................................................................................................................................................
.......................................................................................................................................................................................................................................................................................................................................................
.......................................................................................................................................................................................................................................................................................................................................................
.......................................................................................................................................................................................................................................................................................................................................................
.......................................................................................................................................................................................................................................................................................................................................................
.......................................................................................................................................................................................................................................................................................................................................................
.......................................................................................................................................................................................................................................................................................................................................................
.......................................................................................................................................................................................................................................................................................................................................................
.......................................................................................................................................................................................................................................................................................................................................................
.......................................................................................................................................................................................................................................................................................................................................................
.......................................................................................................................................................................................................................................................................................................................................................
.......................................................................................................................................................................................................................................................................................................................................................
.......................................................................................................................................................................................................................................................................................................................................................
.......................................................................................................................................................................................................................................................................................................................................................
.......................................................................................................................................................................................................................................................................................................................................................
.......................................................................................................................................................................................................................................................................................................................................................
.......................................................................................................................................................................................................................................................................................................................................................
.......................................................................................................................................................................................................................................................................................................................................................
.......................................................................................................................................................................................................................................................................................................................................................
.......................................................................................................................................................................................................................................................................................................................................................
AGTACTATCGAGATATGTGGTATGGAGGGAGTAGGAAAGAAAACCGAGGAAGGTTGTTCGATGATGTTGTTGTGTTAACAGTTACGTACGAAAATAAGTTTGGATTCTTTTTGGACAAAAAACTGATAATTCGATTTATCATCATTTTTTTCCTCGCATAGCAAAACATATTTCAGAGTAATGCAATGGTTTGGTCGATTGCACGAGCACTGAATTCATCGCAAATTTGATAAGCGGGAAGATATGATTTTTGGAAGGTGGAGAAGCCAGGTACCTATTTTTGCTTTGTTCCTTGTTTGAGGAAAATAATAATACTTGACAAGGTGGGTAATCTGGTAGAAAT
TCATGATAGCTCTATACACCATACCTCCCTCATCCTTTCTTTTGGCTCCTTCCAACAAGCTACTACAACAACACAATTGTCAATGCATGCTTTTATTCAAACCTAAGAAAAACCTGTTTTTTGACTATTAAGCTAAATAGTAGTAAAAAAAGGAGCGTATCGTTTTGTATAAAGTCTCATTACGTTACCAAACCAGCTAACGTGCTCGTGACTTAAGTAGCGTTTAAACTATTCGCCCTTCTATACTAAAAACCTTCCACCTCTTCGGTCCATGGATAAAAACGAAACAAGGAACAAACTCCTTTTATTATTATGAACTGTTCCACCCATTAGACCATCTTTA
...............................................................................TCAATGCATGCTTTTATTCAA...................................................................................................................................................................................................................................................
....................................................................................................ACCTAAGAAAAACCTGTTTTT..............................................................................................................................................................................................................................
.............................................................................................................................................................................................................TCGTGACTTAAGTAGCGTTTA.....................................................................................................................
..................................................................................................................................................................................................................................AACTATTCGCCCTTCTATACT................................................................................................
............................................................................................................................................................................................................................................................................TCCATGGATAAAAACGAAACA......................................................
......................................................................................................................................................................................................................................................................................................................TTATGAACTGTTCCACCCATT............
.......................................................................................................................................................................................................................................................................................................................................................
.......................................................................................................................................................................................................................................................................................................................................................
.......................................................................................................................................................................................................................................................................................................................................................
.......................................................................................................................................................................................................................................................................................................................................................
.......................................................................................................................................................................................................................................................................................................................................................
.......................................................................................................................................................................................................................................................................................................................................................
.......................................................................................................................................................................................................................................................................................................................................................
.......................................................................................................................................................................................................................................................................................................................................................
.......................................................................................................................................................................................................................................................................................................................................................
.......................................................................................................................................................................................................................................................................................................................................................
.......................................................................................................................................................................................................................................................................................................................................................
.......................................................................................................................................................................................................................................................................................................................................................
.......................................................................................................................................................................................................................................................................................................................................................
.......................................................................................................................................................................................................................................................................................................................................................



//
>PHAS69 excise_beg:1188846      excise_end:1189218      excise_len:373  Phased_ratio:0.587      Phased_num:5    phased_abun:949
............................................................GTGGTTAATAGAATTCGGCTT......................................................................................................................................................................................................................................................................
................................................................................................................................................TCTATGACAATTAGAAATCTC..................................................................................................................................................................................
.........................................................................................................................................................................................................................................................TCAAGAAGCATTTGTCTCTGA.........................................................................
GACTTGCTATGAGGAGGAAAATCAGGACAACTGACATAATCAGCAATATTCTTGAGCCGAGTGGTTAATAGAATTCGGCTTTTATTGTCATCGTTAGGAAATATTCTTTGTATTTGGTCCCAAGCTTTTGTACTCCAAATATCATCTATGACAATTAGAAATCTCCTACCCTTCAGACCTTTATACGCAATCTCCATTAGTTGATCATCGCTCATTTCTTGCTTGCTTCCAGTAACTGAAGAGACAACTTCAAGAAGCATTTGTCTCTGATTGTATTCTTTGGAGATAGTGACCCATGCATGTTTATCAAATCGAGAACGAATATAGGAATCTCCAAAAACTT
CTGAACGATACTCCTCCTTTTAGTCCTGTTGACTGTATTAGTCGTTATAAGAACTCGGCTCACCAATTATCTTAAGCCGAAAATAACAGTAGCAATCCTTTATAAGAAACATAAACCAGGGTTCGAAAACATGAGGTTTATAGTAGATACTGTTAATCTTTAGAGGATGGGAAGTCTGGAAATATGCGTTAGAGGTAATCAACTAGTAGCGAGTAAAGAACGAACGAAGGTCATTGACTTCTCTGTTGAAGTTCTTCGTAAACAGAGACTAACATAAGAAACCTCTATCACTGGGTACGTACAAATAGTTTAGCTCTTGCTTATATCCTTAGAGGTTTTTGAA
.......................................................................................................................................................................................................................................................GAAGTTCTTCGTAAACAGAGA...........................................................................
.................................................................................................................................................................................................................................................................................................ACTGGGTACGTACAAATAGTT.................................



//
>PHAS43 excise_beg:2501038      excise_end:2502355      excise_len:1318 Phased_ratio:0.332      Phased_num:34   phased_abun:6548
............................................................TTGAGAAACATGCATGGGTCA......................................................................................................................................................................................................................................................................
......................................................................................................AGAGACAGATGCTTCTTGAGG............................................................................................................................................................................................................................
................................................................................................................................................TCAACAATCAAGAAATGAGCA..................................................................................................................................................................................
.....................................................................................................................................................................ATGATGAATTGATGGTTATTG.............................................................................................................................................................
..........................................................................................................................................................................................TGTATAGAAGTCTCAAGGGTA........................................................................................................................................
.........................................................................................................................................................................................................................................................CTTGGGACCAAATGCGAAGAA.........................................................................
...................................................................................................................................................................................................................................................................................................AAAGCCGAATTATACTAACCA...............................
........................................................................................................................................................................................................................................................................................................................CTCGTCTCAAACATGTTGCTG..........
.......................................................................................................................................................................................................................................................................................................................................................
.......................................................................................................................................................................................................................................................................................................................................................
.......................................................................................................................................................................................................................................................................................................................................................
.......................................................................................................................................................................................................................................................................................................................................................
.......................................................................................................................................................................................................................................................................................................................................................
.......................................................................................................................................................................................................................................................................................................................................................
.......................................................................................................................................................................................................................................................................................................................................................
.......................................................................................................................................................................................................................................................................................................................................................
.......................................................................................................................................................................................................................................................................................................................................................
.......................................................................................................................................................................................................................................................................................................................................................
.......................................................................................................................................................................................................................................................................................................................................................
GTATAGGTAAGACAACTCTTGCTAGAAAAGTTTTTGATAATTCAATCATTCGTGATCGATTTGAGAAACATGCATGGGTCACCATATCTGAACAATATAATAAGAGACAGATGCTTCTTGAGGTTGCTTCTTCAATTAGTGGAGTCAACAATCAAGAAATGAGCAATGATGAATTGATGGTTATTGTGTATAGAAGTCTCAAGGGTAGGAGATTTCTAATCGTCATAGATGATCTTTGGAGTACTGAGGCTTGGGACCAAATGCGAAGAATATTTCAAAATGATAACAATAAAAGCCGAATTATACTAACCACTCGTCTCAAACATGTTGCTGATTATGCAAG
CATATCCATTCTGTTGAGAACGATCTTTTCAAAAACTATTAAGTTAGTAAGCACTAGCTAAACTCTTTGTACGTACCCAGTGGTATAGACTTGTTATATTATTCTCTGTCTACGAAGAACTCCAACGAAGAAGTTAATCACCTCAGTTGTTAGTTCTTTACTCGTTACTACTTAACTACCAATAACACATATCTTCAGAGTTCCCATCCTCTAAAGATTAGCAGTATCTACTAGAAACCTCATGACTCCGAACCCTGGTTTACGCTTCTTATAAAGTTTTACTATTGTTATTTTCGGCTTAATATGATTGGTGAGCAGAGTTTGTACAACGACTAATACGTTC
...............................................................................GTGGTATAGACTTGTTATATT...................................................................................................................................................................................................................................................
```

```
....................................................................................................ATTCTCTGTCTACGAAGAACT..............................................................................................................................................................................................................................
..............................................................................................................................................TCAGTTGTTAGTTCTTTACTC....................................................................................................................................................................................
.............................................................................................................................................................................................................ATCCTCTAAAGATTAGCAGTA.....................................................................................................................
..................................................................................................................................................................................................................................TCTACTAGAAACCTCATGACT................................................................................................
.................................................................................................................................................................................................................................................................................................ATTTTCGGCTTAATATGATTG.................................
......................................................................................................................................................................................................................................................................................................................GTGAGCAGAGTTTGTACAACG............
...........................................................................................................................................................................................................................................................................................................................................ACTAATACGTTC
.......................................................................................................................................................................................................................................................................................................................................................
.......................................................................................................................................................................................................................................................................................................................................................
.......................................................................................................................................................................................................................................................................................................................................................
.......................................................................................................................................................................................................................................................................................................................................................
.......................................................................................................................................................................................................................................................................................................................................................
.......................................................................................................................................................................................................................................................................................................................................................
.......................................................................................................................................................................................................................................................................................................................................................



//
>PHAS64 excise_beg:7734970	excise_end:7735342	excise_len:373	Phased_ratio:0.833	Phased_num:4	phased_abun:1180
............................................................TATCGGTCCTGATCATGACGT......................................................................................................................................................................................................................................
.....................................................................................................................................................................TGATAAAATGTAGAAATTATA.............................................................................................................................................................
.........................................................................................................................................................................................................................................................TCAAGATCTGTAATGTTATTA.........................................................................
...................................................................................................................................................................................................................................................................................................TTAACGAACAGTAGATGAGTT...............................
ATTATCTACAATATCAATACCAAAGTCAATGGCTCTGGAATGCGTGCTTTACCCTTCTTGTATCGGTCCTGATCATGACGTCTATACAATACACAAAATTTATAAGGTATACAATTTGCTATAATTTCATATTGAATACAACATAATAGTAATAATACAATTTTGTGATAAAATGTAGAAATTATATAGATATCTAATGTATTTGTGAAATTATTCACCAGAATTTAGAGTAATATAACTAACAACAAATCAAGATCTGTAATGTTATTACAATACAAAATTTAAAAAAAATTAACGAACAGTAGATGAGTTGTTTCAATCATTTTACAATACAACGTACATT
TAATAGATGTTATAGTTATGGTTTCAGTTACCGAGACCTTACGCACGAAATGGGAAGAACATAGCCAGGACTAGTACTGCAGATATGTTATGTGTTTTAAATATTCCATATGTTAAACGATATTAAAGTATAACTTATGTTGTATTATCATTATTATGTTAAAACACTATTTTACATCTTTAATATATCTATAGATTACATAAACACTTTAATAAGTGGTCTTAAATCTCATTATATTGATTGTTGTTTAGTTCTAGACATTACAATAATGTTATGTTTTAAATTTTTTTTAATTGCTTGTCATCTACTCAACAAAGTTAGTAAAATGTTATGTTGCATGTAA



//
>PHAS34 excise_beg:633953	excise_end:634703	excise_len:751	Phased_ratio:0.729	Phased_num:18	phased_abun:4512
............................................................AATACTCTCATGCATTGGACA......................................................................................................................................................................................................................................
..............................................................................................................................................................................................................................................................................TCATACAATAAACTCCAACTT....................................................
........................................................................................................................................................................................................................................................................................................................ATTTGATAAGGAGGACTACCT..........
.......................................................................................................................................................................................................................................................................................................................................................
.......................................................................................................................................................................................................................................................................................................................................................
.......................................................................................................................................................................................................................................................................................................................................................
AGTGCAAAATACAGAAAGCACGCTCTTAGGTGATGAGGCAAGTGATAGTAACTCAAAGCCAATACTCTCATGCATTGGACATCAAGATCTGTGTTCATCACTGAACTTACATTCTCAGCAACACTTCTCCATCGATCCAATCTGTTACCAATTTTGGAAAGAAGTCCAGCTATAACAACAATCATTAGAGGTAACCCTTTGCATTTTGATGCAATTTGTTTCCCAAGTTGTTCAAATTCATGAGGGAAAAATCCTCTCGCAAAGACCTTTTCATACAATAAACTCCAACTTTCATCAAAATTCATGAGGCGCATTTGATAAGGAGGACTACCTGAGCTAGCAT
TCACGTTTTATGTCTTTCGTGCGAGAATCCACTACTCCGTTCACTATCATTGAGTTTCGGTTATGAGAGTACGTAACCTGTAGTTCTAGACACAAGTAGTGACTTGAATGTAAGAGTCGTTGTGAAGAGGTAGCTAGGTTAGACAATGGTTAAAACCTTTCTTCAGGTCGATATTGTTGTTAGTAATCTCCATTGGGAAACGTAAAACTACGTTAAACAAAGGGTTCAACAAGTTTAAGTACTCCCTTTTTAGGAGAGCGTTTCTGGAAAAGTATGTTATTTGAGGTTGAAAGTAGTTTTAAGTACTCCGCGTAAACTATTCCTCCTGATGGACTCGATCGTA
....................................................................................................GACTTGAATGTAAGAGTCGTT..............................................................................................................................................................................................................................
...................................................................................................................................................................CAGGTCGATATTGTTGTTAGT...............................................................................................................................................................
........................................................................................................................................................................................AATCTCCATTGGGAAACGTAA..........................................................................................................................................
...........................................................................................................................................................................................................................................................................................................................................GACTCGATCGTA
.......................................................................................................................................................................................................................................................................................................................................................
.......................................................................................................................................................................................................................................................................................................................................................
.......................................................................................................................................................................................................................................................................................................................................................
.......................................................................................................................................................................................................................................................................................................................................................
.......................................................................................................................................................................................................................................................................................................................................................
.......................................................................................................................................................................................................................................................................................................................................................
.......................................................................................................................................................................................................................................................................................................................................................
.......................................................................................................................................................................................................................................................................................................................................................



//
>PHAS76 excise_beg:14321957	excise_end:14322224	excise_len:268	Phased_ratio:0.400	Phased_num:5	phased_abun:1891
................................................................................................................................................TTTTAATTTGAAGTGGTGATG.......................................................................................................
..........................................................................................................................................................................................AGAAATGAACGGAGATCGGCT.............................................................
ATTCTTTATATCCAACTTTCCTACAAGACACCTCCCTCAAACAATTTTTCTATTGTAATTCTAATCTCTCCATCTAGTTTTGGTACGAGGAAGATGAAAAAGGTTTTGTCTGATATCATTTTTAATAAGCTTAATTAGTTTACATTTTAATTTGAAGTGGTGATGTCTACTTGAGTGATCAGAAATAGAAATGAACGGAGATCGGCTAACTTTTTAACTAAAATATCTGAAATGTCTCCGTTCATTTATATATATATATATACGGAC
TAAGAAATATAGGTTGAAAGGATGTTCTGTGGAGGGAGTTTGTTAAAAAGATAACATTAAGATTAGAGAGGTAGATCAAAACCATGCTCCTTCTACTTTTTCCAAAACAGACTATAGTAAAAATTATTCGAATTAATCAAATGTAAAATTAAACTTCACCACTACAGATGAACTCACTAGTCTTTATCTTTACTTGCCTCTAGCCGATTGAAAAATTGATTTTATAGACTTTACAGAGGCAAGTAAATATATATATATATATGCCTG
..........................................................AAGATTAGAGAGGTAGATCAA.............................................................................................................................................................................................
....................................................................................................TCCAAAACAGACTATAGTAAA...................................................................................................................................................
...................................................................................................................................................................ACAGATGAACTCACTAGTCTT....................................................................................



//
>PHAS23 excise_beg:10138806	excise_end:10139283	excise_len:478	Phased_ratio:0.415	Phased_num:13	phased_abun:1175
.................................................................................AAAGACTTACAATGAGGTGGA.................................................................................................................................................................................................................................................
......................................................................................................AACTCAGGACAGCTGACATTA............................................................................................................................................................................................................................
...........................................................................................................................TCGGCAACATACTTGAGACGA.......................................................................................................................................................................................................
................................................................................................................................................GTGGTTAATAGAATTCGGCTT..................................................................................................................................................................................
....................................................................................................................................................................................................................................TCTATGACAATTAGAAATCTC..............................................................................................
.............................................................................................................................................................................................................................................................................................................................................TCAAGAAGCA
.......................................................................................................................................................................................................................................................................................................................................................
AGTTTTACAAGTTGACGAGGACATGAATCTTTTTTGAATAATTTTTCGGTGAATAGATTCCAACTATCATCTAGACTAAGAAAAGACTTACAATGAGGTGGAAACTCAGGACAGCTGACATTATCGGCAACATACTTGAGACGAGTGGTTAATAGAATTCGGCTTTTATTCTTATCATTTGGGAATATTCTTTGCATTTGGTCCCATGCTTCAGTACTCCAAATATCATCTATGACAATTAGAAATCTCCTACCCTTGAGACCTTTATAGACAATCTCCATTAGTTGATCATCGCTCTTCTCTTGATTGCTTCCGGTAATTGAAGAGGCAACTTCAAGAAGCA
TCAAAATGTTCAACTGCTCCTGTACTTAGAAAAAACTTATTAAAAAGCCACTTATCTAAGGTTGATAGTAGATCTGATTCTTTTCTGAATGTTACTCCACCTTTGAGTCCTGTCGACTGTAATAGCCGTTGTATGAACTCTGCTCACCAATTATCTTAAGCCGAAAATAAGAATAGTAAACCCTTATAAGAAACGTAAACCAGGGTACGAAGTCATGAGGTTTATAGTAGATACTGTTAATCTTTAGAGGATGGGAACTCTGGAAATATCTGTTAGAGGTAATCAACTAGTAGCGAGAAGAGAACTAACGAAGGCCATTAACTTCTCCGTTGAAGTTCTTCGT
..........................................................AGGTTGATAGTAGATCTGATT........................................................................................................................................................................................................................................
.............................................................................................................................................................................................................TACGAAGTCATGAGGTTTATA.....................................................................................................................
............................................................................................................................................................................................................................................................................TCTGTTAGAGGTAATCAACTA......................................................
...........................................................................................................................................................................................................................................................................................................................................GAAGTTCTTCGT
.......................................................................................................................................................................................................................................................................................................................................................
.......................................................................................................................................................................................................................................................................................................................................................



//
>PHAS78 excise_beg:65155769	excise_end:65156099	excise_len:331	Phased_ratio:0.369	Phased_num:6	phased_abun:648
.................................................................................CGTTACGGTAGAGATAGAGTG.....................................................................................................................................................................................................................................
................................................................................................................................................TTGTGCACGGTTCCTCTCGGA......................................................................................................................................................................
....................................................................................................................................................................................................................................AGAAGATTGGGTTTTGGACTA..................................................................................
.........................................................................................................................................................................................................................................................TTAAACAAAGTTGCATTGTTA.............................................................
TTGCCCGGTGGAAATGGAAGATTAATTGATGCATTGGCCGAAAATGTGCCTATTAGTTTTGAAAAAACTGTGCATACCATTCGTTACGGTAGAGATAGAGTGAAGGTAATTACTGCAGGACAAGTATTTGAGGGAGATATGGTATTGTGCACGGTTCCTCTCGGAGTTCTAAAGCGTGGTTCTATCAGGTTCTTTCCAGAGTTGCCTCAACGAAAGCTGGATACAATAAGAAGATTGGGTTTTGGACTATTAAACAAAGTTGCATTGTTATTCCCGTATGTGTTTTGGGACTCCAACATTGATACATTTGGTCATGTTGCTGATGATTCT
AACGGGCCACCTTTACCTTCTAATTAACTACGTAACCGGCTTTTACACGGATAATCAAAACTTTTTTGACACGTATGGTAAGCAATGCCATCTCTATCTCACTTCCATTAATGACGTCCTGTTCATAAACTCCCTCTATACCATAACACGTGCCAAGGAGAGCCTCAAGATTTCGCACCAAGATAGTCCAAGAAAGGTCTCAACGGAGTTGCTTTCGACCTATGTTATTCTTCTAACCCAAAACCTGATAATTTGTTTCAACGTAACAATAAGGGCATACACAAAACCCTGAGGTTGTAACTATGTAAACCAGTACAACGACTACTAAGA
..........................................................AACTTTTTTGACACGTATGGT............................................................................................................................................................................................................................................................
...................................................................................................................................................................CTCAAGATTTCGCACCAAGAT...................................................................................................................................................



//
>PHAS46 excise_beg:31461215	excise_end:31461734	excise_len:520	Phased_ratio:0.501	Phased_num:11	phased_abun:25287
............................................................AGGTGGATGAAATACACTGCA......................................................................................................................................................................................................................................
......................................................................................................ACTTTGATTGCCACGATGATG............................................................................................................................................................................................................................
...........................................................................................................................TTTGTTGCTGGCTTCACTGTG.......................................................................................................................................................................................................
................................................................................................................................................TGAGATGGTAATAACGGTGAT..................................................................................................................................................................................
.....................................................................................................................................................................AATGGTATTCTAATATTGCTA.............................................................................................................................................................
...................................................................................................................................................................................................................................................................................................TTAGCAATATTGGAATACCAT...............................
.............................................................................................................................................................................................................................................................................................................................................GCACAGTGAA
.......................................................................................................................................................................................................................................................................................................................................................
AGGGAAATCCCTATGGAATTATTCACAAAAGAGAACAAGCTTTTACCAAAAGAAGGAGAAAGGTGGATGAAATACACTGCAAATTCTTGTATGATTGTTGCAACTTTGATTGCCACGATGATGTTTGTTGCTGGCTTCACTGTGTGAGATGGTAATAACGGTGATAATGGTATTCTAATATTGCTAAAGTTAGACAGATTTACTGCCTTTTGTTATTTCAGATGCAGGAACACCAATACAAGAAGCAATGAGCTTGCTTGGGCATGTCAAAAGCCGTAAATCCATTCAATTTTAGCAATATTGGAATACCATCATCACCGTTATTACCACCTGGCACAGTGAA
TCCCTTTAGGGATACCTTAATAAGTGTTTTCTCTTGTTCGAAAATGGTTTTCTTCCTCTTTCCACCTACTTTATGTGACGTTTAAGAACATACTAACAACGTTGAAACTAACGGTGCTACTACAAACAACGACCGAAGTGACACACTCTACCATTATTGCCACTATTACCATAAGATTATAACGATTTCAATCTGTCTAAATGACGGAAAACAATAAAGTCTACGTCCTTGTGGTTATGTTCTTCGTTACTCGAACGAACCCGTACAGTTTTCGGCATTTAGGTAAGTTAAAATCGTTATAACCTTATGGTAGTAGTGGCAATAATGGTGGACCGTGTCACTT
............................................................................................................................................................................................................................................................................TTTTCGGCATTTAGGTAAGTT......................................................
.......................................................................................................................................................................................................................................................................................................................................................
.......................................................................................................................................................................................................................................................................................................................................................
```

Genome-wide identification and characterization of phased small interfering RNA genes in response to Botrytis cinerea infection in Solanum lycopersicum
Weibo Jin, Yue Chen, Xiaole Zhu , Xin Tian, Fangli Wu

Table S2 Annotation and transcriptional analysis of identified 91 PHASs

No.	ID	Chr	Beg-End	Phased_Ratio	Phased_Abundance	Phased_Number	Phased_Score	Transcriptome	RT-PCR	Location	Gene_ID	Annotation
1	Sly-PHAS39	8	61096825-61098289	0.689	122160	51	411.48	yes	no	Intergenic	-	-
2	Sly-PHAS40	1	3821576-3822410	0.83	209390	31	315.319	yes	no	Intergenic	-	-
3	Sly-PHAS07	4	356043-356919	0.606	19615	33	197.546	Yes	Yes	Exon	Solyc04g005540.2	Cc-nbs-lrr, resistance protein
4	Sly-PHAS12	6	423568-424045	0.944	46628	19	192.911	yes	Yes	Intergenic	-	-
5	Sly-PHAS28	11	51384504-51385716	0.345	19905	50	170.926	yes	Yes	Intergenic	-	-
6	Sly-PHAS34	12	633953-634703	0.729	4512	18	110.383	yes	Yes	Intergenic	-	-
7	Sly-PHAS33	11	7149517-7150225	0.532	4421	24	107.214	Yes	Yes	Intron	Solyc11g013770.1	Unknown Protein
8	Sly-PHAS41	9	66333637-66334345	0.643	1973	21	102.393	yes	no	Intergenic	-	-
9	Sly-PHAS42	10	51458570-51459425	0.545	7460	21	102.104	yes	no	Intergenic	-	-
10	Sly-PHAS37	1	11691253-11691898	0.849	8756	13	100.237	yes	Yes	Intergenic	-	-
11	Sly-PHAS43	5	2501038-2502355	0.332	6548	34	99.08	Yes	No	Exon	Solyc05g008070.2	Cc-nbs-lrr, resistance protein
12	Sly-PHAS15	6	37325903-37326401	0.854	34411	11	98.184	Yes	Yes	Exon-Intron	Solyc06g054600.2	Zinc finger CCCH domain-containing protein 58
13	Sly-PHAS08	4	363213-364068	0.448	3165	25	90.188	Yes	Yes	Exon	Solyc04g005550.1	Cc-nbs-lrr, resistance protein
14	Sly-PHAS44	12	2778979-2779351	0.687	4968	15	87.678	Yes	No	Exon	Solyc12g009520.1	LRR receptor-like serine/threonine-protein kinase, RLP;
15	Sly-PHAS38	11	54896607-54897252	0.709	2731	15	84.092	Yes	Yes	Intron	Solyc11g071410.1	Disease resistance protein R3a-like protein
16	Sly-PHAS27	11	1182678-1183680	0.5	2880	20	79.646	Yes	Yes	Exon-Intron	Solyc11g006520.1	Cc-nbs-lrr, resistance protein
17	Sly-PHAS45	2	28907395-28908334	0.542	1070	17	64.23	Yes	No	Exon	Solyc02g032650.2	Nbs-lrr, resistance protein
18	Sly-PHAS46	11	31461215-31461734	0.501	25287	11	55.869	yes	no	Intergenic	-	-
19	Sly-PHAS47	11	53108590-53109361	0.46	1248	17	55.708	Yes	No	Exon-Intron	Solyc11g068360.1	Cc-nbs-lrr, resistance protein
20	Sly-PHAS48	11	54263089-54263986	0.455	1238	17	55.047	Yes	No	Exon	Solyc11g069620.1	Cc-nbs-lrr, resistance protein
21	Sly-PHAS13	6	2161857-2162313	0.773	227	12	50.304	Yes	Yes	Exon	Solyc06g008300.2	LRR receptor-like serine/threonine-protein kinase, RLP
22	Sly-PHAS49	11	1242990-1243341	0.694	6532	8	48.768	Yes	No	Exon-Intron	Solyc11g006640.1	Cc-nbs-lrr, resistance protein
23	Sly-PHAS30	11	54580204-54580912	0.33	1254	19	44.769	yes	Yes	Intergenic	-	-
24	Sly-PHAS11	5	3823470-3824094	0.457	865	14	43.29	Yes	Yes	Intron	Solyc05g009630.2	Disease resistance protein
25	Sly-PHAS50	5	264321-264861	0.47	698	14	43.074	Yes	No	Exon	Solyc05g005330.2	Cc-nbs-lrr, resistance protein with an R1 specific domain
26	Sly-PHAS51	1	65142532-65142904	0.751	778	8	39.991	yes	no	Intergenic	-	
27	Sly-PHAS23	11	10138806-10139283	0.415	1175	13	38.159	Yes	Yes	Exon	Solyc11g020100.1	Cc-nbs-lrr, resistance protein
28	Sly-PHAS52	6	37335120-37335597	0.367	1408	14	37.282	Yes	No	Exon-Intron	Solyc06g054620.2	Zinc finger CCCH domain-containing protein 34
29	Sly-PHAS53	11	11186857-11187145	0.953	1978	5	36.158	yes	no	Intergenic	-	-
30	Sly-PHAS54	1	87425595-87425883	0.907	735	6	35.901	Yes	No	Intron	Solyc01g096350.2	Calcium dependent protein kinase-like protein
31	Sly-PHAS55	9	13623048-13623420	0.902	278	7	35.524	Yes	No	Exon	Solyc09g018220.1	Cc-nbs-lrr, resistance protein
32	Sly-PHAS18	7	3097032-3097299	0.977	379	6	34.822	Yes	Yes	Exon-Intron	Solyc07g008320.2	Calcium-transporting ATPase 1
33	Sly-PHAS56	9	70361711-70362020	0.785	4601	5	33.115	yes	no	Intergenic	-	-
34	Sly-PHAS57	8	60064409-60064886	0.731	139	9	32.443	Yes	No	Exon	Solyc08g076000.2	Cc-nbs-lrr, resistance protein
35	Sly-PHAS03	2	28004455-28004806	0.932	307	6	32.041	Yes	Yes	Intron	Solyc02g032200.2	Tir-lrr, resistance protein fragment
36	Sly-PHAS02	1	1897637-1898156	0.476	1635	9	31.682	Yes	Yes	Exon	Solyc01g007690.2	Unknown Protein
37	Sly-PHAS04	3	33137543-33138062	0.454	742	10	30.02	yes	Yes	Intergenic	-	-
38	Sly-PHAS26	11	18740259-18740610	0.647	10225	5	29.872	yes	Yes	Intergenic	-	-
39	Sly-PHAS58	5	844677-845112	0.306	1421	13	28.835	Yes	No	Exon	Solyc05g006140.1	UDP-glucosyltransferase family 1 protein
40	Sly-PHAS21	10	2340887-2341322	0.725	134	8	28.402	Yes	Yes	Exon	Solyc10g008230.1	Cc-nbs-lrr, resistance protein
41	Sly-PHAS59	12	66918286-66918700	0.595	361	8	28.008	Yes	No	Exon	Solyc12g100010.1	LRR receptor-like serine/threonine-protein kinase, RLP
42	Sly-PHAS60	12	2986223-2986595	0.913	372	5	27.024	Yes	No	Exon	Solyc12g009770.1	LRR receptor-like serine/threonine-protein kinase, RLP;
43	Sly-PHAS61	3	3344921-3345398	0.47	2545	7	25.802	yes	no	Intergenic	-	-
44	Sly-PHAS62	1	1125523-1125895	0.508	998	7	24.566	Yes	No	Exon	Solyc01g006550.2	LRR receptor-like serine/threonine-protein kinase, RLP
45	Sly-PHAS20	8	2161869-2162199	0.45	2026	7	23.971	Yes	Yes	Exon	Solyc08g007630.1	Cc-nbs-lrr, resistance protein
46	Sly-PHAS63	12	50481440-50481875	0.489	455	8	23.957	yes	no	Intergenic	-	-
47	Sly-PHAS64	7	7734970-7735342	0.833	1180	4	23.56	yes	no	Intergenic	-	-
48	Sly-PHAS09	5	990328-990679	0.975	124	5	23.487	Yes	Yes	Exon	Solyc05g006340.2	WD-40 repeat family protein
49	Sly-PHAS22	10	2347115-2347403	0.875	200	5	23.171	Yes	Yes	Exon	Solyc10g008240.2	Cc-nbs-lrr, resistance protein
50	Sly-PHAS10	5	63987877-63988291	0.587	245	7	22.623	Yes	Yes	Exon	Solyc05g054010.2	Cc-nbs-lrr, resistance protein
51	Sly-PHAS65	1	3979632-3979920	0.763	1468	4	22.247	Yes	No	Exon	Solyc01g009700.1	LRR receptor-like serine/threonine-protein kinase, RLP
52	Sly-PHAS66	7	30150665-30150890	0.634	4744	4	21.451	no	no	Intergenic	-	-
53	Sly-PHAS67	9	4209688-4209997	0.602	1155	5	21.232	no	no	Intergenic	-	-
54	Sly-PHAS68	8	58953865-58954258	0.502	1047	6	20.962	no	no	Intergenic	-	-
55	Sly-PHAS14	6	12309851-12310139	0.599	1073	5	20.908	no	yes	Intergenic	-	-
56	Sly-PHAS06	3	33140166-33140412	0.561	1610	5	20.705	Yes	Yes	Intron	Solyc03g063080.1	Unknown Protein
57	Sly-PHAS69	11	1188846-1189218	0.587	949	5	20.122	Yes	No	Exon	Solyc11g006530.1	Cc-nbs-lrr, resistance protein
58	Sly-PHAS36	12	2992369-2992699	0.659	124	6	19.052	Yes	Yes	Exon	Solyc12g009780.1	LRR receptor-like serine/threonine-protein kinase, RLP;
59	Sly-PHAS01	1	88580778-88581066	0.6	464	5	18.431	yes	no	Intergenic	-	-
60	Sly-PHAS29	11	54529614-54529902	0.625	335	5	18.179	Yes	Yes	Intron	Solyc11g069930.1	Disease resistance protein R3a-like protein
61	Sly-PHAS70	1	88582539-88582806	0.551	486	5	17.058	Yes	No	Exon	Solyc01g097890.2	Os03g0370250 protein
62	Sly-PHAS71	6	421966-422170	0.653	179	5	16.949	Yes	No	Exon	Solyc06g005410.2	Unknown Protein
63	Sly-PHAS25	11	18662824-18663154	0.687	464	4	16.883	no	Yes	Intergenic	-	-
64	Sly-PHAS72	11	2717464-2717710	0.353	397	8	16.876	Yes	No	Exon-Intron	Solyc11g008540.1	Ribonuclease 3-like protein 3
65	Sly-PHAS73	1	491972-492344	0.614	863	4	16.602	Yes	No	Exon	Solyc01g005730.2	LRR receptor-like serine/threonine-protein kinase, RLP
66	Sly-PHAS24	11	18645430-18645718	0.326	1372	7	16.509	yes	Yes	Intergenic	-	-
67	Sly-PHAS74	6	704592-704880	0.418	2440	5	16.313	yes	no	Intergenic	-	-
68	Sly-PHAS75	6	705061-705328	0.493	2433	4	15.365	yes	no	Intergenic	-	-
69	Sly-PHAS76	8	14321957-14322224	0.4	1891	5	15.083	yes	no	Intergenic	-	-
70	Sly-PHAS05	3	33139124-33139433	0.528	1052	4	14.691	yes	Yes	Intergenic	-	-
71	Sly-PHAS77	4	4340342-4340651	0.775	102	4	14.332	Yes	No	Exon	Solyc04g011980.1	Cc-nbs-lrr, resistance protein
72	Sly-PHAS78	5	65155769-65156099	0.369	648	6	14.331	Yes	No	Exon	Solyc05g055580.1	Lysine-specific histone demethylase 1
73	Sly-PHAS79	11	33695519-33695807	0.594	362	4	13.998	Yes	No	Intron	Solyc11g043070.1	Cc-nbs-lrr, resistance protein
74	Sly-PHAS80	10	59189313-59189664	0.32	401	7	13.44	Yes	No	Exon-Intron	Solyc10g076270.1	WD repeat-containing protein mip1
75	Sly-PHAS17	7	61236491-61236821	0.659	164	4	13.44	yes	yes	Intergenic	-	-
76	Sly-PHAS32	11	2719362-2719587	0.572	276	4	12.858	Yes	Yes	Exon-Intron	Solyc11g008540.1	Ribonuclease 3-like protein 3
77	Sly-PHAS81	9	4127097-4127343	0.648	123	4	12.477	Yes	No	Intron	Solyc09g010830.2	Ubiquitin ligase E3
78	Sly-PHAS16	6	37334710-37335040	0.319	555	6	12.105	Yes	Yes	Exon-Intron	Solyc06g054620.2	Zinc finger CCCH domain-containing protein 34
79	Sly-PHAS82	11	2700537-2700762	0.422	938	4	11.543	Yes	No	Exon-Intron	Solyc11g008530.1	Ribonuclease 3-like protein 3
80	Sly-PHAS19	8	38677752-38678040	0.363	2614	4	11.436	yes	Yes	Intergenic	-	-
81	Sly-PHAS83	6	11227196-11227400	0.444	568	4	11.261	yes	no	Intergenic	-	-
82	Sly-PHAS84	x	18430364-18430673	0.573	105	4	10.659	yes	no	Intergenic	-	-
83	Sly-PHAS85	2	29736845-29737175	0.306	640	5	9.882	no	no	Intergenic	-	-
84	Sly-PHAS86	8	58951365-58951674	0.33	283	5	9.313	yes	no	Intergenic	-	-
85	Sly-PHAS35	12	65690087-65690417	0.464	125	4	8.962	yes	yes	Intergenic	-	-
86	Sly-PHAS87	3	3344007-3344274	0.327	194	5	8.601	yes	no	Intergenic	-	-
87	Sly-PHAS88	9	9815931-9816387	0.307	103	6	8.544	no	no	Intergenic	-	-
88	Sly-PHAS89	6	32069642-32069888	0.334	223	4	7.231	Yes	No	Exon	Solyc06g048960.2	Ribonuclease 3-like protein 3
89	Sly-PHAS90	8	58954978-58955287	0.318	181	4	6.616	no	no	Intergenic	-	-
90	Sly-PHAS91	10	61676305-61676559	0.31	171	4	6.379	yes	no	Intergenic	-	-
91	Sly-PHAS31	11	2704350-2704554	0.315	139	4	6.217	Yes	Yes	Exon	Solyc11g008530.1	Ribonuclease 3-like protein 3

Genome-wide identification and characterization of phased small interfering RNA genes in response to Botrytis cinerea infection in Solanum lycopersicum

Weibo Jin, Yue Chen, Xiaole Zhu , Xin Tian, Fangli Wu

Table S3 miRNA binding sites were predicted in 91 PHAS loci via psRNAtarget online

miRNA_Acc.	Target_Ac	Expectatio	UPE	miRNA_st	miRNA_er	Target_sta	Target_end	miRNA_aligned_fragment	Target_aligned_fragment	Inhibition	Target_De	Multiplicity
sly-miR390a-5p	PHAS51	1.5	10.562	1	21	48	68	AAGCUCAGGAGGGAUAGCACC	GGUGCUAUCCUACCUGAGCUU	Translation		1
sly-miR390b-5p	PHAS51	2	10.562	1	21	48	68	AAGCUCAGGAGGGAUAGCGCC	GGUGCUAUCCUACCUGAGCUU	Translation		1
sly-miR6023	PHAS37	1	19.085	1	22	49	70	UUCCAUGAAAGAGUUUUUGGAU	AUCUAAAAACUCUUUCGUGGAA	Cleavage		1
sly-miR482a	PHAS07	3	20.012	1	20	49	68	UUUCCAAUUCCACCCAUUCC	GGUAUGGGUGGAGUUGGUAA	Cleavage		1
sly-miR6024	PHAS50	3	16.815	1	22	24	45	UUUUAGCAAGAGUUGUUUUACC	GGUAAGACAACUCUCGCUAACA	Cleavage		1
sly-miR6024	PHAS43	1	13.195	1	22	6	27	UUUUAGCAAGAGUUGUUUUACC	GGUAAGACAACUCUUGCUAGAA	Cleavage		1
sly-miR482d-3p	PHAS71	1	11.427	1	21	136	156	UUUCCUAUUCCACCCAUGCCA	UGGCAUUGGUGGAAUAGGAAA	Cleavage		1
sly-miR4376	PHAS18	3	16.487	1	22	29	50	ACGCAGGAGAGAUGAUGCUGGA	UCUGGUGUCAUCUCUCCUGCGA	Cleavage		1
sly-miR482e-3p	PHAS39	1.5	10.951	1	22	19	40	UCUUUCCUACUCCUCCCAUACC	GGUAUGGAGGGAGUAGGAAAGA	Cleavage		1
sly-miR482d-3p	PHAS39	3	10.883	1	21	18	38	UUUCCUAUUCCACCCAUGCCA	UGGUAUGGAGGGAGUAGGAAA	Cleavage		1
sly-miR6024	PHAS55	3	11.063	1	22	6	27	UUUUAGCAAGAGUUGUUUUACC	GGGAAAACAACUCUUGCCAAGA	Cleavage		1
sly-miR6026	PHAS55	2	11.063	1	22	7	28	UUCUUGGCUAGAGUUGUAUUGC	GGAAAACAACUCUUGCCAAGAA	Translation		1
sly-miR6024	PHAS21	2	9.717	1	22	7	28	UUUUAGCAAGAGUUGUUUUACC	GGCAAAACGACUCUUGCUAGAA	Cleavage		1
sly-miR6024	PHAS22	2	8.662	1	22	7	28	UUUUAGCAAGAGUUGUUUUACC	GGCAAAACGACUCUUGCUAGAA	Cleavage		1
sly-miR6024	PHAS23	2	22.612	1	22	29	50	UUUUAGCAAGAGUUGUUUUACC	GGUAAGACAACUCUUGCCAAAA	Cleavage		1
sly-miR482b	PHAS23	3	21.29	1	22	14	35	UCUUGCCUACACCGCCCAUGCC	GGUAUGGGCGGUAUCGGUAAGA	Translation		1
sly-miR482c	PHAS23	2.5	21.29	1	22	14	35	UCUUGCCAAUACCGCCCAUUCC	GGUAUGGGCGGUAUCGGUAAGA	Cleavage		1
sly-miR6024	PHAS69	2.5	20.899	1	22	8	29	UUUUAGCAAGAGUUGUUUUACC	GGUAAGACAACUCUUGCCAGAA	Cleavage		1
sly-miR6024	PHAS28	2	15.938	1	21	1	21	UUUUAGCAAGAGUUGUUUUAC	GUAAGACAACACUUGCUAAAG	Translation		1
sly-miR6024	PHAS48	3	21.229	1	22	65	86	UUUUAGCAAGAGUUGUUUUACC	GGUAAGACAACACUAGCUAAAG	Translation		1
sly-miR6024	PHAS29	3	19.594	1	22	7	28	UUUUAGCAAGAGUUGUUUUACC	GGUAAGACAACACUAGCUAAAG	Translation		1
sly-miR482e-3p	PHAS33	2.5	10.248	1	22	28	49	UCUUUCCUACUCCUCCCAUACC	GGUAUGGAAGGAGUAAGAAAGA	Cleavage		1
sly-miR169b	PHAS34	3	14.338	1	21	66	86	UAGCCAAGGAUGACUUGCCUG	UAGGUAAGACAACUUUGGCUA	Translation		1
sly-miR169d	PHAS34	3	14.338	1	21	66	86	UAGCCAAGGAUGACUUGCCUA	UAGGUAAGACAACUUUGGCUA	Translation		1
sly-miR482c	PHAS34	3	17.897	1	22	53	74	UCUUGCCAAUACCGCCCAUUCC	GGAAUGGGUGGCAUAGGUAAGA	Translation		1
sly-miR169e-5p	PHAS34	3	14.338	1	22	65	86	UAGCCAAGGAUGACUUGCCUUU	AUAGGUAAGACAACUUUGGCUA	Translation		1
sly-miR6023	PHAS63	3	17.22	1	22	49	70	UUCCAUGAAAGAGUUUUUGGAU	AUCCAAAAACAAGUUCAUGGAA	Translation		1
sly-miR6023	PHAS59	3	11.495	1	22	5	26	UUCCAUGAAAGAGUUUUUGGAU	AUCCAAAAACGAAUUCAUGGAA	Translation		1
sly-miR6023	PHAS44	2.5	19.684	1	20	51	70	UUCCAUGAAAGAGUUUUUGG	CCGAAAACAGUUUCAUGGAA	Translation		1

Genome-wide identification and characterization of phased small interfering RNA genes in response to Botrytis cinerea infection in Solanum lycopersicum

Weibo Jin, Yue Chen, Xiaole Zhu , Xin Tian, Fangli Wu

Table S4 Differential expression of 245 phasiRNAs based on sRNA-seq data

phasiRNA_id	phasiRNA_sequence	TC7d_raw	TD7d_raw	TC7d_RP10M	TC7d_RP10M	Log2(TD7d/TC7d)	Pvalue	FDR	Significance
siR11-1	AGAAGGCAATGTAGCTGA	28	28	16.6	20.1	0.28	0.4726	0.7132	
siR11-11	AATCAAGTTCAGTCGATTT	17	29	10.1	20.8	1.04	0.0161	0.0651	
siR11-13	TCAAGAAATCAAACAAGG	43	46	25.5	33.0	0.37	0.2236	0.4938	
siR11-14	TGAGTTCTGCAGACACGA	684	671	406.1	481.5	0.25	0.0017	0.0106	
siR05-1	GAGTGCTTGAAATTGTCG	56	81	33.2	58.1	0.81	0.0012	0.0078	
siR31-3	AAGAACTTGAGACGGAAA	100	188	59.4	134.9	1.18	0.0000	0.0000	**
siR31-4	TCTGCGACCTTGAGTAGA	13	35	7.7	25.1	1.70	0.0001	0.0009	**
siR72-4	ATACTTTAGCTCATGACCT	60	64	35.6	45.9	0.37	0.1560	0.4042	
siR72-6	TTCGGATTTGAGAATGGA	65	111	38.6	79.7	1.05	0.0000	0.0000	**
siR53-5	AGAAGATCATGAATTTTG	650	983	385.9	705.4	0.87	0.0000	0.0000	
siR55-1	TTCGAGACGTGATTATCAT	18	43	10.7	30.9	1.53	0.0001	0.0006	**
siR55-4	AGAGTGATCGCAGGTTGT	39	79	23.2	56.7	1.29	0.0000	0.0000	**
siR55-7	TCTGGACTGGTCTACGTTT	263	299	156.1	214.6	0.46	0.0002	0.0013	
siR24-3	TGAAACTTAAAGGAATTG	564	408	334.8	292.8	-0.19	0.0388	0.1319	
siR48-2	TAAAGCGGTTTACAATGA	65	46	38.6	33.0	-0.23	0.4252	0.6572	
siR48-5	TATGGGAAGTAAGATCAT	56	39	33.2	28.0	-0.25	0.4171	0.6500	
siR48-8	TCCGGAACTTGAAGAGGT	1168	1032	693.4	740.5	0.09	0.1234	0.3382	
siR48-9	AAGTGAAATATGGGACCA	18	31	10.7	22.2	1.05	0.0119	0.0505	
siR13-8	ATATGAAGGTAATGATGG	9	30	5.3	21.5	2.02	0.0001	0.0006	**
siR67-4	GGGCACGTGTCGTTGGCT	764	121	453.6	86.8	-2.39	0.0000	0.0000	**
siR42-2	CAGAGGTCTGAAGGGTAG	361	477	214.3	342.3	0.68	0.0000	0.0000	
siR42-3	TATTTGGAGTACTGAGGC	497	502	295.0	360.2	0.29	0.0016	0.0101	
siR42-4	AAGTCGAGATGATAGTTG	37	29	22.0	20.8	-0.08	0.8409	0.9167	
siR42-5	TCTATTCATCGAAAGACTA	75	71	44.5	50.9	0.19	0.4121	0.6435	
siR42-7	TTTACTTGTAGAAATAGG	132	137	78.4	98.3	0.33	0.0626	0.2004	
siR42-8	AGGATTACCTCTCTCGATT	38	16	22.6	11.5	-0.97	0.0209	0.0800	
siR42-13	AGTAGTTAATAGAATTCG	1910	1747	1133.9	1253.6	0.14	0.0024	0.0137	
siR42-15	AAAATCAGGGCAGTTGAC	366	349	217.3	250.4	0.20	0.0575	0.1857	
siR42-16	CAAAGACTTACTATGAGG	332	145	197.1	104.0	-0.92	0.0000	0.0000	
siR42-20	AAGAAGATCACGAATTTT	42	44	24.9	31.6	0.34	0.2712	0.5555	
siR84-2	TTAAATAAATGGATTGTTC	35	40	20.8	28.7	0.46	0.1610	0.4131	
siR44-7	GGTCCAATCCGATAACGT	18	50	10.7	35.9	1.75	0.0000	0.0000	**
siR44-11	AGTTGTAGTCGGCTTAAT	39	86	23.2	61.7	1.41	0.0000	0.0000	**
siR44-13	ACTTGTGACATGTTAACC	108	197	64.1	141.4	1.14	0.0000	0.0000	**
siR44-14	TGCTGCACTTGGGACGGG	16	33	9.5	23.7	1.32	0.0020	0.0118	*
siR44-15	TCATGGAATGAGAGTAAC	120	379	71.2	272.0	1.93	0.0000	0.0000	**
siR41-1	TCCCTTTGGATGTCTTCTT	52	45	30.9	32.3	0.06	0.8163	0.9081	
siR41-2	TCAGTAAGTTTTGAAGCAT	291	308	172.8	221.0	0.35	0.0026	0.0141	
siR41-7	TATCGAACAGTAGCTGGA	76	60	45.1	43.1	-0.07	0.7954	0.8874	
siR41-10	TCCAGCAAGACTTCACAT	7	28	4.2	20.1	2.26	0.0000	0.0003	**
siR41-12	ATGCTTCAAAACTTACTGA	20	36	11.9	25.8	1.12	0.0044	0.0221	*
siR41-13	TCTTCTGAGCTAGCATCTT	52	38	30.9	27.3	-0.18	0.5714	0.7706	
siR54-2	TGGGGACGGTCTTTCCATA	49	40	29.1	28.7	-0.02	0.9609	0.9750	
siR54-3	CTCCATCAGAATTGCGGA	94	70	55.8	50.2	-0.15	0.5119	0.7305	
siR54-4	TGCGGCGACGGATGATCG	37	50	22.0	35.9	0.71	0.0228	0.0859	
siR08-3	TCTGGGAGGCTCTTCATGA	34	58	20.2	41.6	1.04	0.0006	0.0045	**
siR08-6	AATCAATGGAAGCTCAGA	5	30	3.0	21.5	2.84	0.0000	0.0000	**
siR08-7	TCATGGAAGTTGGAACCT	20	32	11.9	23.0	0.95	0.0191	0.0735	
siR08-18	CAAACATGTCGGAAACGC	1476	1220	876.2	875.4	0.00	0.9830	0.9882	
siR75-1	TAGCTTGACGTATAGAACA	439	542	260.6	388.9	0.58	0.0000	0.0000	
siR75-3	GATAGAGCAAGGATTGTA	28	31	16.6	22.2	0.42	0.2612	0.5543	
siR52-2	CAAAGGAAAAGGTCCAGC	85	60	50.5	43.1	-0.23	0.3519	0.6400	
siR52-5	CACTTATCCAGAACTGCGA	21	34	12.5	24.4	0.96	0.0142	0.0584	
siR52-9	TATGAAAACAGGGCACTG	141	107	83.7	76.8	-0.12	0.5059	0.7274	
siR52-10	TGAAAAGGAATGCGCACA	493	947	292.7	679.5	1.22	0.0000	0.0000	**
siR52-11	TTCATTGAGTTCGTCACAC	120	209	71.2	150.0	1.08	0.0000	0.0000	**
siR52-14	TTTTTAATTCTTTTGTTGG	151	259	89.6	185.9	1.05	0.0000	0.0000	**
siR15-2	TATGAAAACAGGGCACTG	141	107	83.7	76.8	-0.12	0.5059	0.7260	
siR15-6	AGCTGGCTGTGGATGGTTA	571	1135	339.0	814.4	1.26	0.0000	0.0000	**
siR15-7	TAATGCTGGAACTTGCACA	26	34	15.4	24.4	0.66	0.0773	0.2374	
siR15-8	CAAAGGAAAAGGTCCAGC	85	60	50.5	43.1	-0.23	0.3519	0.6415	
siR15-9	TTGCAGTTCTGGATAAGT	79	70	46.9	50.2	0.10	0.6698	0.8076	
siR15-10	TATAGAAACCTGGAAGCA	26	45	15.4	32.3	1.07	0.0023	0.0131	*
siR19-4	ATAACATTATAGGATTTC	417	310	247.6	222.4	-0.15	0.1545	0.4019	
siR26-2	GCTTTTAGGACTCCGCCG	2047	601	1215.2	431.3	-1.49	0.0000	0.0000	**
siR26-4	TCTTGGATTTATGAAAGA	1558	750	924.9	538.2	-0.78	0.0000	0.0000	
siR47-3	TGGGAAGTAAGATCATCG	86	101	51.1	72.5	0.50	0.0166	0.0671	
siR47-6	TCCGGAACTTGAAGAGGT	1168	1032	693.4	740.5	0.09	0.1234	0.3370	

siR47-14	TCAGCACACATCGTCCGA	109	71	64.7	50.9	-0.35	0.1177	0.3237	
siR89-4	TCATGACCTGGAACAGTT	113	106	67.1	76.1	0.18	0.3502	0.6415	
siR02-6	AAAGCGTCTGTAGGTGGC	236	206	140.1	147.8	0.08	0.5706	0.7709	
siR38-2	TCCGGAACTTGAAGAGGT	195	131	115.8	94.0	-0.30	0.0652	0.2070	
siR38-5	AAGTGAAATATGGGAGCT	26	29	15.4	20.8	0.43	0.2659	0.5629	
siR38-9	TTGTAAGCGGCTTTAGCA	290	174	172.2	124.9	-0.46	0.0007	0.0051	
siR38-14	CCTTTTAGAAGGATTTGG	0	33	0.1	23.7	7.89	0.0000	0.0000	**
siR66-1	CGGATTCTGACTTAGAGG	4997	1031	2966.5	739.8	-2.00	0.0000	0.0000	**
siR66-2	AGGGCACGTGTCGTTGGC	61	29	36.2	20.8	-0.80	0.0126	0.0532	
siR66-4	GCCTCTAAGTCAGAATCC	40	27	23.7	19.4	-0.29	0.4243	0.6598	
siR29-1	TTTGGTTTGAAAGCTTGGT	139	130	82.5	93.3	0.18	0.3127	0.6063	
siR04-4	AGGGACTACGGCCTTTTA	416	494	247.0	354.5	0.52	0.0000	0.0000	
siR04-5	CGCGGAAGTTTGAGGCAA	140	67	83.1	48.1	-0.79	0.0002	0.0013	
siR79-1	ATGAGTGGGATGACTTGA	1083	1113	642.9	798.7	0.31	0.0000	0.0000	
siR16-1	GTAGTTTGGGATGGCACT	18	40	10.7	28.7	1.42	0.0003	0.0022	**
siR16-5	ACGCAAGCCCATATGCAT	8	61	4.7	43.8	3.22	0.0000	0.0000	**
siR16-6	ACTCTGCAGCTGGAACAG	640	514	379.9	368.8	-0.04	0.6189	0.7619	
siR60-1	TGAGGAATTTGAGGCATC	74	127	43.9	91.1	1.05	0.0000	0.0000	**
siR01-1	TCCAGAGGCTTTGGACGG	203	116	120.5	83.2	-0.53	0.0013	0.0087	
siR01-4	TCATAAAAAGCAGTCGTG	98	72	58.2	51.7	-0.17	0.4504	0.6824	
siR12-3	TCTTTGCTCTAAGGCCACA	186	243	110.4	174.4	0.66	0.0000	0.0000	
siR12-4	TCAAATGTTTGTGTTGGAT	4612	4878	2738.0	3500.3	0.35	0.0000	0.0000	
siR12-7	TTGTTAAGGACTCTAATCC	2563	1787	1521.5	1282.3	-0.25	0.0000	0.0000	
siR12-9	TCTTGAGAATTTGATGAA	733	555	435.2	398.3	-0.13	0.1156	0.3192	
siR12-11	ATAAGGAGACGATTGCAA	1917	1332	1138.0	955.8	-0.25	0.0000	0.0000	
siR12-13	TATTGGGAGAAGTAGTGA	474	518	281.4	371.7	0.40	0.0000	0.0001	
siR12-14	TTTTAAAGAAAGAAGAGT	110	136	65.3	97.6	0.58	0.0017	0.0103	
siR12-16	TGTGGCCTTAGAGCAAAG	425	587	252.3	421.2	0.74	0.0000	0.0000	
siR07-1	GAGAAAATCAGACAAATG	175	139	103.9	99.7	-0.06	0.7255	0.8479	
siR07-2	AAAAAAGAAAGGTTGTTT	11	28	6.5	20.1	1.63	0.0009	0.0061	**
siR07-3	AGAATTCAGGATGAGATT	2627	2078	1559.5	1491.1	-0.06	0.1266	0.3421	
siR07-6	CTAGAGAAACTTGGAATT	70	61	41.6	43.8	0.07	0.7595	0.8783	
siR07-10	TCTGAAGAGGAAGCATGG	165	224	98.0	160.7	0.71	0.0000	0.0000	
siR07-12	AATTCAGTCGACGATCCT	68	57	40.4	40.9	0.02	0.9331	0.9544	
siR07-25	TACGCAGCAGATCTCCAC	7372	5451	4376.4	3911.5	-0.16	0.0000	0.0000	
siR07-27	CCAAGATTATAAGGATGC	661	445	392.4	319.3	-0.30	0.0007	0.0051	
siR07-28	TGAATGTCACTTTGCACCC	67	51	39.8	36.6	-0.12	0.6634	0.8024	
siR07-29	AAACATGTCGGAAACGCG	811	625	481.5	448.5	-0.10	0.1832	0.4547	
siR27-3	AGAAGATCATGAATTTTG	650	983	385.9	705.4	0.87	0.0000	0.0000	
siR27-7	AAATCAGGGCAGTTGACA	458	436	271.9	312.9	0.20	0.0359	0.1272	
siR27-8	GTGGTTAATAGAATTCGG	19	60	11.3	43.1	1.93	0.0000	0.0000	**
siR27-17	GAAAAATGGACTTAACGC	22	34	13.1	24.4	0.90	0.0209	0.0796	
siR27-20	ATATTTGGAGTACTGAGG	563	657	334.2	471.4	0.50	0.0000	0.0000	
siR61-2	TAGGATTTTATATACTAGC	82	103	48.7	73.9	0.60	0.0046	0.0227	
siR61-3	TCCATAGACGAATACCCT	123	116	73.0	83.2	0.19	0.3093	0.6044	
siR61-4	TTTGCTGTACGGTTTCTTG	31	33	18.4	23.7	0.37	0.3104	0.6033	
siR61-5	AAAGTTAATTGATTGTCG	155	251	92.0	180.1	0.97	0.0000	0.0000	
siR61-6	CATGTCATCGAGGAAATT	14	36	8.3	25.8	1.64	0.0001	0.0011	**
siR61-7	TGAAAATGAACAAGACAG	155	229	92.0	164.3	0.84	0.0000	0.0000	
siR57-1	CTAGGAAAGACGACACTT	5790	5629	3437.3	4039.2	0.23	0.0000	0.0000	
siR57-5	TACTTGCTTGTCTTAGATG	94	133	55.8	95.4	0.77	0.0001	0.0005	
siR68-3	TCTTGATCGCAGTTAATCC	678	589	402.5	422.6	0.07	0.3844	0.6627	
siR30-1	TGCAGGAAGATCATTGTA	317	214	188.2	153.6	-0.29	0.0212	0.0805	
siR30-4	TTTACGTGTCGTCACAATC	116	91	68.9	65.3	-0.08	0.7113	0.8390	
siR30-16	AGGAGATGTTGGAAGTAA	478	685	283.8	491.5	0.79	0.0000	0.0000	
siR30-17	TCTGGATGATGTGTGGAA	148	120	87.9	86.1	-0.03	0.8760	0.9182	
siR50-2	TTCGGAGTGTTAAGAAGG	329	530	195.3	380.3	0.96	0.0000	0.0000	
siR50-14	ACGAGTTTAGCATGGTAG	21	31	12.5	22.2	0.83	0.0387	0.1335	
siR28-1	TGATGCTTTTGGAATAAC	1152	1235	683.9	886.2	0.37	0.0000	0.0000	
siR28-5	AAGGGAAGAAGTTTCTTG	58	74	34.4	53.1	0.63	0.0131	0.0547	
siR28-10	TAGCAGAGCAATAAACGT	210	265	124.7	190.2	0.61	0.0000	0.0000	
siR28-12	TTGGAATTTACTCAAACG	112	93	66.5	66.7	0.00	0.9722	0.9786	
siR28-14	AGAGGTTGGAAGAAAAAT	53	86	31.5	61.7	0.97	0.0001	0.0007	
siR28-17	TCCAGATTTGAAGTGATG	145	159	86.1	114.1	0.41	0.0140	0.0580	
siR28-23	CATATGTTGAAACAAAGT	65	88	38.6	63.1	0.71	0.0024	0.0137	
siR28-31	TTCCACACATCATCCAGG	29	37	17.2	26.6	0.63	0.0792	0.2415	
siR28-34	TTACTTCCTATATCTCCTT	34	56	20.2	40.2	0.99	0.0013	0.0085	
siR28-35	TTACGTGTAGTTACAATG	37	37	22.0	26.6	0.27	0.4105	0.6423	
siR28-42	AGTACCTGAATGAAACTG	680	487	403.7	349.5	-0.21	0.0149	0.0612	
siR28-46	CCATGGAATAGGACATGT	25	32	14.8	23.0	0.64	0.1004	0.2875	
siR28-49	ACACATCCGGCAACTCCA	43	23	25.5	16.5	-0.63	0.0913	0.2646	
siR32-3	TCACAGAGTAGAAAAAGT	34	51	20.2	36.6	0.86	0.0066	0.0312	
siR86-4	TCCCGTTCGAAGGATATG	146	108	86.7	77.5	-0.16	0.3821	0.6618	
siR74-2	GATAGAGCAAGGATTGTA	28	31	16.6	22.2	0.42	0.2612	0.5559	

siR74-4	TAGCTTGACGTATAGAAC	439	542	260.6	388.9	0.58	0.0000	0.0000	
siR14-2	AATTGTCGGGAGGGAAGC	180	160	106.9	114.8	0.10	0.5055	0.7282	
siR82-3	ACAACTAGTGGACGATCA	427	853	253.5	612.1	1.27	0.0000	0.0000	**
siR82-4	CCATGTAGCAGCATTCCA	21	72	12.5	51.7	2.05	0.0000	0.0000	**
siR20-1	TCTGGATGACGTGTGGAA	54	29	32.1	20.8	-0.63	0.0596	0.1917	
siR20-2	AGGAGATATAGGAAGTAA	246	382	146.0	274.1	0.91	0.0000	0.0000	
siR20-6	TTTACGTGTCGTCACAATC	116	91	68.9	65.3	-0.08	0.7113	0.8403	
siR37-1	TTTCGTGGAACAAGAGTG	23	32	13.7	23.0	0.75	0.0555	0.1840	
siR37-3	TTCTTTGTGACAGGATGA	25	54	14.8	38.7	1.39	0.0000	0.0003	**
siR83-4	CCGCGGAAGTTTGAGGCA	87	41	51.6	29.4	-0.81	0.0025	0.0135	
siR03-5	TAGCTTCTTGAATCTTGAC	25	39	14.8	28.0	0.92	0.0122	0.0517	
siR03-6	GAAGAACTCTTTTGCAGC	47	30	27.9	21.5	-0.38	0.2730	0.5548	
siR70-2	TCATAAAAAGCAGTCGTG	98	72	58.2	51.7	-0.17	0.4504	0.6810	
siR70-5	TCCAGAGGCTTTGGACGG	203	116	120.5	83.2	-0.53	0.0013	0.0086	
siR45-14	CTAACATTGTAGAGGAAG	34	34	20.2	24.4	0.27	0.4298	0.6629	
siR91-1	TGAAGGAAAATGGTAAGT	23	37	13.7	26.6	0.96	0.0111	0.0476	
siR49-3	TATTTGGAGTACTGAGGC	497	502	295.0	360.2	0.29	0.0016	0.0102	
siR49-6	AGTAGTTAATAGAATTCG	1910	1747	1133.9	1253.6	0.14	0.0024	0.0136	
siR49-8	AAAATCAGGGCAGTTGAC	366	349	217.3	250.4	0.20	0.0575	0.1865	
siR71-2	TTTTCAAGCAACATCTAG	46	290	27.3	208.1	2.93	0.0000	0.0000	**
siR71-4	TTGGTATATATGGCATTGC	82	106	48.7	76.1	0.64	0.0023	0.0130	
siR80-6	TTCTAGGTTAGTCGTTGG	51	54	30.3	38.7	0.35	0.2045	0.4680	
siR80-7	TGGCCTGAATGAAGACAT	39	31	23.2	22.2	-0.06	0.8808	0.9207	
siR06-2	TCACCGGATCATTCAATC	11	33	6.5	23.7	1.87	0.0001	0.0006	**
siR06-4	TCGCGAGAAGTCCATTGA	164	139	97.4	99.7	0.03	0.8286	0.9072	
siR56-1	TTGGGCTGTACCAGATGG	142	137	84.3	98.3	0.22	0.1980	0.4628	
siR51-1	ATAGACAAGGAAGGAGAA	162	140	96.2	100.5	0.06	0.7008	0.8396	
siR51-2	TGAAGAAGTCATGACGTA	52	25	30.9	17.9	-0.79	0.0240	0.0887	
siR51-3	AAGACGTAAGAGATAACA	657	379	390.0	272.0	-0.52	0.0000	0.0000	
siR51-4	ATGGGGTAGGTAGAGGAA	1238	727	734.9	521.7	-0.49	0.0000	0.0000	
siR51-5	AAGCGGTGAGAAAAAGCT	70	41	41.6	29.4	-0.50	0.0789	0.2415	
siR51-6	TTCTTGACCTTGTAAGACC	1383	448	821.0	321.5	-1.35	0.0000	0.0000	**
siR51-7	TTCTTGACCTTGTAAGACC	896	373	531.9	267.7	-0.99	0.0000	0.0000	
siR51-8	TTTTTTGACTTGTTGCCTT	125	72	74.2	51.7	-0.52	0.0137	0.0571	
siR25-3	ACGCAATGTGATTTCTGC	64	81	38.0	58.1	0.61	0.0107	0.0459	
siR25-4	ACAGCCAACTCAGAACTG	106	75	62.9	53.8	-0.23	0.3041	0.5956	
siR33-1	CTTTGCAAGGAATGCTGG	34	32	20.2	23.0	0.19	0.5934	0.7851	
siR33-3	TACTGCAACAACTAGAGA	1274	907	756.3	650.8	-0.22	0.0005	0.0038	
siR33-11	AAACTAGGTTGTTCCGGA	114	137	67.7	98.3	0.54	0.0031	0.0169	
siR33-14	TTAATATCAGCAAGAAAA	56	107	33.2	76.8	1.21	0.0000	0.0000	**
siR33-15	ACCAGCATTCCTTGCAAA	10	68	5.9	48.8	3.05	0.0000	0.0000	**
siR40-1	TTATGGAATACGAGTAGA	28693	41377	17033.8	29690.9	0.80	0.0000	0.0000	
siR40-2	TGATGTACATGGGATGGA	422	363	250.5	260.5	0.06	0.5836	0.7747	
siR40-4	CCAAAAACGGTCTTATGG	605	362	359.2	259.8	-0.47	0.0000	0.0000	
siR40-12	CATTTGTAACATGAGCTG	35	25	20.8	17.9	-0.22	0.5883	0.7796	
siR40-13	TGGATTTCTAGATTTATCA	10	31	5.9	22.2	1.91	0.0001	0.0007	**
siR40-15	ATCCATCCCATGTACATC	69	232	41.0	166.5	2.02	0.0000	0.0000	**
siR40-16	ATGTTTTCTAATCAAGTTG	83	108	49.3	77.5	0.65	0.0018	0.0110	
siR40-17	AGTTTTGAAGTATTGTGTC	26	46	15.4	33.0	1.10	0.0016	0.0101	*
siR40-19	TACAACAATCTCTACTCG	164	286	97.4	205.2	1.08	0.0000	0.0000	**
siR40-20	CATAGGTATAGAAGATGA	470	512	279.0	367.4	0.40	0.0000	0.0001	
siR40-24	TGTCAAAGATCTTGGAGG	92	88	54.6	63.1	0.21	0.3278	0.6093	
siR39-3	TTTTTTTCCTCGCATAGCA	50	89	29.7	63.9	1.11	0.0000	0.0001	**
siR39-4	ACATATTTCAGAGTAATG	58	68	34.4	48.8	0.50	0.0504	0.1683	
siR39-5	TGGTTTGGTCGATTGCAC	560	738	332.4	529.6	0.67	0.0000	0.0000	
siR39-6	GATAAGCGGGAAGATATG	102	179	60.6	128.4	1.08	0.0000	0.0000	**
siR39-7	TTTGGAAGGTGGAGAAGC	1986	2907	1179.0	2086.0	0.82	0.0000	0.0000	
siR39-9	TGGTAGAAATTGAAAGAT	29355	29931	17426.8	21477.6	0.30	0.0000	0.0000	
siR39-10	TTTTTGTTATTCTTATCGG	37	24	22.0	17.2	-0.36	0.3628	0.6444	
siR39-12	ACATATATGCCAAGCTTG	35	21	20.8	15.1	-0.46	0.2510	0.5419	
siR39-23	AGATGTGATTCATAGTAA	62	44	36.8	31.6	-0.22	0.4446	0.6762	
siR39-33	TTTTTGTCCAAAAAGAAT	276	177	163.8	127.0	-0.37	0.0079	0.0360	
siR39-34	ATTTGCGATGAATTCAGT	50	39	29.7	28.0	-0.09	0.7942	0.8940	
siR39-35	TCATATCTTCCCGCTTATC	34	64	20.2	45.9	1.18	0.0001	0.0006	**
siR39-36	ACAAAGCAAAAATAGGTA	120	169	71.2	121.3	0.77	0.0000	0.0001	
siR39-37	TTACCCACCTTGTCAAGTA	78	103	46.3	73.9	0.67	0.0017	0.0105	
siR39-38	ACAAGCTTGGCATATATG	28	57	16.6	40.9	1.30	0.0001	0.0005	**
siR39-46	TCTAGGTGGAGATCTTTC	340	346	201.8	248.3	0.30	0.0067	0.0314	
siR69-1	GTGGTTAATAGAATTCGG	19	60	11.3	43.1	1.93	0.0000	0.0000	**
siR69-3	TCAAGAAGCATTTGTCTC	664	465	394.2	333.7	-0.24	0.0057	0.0274	
siR69-4	AGAGACAAATGCTTCTTG	25	40	14.8	28.7	0.96	0.0087	0.0394	
siR43-2	AGAGACAGATGCTTCTTG	47	96	27.9	68.9	1.30	0.0000	0.0000	**
siR43-3	TCAACAATCAAGAAATGA	866	1129	514.1	810.1	0.66	0.0000	0.0000	
siR43-4	ATGATGAATTGATGGTTA	7	34	4.2	24.4	2.54	0.0000	0.0000	**

siR43-5	TGTATAGAAGTCTCAAGG(	88	196	52.2	140.6	1.43	0.0000	0.0000	**
siR43-6	CTTGGGACCAAATGCGAA(	867	1091	514.7	782.9	0.61	0.0000	0.0000	
siR43-7	AAAGCCGAATTATACTAA(	102	185	60.6	132.8	1.13	0.0000	0.0000	**
siR43-9	TATTCAGAAAAGATCGTT(	4751	8541	2820.5	6128.8	1.12	0.0000	0.0000	**
siR43-10	TTATTGTCATTGCTGGACT	48	70	28.5	50.2	0.82	0.0023	0.0131	
siR43-11	TTAGCAAGATAGACGTGA(	448	708	266.0	508.0	0.93	0.0000	0.0000	
siR43-13	ACTTGAAGGCGTGTTTTCT	23	40	13.7	28.7	1.07	0.0038	0.0198	*
siR43-17	ACATAAATGATCGTAGAC(	84	130	49.9	93.3	0.90	0.0000	0.0001	
siR43-19	TGGATGCACGTGAAATTG(	32	71	19.0	50.9	1.42	0.0000	0.0000	**
siR43-21	TCAAGAAGCATCTGTCTCT	55	74	32.7	53.1	0.70	0.0060	0.0284	
siR43-23	ATGACGATTAGAAATCTC(	108	134	64.1	96.2	0.59	0.0017	0.0103	
siR43-24	TCAGTACTCCAAAGATCAT	47	40	27.9	28.7	0.04	0.8854	0.9230	
siR43-25	GTTAGTATAATTCGGCTTT	38	9	22.6	6.5	-1.80	0.0002	0.0017	**
siR43-27	TCAGGGGAGCTTGCATAAT	571	660	339.0	473.6	0.48	0.0000	0.0000	
siR43-28	ATCTTTCGTAATTGTGGAC	36	37	21.4	26.6	0.31	0.3505	0.6404	
siR43-29	TGTTGTATGATGTGCTTCC	38	35	22.6	25.1	0.15	0.6392	0.7781	
siR43-31	AAACACAGAATTGCTTGG(	54	82	32.1	58.8	0.87	0.0005	0.0032	
siR64-4	TTAACGAACAGTAGATGA(	114	89	67.7	63.9	-0.08	0.6889	0.8292	
siR34-4	ACCAAGTACCTCGGGCCT	40	23	23.7	16.5	-0.52	0.1675	0.4268	
siR34-6	AAACAAGACATAACGAAC	279	590	165.6	423.4	1.35	0.0000	0.0000	**
siR34-16	AAGATGATGGGCAACTAG	25	42	14.8	30.1	1.02	0.0044	0.0218	*
siR76-2	AGAAATGAACGGAGATCG	186	200	110.4	143.5	0.38	0.0100	0.0432	
siR76-5	TTCTGATCACTCAAGTAG/	439	592	260.6	424.8	0.70	0.0000	0.0000	
siR23-3	TCGGCAACATACTTGAGA(	20	62	11.9	44.5	1.90	0.0000	0.0000	**
siR23-4	GTGGTTAATAGAATTCGG(	19	60	11.3	43.1	1.93	0.0000	0.0000	**
siR23-11	AGAGACAAATGCTTCTTG/	25	40	14.8	28.7	0.96	0.0087	0.0391	
siR46-1	AGGTGGATGAAATACACT(	18	40	10.7	28.7	1.42	0.0003	0.0022	**
siR46-4	TGAGATGGTAATAACGGT(	1362	2151	808.6	1543.5	0.93	0.0000	0.0000	
siR46-5	AATGGTATTCTAATATTGC	15	44	8.9	31.6	1.83	0.0000	0.0001	**

Genome-wide identification and characterization of phased small interfering RNA genes in response to Botrytis cinerea infection in Solanum lycopersicum
Weibo Jin, Yue Chen, Xiaole Zhu , Xin Tian, Fangli Wu

Table S5 Validation of 55 B.cinerea-responsive phasiRNAs using Weiberg's sRNA-seq data[25]

siRiRNA-id	Log2(TC7d/TD7d)Leaf	leaf0h-rp10m	leaf24h_rp10m	Log2(24h/0h)Leaf	Pvalue	Significance	leaf0h-rp10m	leaf72h_rp10m	Log2(72h/0h)Leaf	Pvalue	Significance	fruit0h-rp10m	fruit24h_rp10m	Log2(24h/0h)Fruit	Pvalue	Significance	fruit0h-rp10m	fruit72h_rp10m	Log2(72h/0h)Fruit	Pvalue	Significance
siR44-15	1.93	161.91	991.79	2.61	0.0000	**	161.91	1395.57	3.11	0.0000	**	281.43	699.29	1.31	0.0000	**	281.43	1180.04	2.07	0.0000	**
siR34-6	1.35	161.91	375.27	1.21	0.0000	**	161.91	986.52	2.61	0.0000	**	584.52	2055.07	1.81	0.0000	**	584.52	2932.21	2.33	0.0000	**
siR82-3	1.27	0.01	455.69	15.48	0.0000	**	0.01	144.37	13.82	0.0000	**	335.56	1512.76	2.17	0.0000	**	335.56	1287.31	1.94	0.0000	**
siR44-13	1.14	161.91	1849.56	3.51	0.0000	**	161.91	3344.55	4.37	0.0000	**	952.55	2311.95	1.28	0.0000	**	952.55	2038.25	1.10	0.0000	**
siR44-11	1.41	398.55	2332.06	2.55	0.0000	**	398.55	5197.29	3.70	0.0000	**	898.42	2526.03	1.49	0.0000	**	898.42	1483.99	0.72	0.0000	
siR31-3	1.18	12.46	53.61	2.11	0.0000	**	12.46	0.01	-10.28	0.0000	**	0.01	42.81	12.06	0.0000	**					
siR08-6	2.84	12.46	134.03	3.43	0.0000	**	12.46	48.12	1.95	0.0000	**	0.01	28.54	11.48	0.0000	**					
siR71-2	2.93	137.00	589.72	2.11	0.0000	**	137.00	769.97	2.49	0.0000	**	281.43	470.95	0.74	0.0000		281.43	1573.38	2.48	0.0000	**
siR13-8	2.02	12.46	53.61	2.11	0.0000	**	12.46	72.19	2.53	0.0000	**						0.01	17.88	10.80	0.0000	**
siR43-9	1.12	37.36	160.83	2.11	0.0000	**	37.36	409.05	3.45	0.0000	**	21.65	14.27	-0.60	0.0000		21.65	71.52	1.72	0.0000	**
siR23-4	1.93	49.82	187.64	1.91	0.0000	**	49.82	240.62	2.27	0.0000	**	64.95	71.36	0.14	0.0000		64.95	35.76	-0.86	0.0000	
siR27-8	1.93	49.82	187.64	1.91	0.0000	**	49.82	240.62	2.27	0.0000	**	64.95	71.36	0.14	0.0000		64.95	35.76	-0.86	0.0000	
siR69-1	1.93	49.82	187.64	1.91	0.0000	**	49.82	240.62	2.27	0.0000	**	64.95	71.36	0.14	0.0000		64.95	35.76	-0.86	0.0000	
siR15-6	1.26	74.73	241.25	1.69	0.0000	**	74.73	481.23	2.69	0.0000	**	140.72	142.71	0.02	0.0002		140.72	196.67	0.48	0.0000	
siR82-4	2.05	0.01	107.22	13.39	0.0000	**						75.77	585.12	2.95	0.0000	**	75.77	160.91	1.09	0.0000	**
siR52-10	1.22	274.00	321.66	0.23	0.0000		274.00	601.54	1.13	0.0000	**	519.57	1084.62	1.06	0.0000	**	519.57	1448.23	1.48	0.0000	**
siR33-14	1.21	37.36	53.61	0.52	0.0000		37.36	192.49	2.37	0.0000	**	21.65	256.88	3.57	0.0000	**	21.65	71.52	1.72	0.0000	**
siR08-3	1.04	24.91	26.80	0.11	0.0000		24.91	72.19	1.54	0.0000	**	0.01	28.54	11.48	0.0000	**	0.01	17.88	10.80	0.0000	**
siR06-2	1.87						0.01	24.06	11.23	0.0000	**	10.82	14.27	0.40	0.0000		10.82	107.28	3.31	0.0000	**
siR44-7	1.75						0.01	72.19	12.82	0.0000	**	10.82	85.63	2.98	0.0000	**	10.82	71.52	2.72	0.0000	**
siR43-7	1.13						0.01	24.06	11.23	0.0000	**						0.01	17.88	10.80	0.0000	**
siR07-2	1.63	921.65	2921.77	1.66	0.0000	**	921.65	794.03	-0.22	0.0000		573.69	2369.04	2.05	0.0000	**	573.69	375.47	-0.61	0.0000	
siR72-6	1.05	0.01	268.05	14.71	0.0000	**						140.72	356.78	1.34	0.0000	**	140.72	125.16	-0.17	0.0000	
siR41-10	2.26	24.91	53.61	1.11	0.0000	**	24.91	96.25	1.95	0.0000	**	10.82	0.01	-10.08	0.0000	**	10.82	0.01	-10.08	0.0000	**
siR16-1	1.42	74.73	160.83	1.11	0.0000	**	74.73	192.49	1.37	0.0000	**	108.24	99.90	-0.12	0.0000		108.24	53.64	-1.01	0.0000	**
siR52-11	1.08	946.56	3538.29	1.90	0.0000	**	946.56	4932.61	2.38	0.0000	**	2684.45	3881.80	0.53	0.0000		2684.45	1251.55	-1.10	0.0000	**
siR52-14	1.05	298.91	2600.11	3.12	0.0000	**	298.91	697.78	1.22	0.0000	**	681.94	770.65	0.18	0.0000		681.94	554.26	-0.30	0.0000	
siR43-4	2.54	24.91	53.61	1.11	0.0000	**	24.91	48.12	0.95	0.0000		21.65	0.01	-11.08	0.0000	**	21.65	53.64	1.31	0.0000	**
siR43-25	-1.80	12.46	0.01	-10.28	0.0000	**	12.46	0.01	-10.28	0.0000	**	10.82	14.27	0.40	0.0000		10.82	0.01	-10.08	0.0000	**
siR51-6	-1.35	62.27	0.01	-12.60	0.0000	**	62.27	72.19	0.21	0.0000											
siR37-3	1.39	62.27	26.80	-1.22	0.0000	**	62.27	216.55	1.80	0.0000	**	10.82	0.01	-10.08	0.0000	**	10.82	0.01	-10.08	0.0000	**
siR61-6	1.64	24.91	0.01	-11.28	0.0000	**	24.91	0.01	-11.28	0.0000	**	97.42	114.17	0.23	0.0000		97.42	53.64	-0.86	0.0000	
siR43-2	1.30	12.46	0.01	-10.28	0.0000	**	12.46	0.01	-10.28	0.0000	**										
siR40-15	2.02	373.64	80.42	-2.22	0.0000	**	373.64	409.05	0.13	0.0000											
siR43-19	1.42	74.73	0.01	-12.87	0.0000	**	74.73	48.12	-0.64	0.0000		10.82	0.01	-10.08	0.0000	**	10.82	0.01	-10.08	0.0000	**
siR39-3	1.11	286.46	455.69	0.67	0.0000		286.46	120.31	-1.25	0.0000	**	151.54	613.67	2.02	0.0000	**	151.54	71.52	-1.08	0.0000	**
siR26-2	-1.49	112.09	428.88	1.94	0.0000	**	112.09	384.98	1.78	0.0000	**	1428.82	485.23	-1.56	0.0000	**	1428.82	1019.12	-0.49	0.0000	
siR33-15	3.05	398.55	348.47	-0.19	0.0000		398.55	216.55	-0.88	0.0000		97.42	1070.35	3.46	0.0000	**	97.42	143.03	0.55	0.0000	
siR46-5	1.83	298.91	402.08	0.43	0.0000		298.91	505.29	0.76	0.0000		303.08	328.24	0.12	0.0000		303.08	53.64	-2.50	0.0000	**
siR43-5	1.43	348.73	268.05	-0.38	0.0000		348.73	673.72	0.95	0.0000		541.22	228.34	-1.25	0.0000	**	541.22	232.43	-1.22	0.0000	**
siR39-38	1.30	622.73	562.91	-0.15	0.0000		622.73	890.28	0.52	0.0000		616.99	884.82	0.52	0.0000		616.99	482.74	-0.35	0.0000	
siR55-4	1.29	87.18	53.61	-0.70	0.0000		87.18	48.12	-0.86	0.0000		64.95	128.44	0.98	0.0000		64.95	17.88	-1.86	0.0000	**
siR39-35	1.18	274.00	187.64	-0.55	0.0000		274.00	216.55	-0.34	0.0000		129.89	342.51	1.40	0.0000	**	129.89	89.40	-0.54	0.0000	
siR39-6	1.08	274.00	160.83	-0.77	0.0000		274.00	240.62	-0.19	0.0000		119.07	185.53	0.64	0.0000		119.07	214.55	0.85	0.0000	
siR40-19	1.08	846.92	428.88	-0.98	0.0000		846.92	769.97	-0.14	0.0000		10.82	14.27	0.40	0.0000		10.82	0.01	-10.08	0.0000	**
siR66-1	-2.00	24.91	26.80	0.11	0.0000		24.91	48.12	0.95	0.0000		108.24	0.01	-13.40	0.0000	**	108.24	143.03	0.40	0.0000	
siR67-4	-2.39	137.00	80.42	-0.77	0.0000		137.00	120.31	-0.19	0.0000		151.54	114.17	-0.41	0.0000		151.54	196.67	0.38	0.0000	
siR31-4	1.70																0.01	17.88	10.80	0.0000	**
siR60-1	1.05											0.01	14.27	10.48	0.0000	**					
siR38-14	7.89																				
siR16-5	3.22																				
siR40-13	1.91																				
siR23-3	1.90																				
siR55-1	1.53																				
siR46-1	1.42																				

Genome-wide identification and characterization of phased small interfering RNA genes in response to Botrytis cinerea infection in Solanum lycopersicum
Weibo Jin, Yue Chen, Xiaole Zhu , Xin Tian, Fangli Wu

Table S6 Targets prediction of *B.cinerea* -responsive phasiRNAs via psRNAtarget online

miRNA_Acc.	Target_Acc.	Expectation	UPE	miRNA_start	miRNA_end	Target_start	Target_end	miRNA_aligned_fragment	Target_aligned_fragment	Inhibition	Target_Desc.	**Degradome evidence**	
siR44-15	Solyc06g065340.1.1	2	12.308	1	21	174	194	UCAUGGAAUGAGAGUA	AUCAUCACUCUCAUUCCA	Cleavage	evidence_code:10F0H0E0I	**yes**	
siR44-15	Solyc02g091690.2.1	2.5	13.831	1	20	1116	1135	UCAUGGAAUGAGAGUA	UUGUUUCUCUUGUUCCAU	Cleavage	genomic_reference:SL2.50	**no**	
siR44-15	Solyc03g026230.1.1	3	11.79	1	20	234	253	UCAUGGAAUGAGAGUA	GCUUUACUCUCGUUCUAU	Cleavage	evidence_code:10F0H1E0I	**yes**	
siR34-6	Solyc12g006040.1.1	0	11.662	1	21	48	68	AAACAAGACAUAACGA	CCCGUUCGUUAUGUCUUG	Cleavage	evidence_code:10F0H1E1I	**yes**	
siR34-6	Solyc02g070310.2.1	2.5	8.326	1	21	553	573	AAACAAGACAUAACGA	CCUGUUCCUUGUGUUUUG	Cleavage	genomic_reference:SL2.50	**yes**	
siR34-6	Solyc08g013940.2.1	3	19.705	1	20	560	579	AAACAAGACAUAACGA	CUCUUGGUUGUGUCUUGU	Cleavage	genomic_reference:SL2.50	**yes**	
siR82-3	Solyc01g044500.1.1	3	20.289	1	20	259	278	ACAACUAGUGGACGAU	CAUGAUCGUUCAUUGGAU	Cleavage	evidence_code:10F0H0E0I	**yes**	
siR82-3	Solyc07g006620.2.1	3	13.807	1	20	586	605	ACAACUAGUGGACGAU	GAUAAUUGUUCACUAGUU	Cleavage	genomic_reference:SL2.50	**yes**	
siR82-3	Solyc09g092100.2.1	3	10.902	1	20	3439	3458	ACAACUAGUGGACGAU	CCUGAUCUUCCACUAGUU	Cleavage	genomic_reference:SL2.50	**no**	
siR82-3	Solyc01g006880.2.1	3	17.143	1	20	2813	2832	ACAACUAGUGGACGAU	CAUCAUCGCUUACUAGUU	Cleavage	genomic_reference:SL2.50	**no**	
siR44-13	Solyc12g007140.1.1	3	12.935	1	20	1942	1961	ACUUGUGACAUGUUAA	CCCGUUAACAUGUCAAGA	Cleavage	evidence_code:10F1H0E1I	**yes**	
siR44-11	Solyc06g074670.2.1	3	20.95	1	20	333	352	AGUUGUAGUCGGCUUA	CGAUUAAGCCGAUUACAA	Cleavage	genomic_reference:SL2.50	**yes**	
siR08-6	Solyc01g109410.2.1	3	15.244	1	21	339	359	AAUCAAUGGAAGCUCA	UCUUCUCACCUUCCAUUG	Cleavage	genomic_reference:SL2.50	**yes**	71
siR08-6	Solyc08g075150.2.1	3	20.425	1	21	915	935	AAUCAAUGGAAGCUCA	UCUGUUGAGCUUCAAUUG	Cleavage	genomic_reference:SL2.50	**yes**	
siR08-6	Solyc01g100390.2.1	3	19.316	1	21	1374	1394	AAUCAAUGGAAGCUCA	UCUUCUGUGUUUCAAUUG	Cleavage	genomic_reference:SL2.50	**yes**	
siR08-6	Solyc12g100360.1.1	3	21.447	1	20	2501	2520	AAUCAAUGGAAGCUCA	CUUCUUUGCUUCCAUUGA	Cleavage	evidence_code:10F0H1E1I	**yes**	
siR08-6	Solyc07g007280.2.1	3	6.587	1	20	103	122	AAUCAAUGGAAGCUCA	CUUCUUUGGUUCCAUUGA	Cleavage	genomic_reference:SL2.50	**yes**	
siR71-2	Solyc06g005410.2.1	0	15.571	1	21	324	344	UUUUCAAGCAACAUCU	CCUCUAGAUGUUGCUUGA	Cleavage	genomic_reference:SL2.50	**yes**	
siR71-2	Solyc09g014710.2.1	2.5	20.572	1	21	1554	1574	UUUUCAAGCAACAUCU	UCUUUGGAUGUUGUUUGC	Cleavage	genomic_reference:SL2.50	**yes**	
siR71-2	Solyc09g014740.2.1	2.5	19.377	1	21	1689	1709	UUUUCAAGCAACAUCU	UCUUUGGAUGUUGUUUGC	Cleavage	genomic_reference:SL2.50	**yes**	
siR71-2	Solyc06g072650.1.1	2	15.896	1	20	244	263	UUUUCAAGCAACAUCU	UUCAAGAUGUUGCUUGAA	Cleavage	evidence_code:10F1H0E1I	**yes**	
siR71-2	Solyc10g076550.1.1	3	18.149	1	21	806	826	UUUUCAAGCAACAUCU	UCUUUGGGUGUUGUUUGC	Cleavage	evidence_code:10F0H1E1I	**yes**	
siR71-2	Solyc10g076530.1.1	3	17.794	1	21	983	1003	UUUUCAAGCAACAUCU	UCUUUGGGUGUUGUUUGC	Cleavage	evidence_code:10F0H1E0I	**yes**	
siR71-2	Solyc09g014730.2.1	3	18.176	1	21	1473	1493	UUUUCAAGCAACAUCU	UCUUUGGGUGUUGUUUGC	Cleavage	genomic_reference:SL2.50	**yes**	
siR71-2	Solyc08g015780.2.1	3	12.581	1	21	314	334	UUUUCAAGCAACAUCU	CUUAUGGGUGUUGCUUGA	Cleavage	genomic_reference:SL2.50	**yes**	
siR71-2	Solyc02g094590.2.1	3	20.885	1	21	2044	2064	UUUUCAAGCAACAUCU	CUUUGAGAUGUUGUUUGA	Cleavage	genomic_reference:SL2.50	**no**	
siR71-2	Solyc05g015750.2.1	3	10.547	1	21	904	924	UUUUCAAGCAACAUCU	UUUCUGUAUGUUGUUUGA	Cleavage	genomic_reference:SL2.50	**yes**	
siR71-2	Solyc03g093990.2.1	3	23.878	1	21	1233	1253	UUUUCAAGCAACAUCU	CCUCUCUGUGUUGCUUGA	Cleavage	genomic_reference:SL2.50	**yes**	
siR71-2	Solyc01g091480.2.1	3	12.876	1	20	1551	1570	UUUUCAAGCAACAUCU	CUUAAGAUGUUGCUUCAA	Cleavage	genomic_reference:SL2.50	**yes**	
siR71-2	Solyc05g049970.2.1	3	17.602	1	20	1216	1234	UUUUCAAGCAACAUCU	CUGUAGAU-UUGCUUGAA	Cleavage	genomic_reference:SL2.50	**yes**	
siR13-8	Solyc02g055420.1.1	1.5	13.48	1	20	122	141	AUAUGAAGGUAAUGAU	AUCCAUUAUUAUUUUCAU	Cleavage	evidence_code:10F0H0E0I	**no**	
siR13-8	Solyc03g119240.2.1	3	8.635	1	21	163	183	AUAUGAAGGUAAUGAU	AAUCCAUUGUUGCCUUCC	Cleavage	genomic_reference:SL2.50	**no**	
siR13-8	Solyc01g065550.2.1	3	9.807	1	21	741	761	AUAUGAAGGUAAUGAU	GAUCCAUUGUUAUCUUCA	Cleavage	genomic_reference:SL2.50	**no**	
siR13-8	Solyc05g052630.2.1	2.5	13.034	1	20	2318	2337	AUAUGAAGGUAAUGAU	AUUCAAUAUUACCUUUAU	Cleavage	genomic_reference:SL2.50	**no**	
siR13-8	Solyc09g005080.1.1	3	13.844	1	20	614	633	AUAUGAAGGUAAUGAU	GUCAAUCUUUAUCUUCAU	Cleavage	evidence_code:10F1H1E1I	**no**	
siR23-4	Solyc11g020100.1.1	0	9.599	1	21	819	839	GUGGUUAAUAGAAUUC	AAGCCGAAUUCUAUUAAC	Cleavage	evidence_code:10F0H1E1I	**no**	
siR23-4	Solyc11g006630.1.1	0	13.05	1	21	831	851	GUGGUUAAUAGAAUUC	AAGCCGAAUUCUAUUAAC	Cleavage	evidence_code:10F0H1E1I	**no**	
siR23-4	Solyc11g006530.1.1	0	11.298	1	21	825	845	GUGGUUAAUAGAAUUC	AAGCCGAAUUCUAUUAAC	Cleavage	evidence_code:10F0H1E0I	**no**	
siR23-4	Solyc11g006520.1.1	0	14.042	1	21	813	833	GUGGUUAAUAGAAUUC	AAGCCGAAUUCUAUUAAC	Cleavage	evidence_code:10F0H1E1I	**no**	
siR23-4	Solyc10g051050.1.1	0.5	15.467	1	21	117	137	GUGGUUAAUAGAAUUC	AAGCCGAAUUCUAUUAAC	Cleavage	evidence_code:10F0H1E1I	**yes**	
siR23-4	Solyc11g006640.1.1	0.5	14.791	1	21	834	854	GUGGUUAAUAGAAUUC	AAGCCGAAUUCUAUUAAC	Cleavage	evidence_code:10F0H1E1I	**yes**	
siR23-4	Solyc12g017800.1.1	0.5	9.217	1	21	825	845	GUGGUUAAUAGAAUUC	AAGCCGAAUUCUGUUAAC	Cleavage	evidence_code:10F0H1E0I	**no**	
siR23-4	Solyc12g017730.1.1	1	11.913	1	21	135	155	GUGGUUAAUAGAAUUC	AAACCGAAUUCUAUUAAC	Cleavage	evidence_code:10F0H1E0I	**no**	
siR23-4	Solyc08g005500.2.1	2.5	17.71	1	21	849	869	GUGGUUAAUAGAAUUC	AAGCCGAAUACUGUUGAC	Cleavage	genomic_reference:SL2.50	**no**	
siR23-4	Solyc04g011890.1.1	3	22.909	1	21	150	170	GUGGUUAAUAGAAUUC	AAGUCGAAUCUUAUUGAC	Cleavage	evidence_code:10F0H1E0I	**no**	
siR23-4	Solyc07g014650.2.1	3	16.84	1	21	938	958	GUGGUUAAUAGAAUUC	AAGUGGAAUUUUAUUAGC	Cleavage	genomic_reference:SL2.50	**no**	
siR23-4	Solyc04g012010.2.1	3	19.793	1	21	857	877	GUGGUUAAUAGAAUUC	AAGUCGAAUCUUAUUGAC	Cleavage	genomic_reference:SL2.50	**no**	
siR23-4	Solyc02g027080.1.1	2.5	16.476	1	21	852	872	GUGGUUAAUAGAAUUC	GAGCCGAAUACUGUUGAC	Cleavage	evidence_code:10F0H1E1I	**no**	
siR23-4	Solyc01g016960.1.1	3	14.673	1	21	246	266	GUGGUUAAUAGAAUUC	GAGUCGAAUCUUAUUGAC	Cleavage	evidence_code:10F0H1E0I	**no**	
siR23-4	Solyc08g074250.1.1	3	18.623	1	21	870	890	GUGGUUAAUAGAAUUC	GAGUCGAAUCUUAUUGAC	Cleavage	evidence_code:10F0H1E1I	**no**	
siR23-4	Solyc04g011960.1.1	3	20.727	1	21	846	866	GUGGUUAAUAGAAUUC	GAGUCGAAUCUUAUUGAC	Cleavage	evidence_code:10F0H1E0I	**no**	
siR23-4	Solyc04g011990.1.1	3	19.756	1	21	837	857	GUGGUUAAUAGAAUUC	GAGUCGAAUCUUAUUGAC	Cleavage	evidence_code:10F0H1E0I	**no**	
siR27-8	Solyc11g020100.1.1	0	9.599	1	21	819	839	GUGGUUAAUAGAAUUC	AAGCCGAAUUCUAUUAAC	Cleavage	evidence_code:10F0H1E1I	**no**	
siR27-8	Solyc11g006630.1.1	0	13.05	1	21	831	851	GUGGUUAAUAGAAUUC	AAGCCGAAUUCUAUUAAC	Cleavage	evidence_code:10F0H1E1I	**no**	
siR27-8	Solyc11g006530.1.1	0	11.298	1	21	825	845	GUGGUUAAUAGAAUUC	AAGCCGAAUUCUAUUAAC	Cleavage	evidence_code:10F0H1E0I	**no**	
siR27-8	Solyc11g006520.1.1	0	14.042	1	21	813	833	GUGGUUAAUAGAAUUC	AAGCCGAAUUCUAUUAAC	Cleavage	evidence_code:10F0H1E1I	**no**	
siR27-8	Solyc10g051050.1.1	0.5	15.467	1	21	117	137	GUGGUUAAUAGAAUUC	AAGCCGAAUUCUAUUAAC	Cleavage	evidence_code:10F0H1E1I	**yes**	
siR27-8	Solyc11g006640.1.1	0.5	14.791	1	21	834	854	GUGGUUAAUAGAAUUC	AAGCCGAAUUCUAUUAAC	Cleavage	evidence_code:10F0H1E1I	**yes**	
siR27-8	Solyc12g017800.1.1	0.5	9.217	1	21	825	845	GUGGUUAAUAGAAUUC	AAGCCGAAUUCUGUUAAC	Cleavage	evidence_code:10F0H1E0I	**no**	
siR27-8	Solyc12g017730.1.1	1	11.913	1	21	135	155	GUGGUUAAUAGAAUUC	AAACCGAAUUCUAUUAAC	Cleavage	evidence_code:10F0H1E0I	**no**	
siR27-8	Solyc08g005500.2.1	2.5	17.71	1	21	849	869	GUGGUUAAUAGAAUUC	AAGCCGAAUACUGUUGAC	Cleavage	genomic_reference:SL2.50	**no**	
siR27-8	Solyc04g011890.1.1	3	22.909	1	21	150	170	GUGGUUAAUAGAAUUC	AAGUCGAAUCUUAUUGAC	Cleavage	evidence_code:10F0H1E0I	**no**	
siR27-8	Solyc07g014650.2.1	3	16.84	1	21	938	958	GUGGUUAAUAGAAUUC	AAGUGGAAUUUUAUUAGC	Cleavage	genomic_reference:SL2.50	**no**	
siR27-8	Solyc04g012010.2.1	3	19.793	1	21	857	877	GUGGUUAAUAGAAUUC	AAGUCGAAUCUUAUUGAC	Cleavage	genomic_reference:SL2.50	**no**	
siR27-8	Solyc02g027080.1.1	2.5	16.476	1	21	852	872	GUGGUUAAUAGAAUUC	GAGCCGAAUACUGUUGAC	Cleavage	evidence_code:10F0H1E1I	**no**	
siR27-8	Solyc01g016960.1.1	3	14.673	1	21	246	266	GUGGUUAAUAGAAUUC	GAGUCGAAUCUUAUUGAC	Cleavage	evidence_code:10F0H1E0I	**no**	
siR27-8	Solyc08g074250.1.1	3	18.623	1	21	870	890	GUGGUUAAUAGAAUUC	GAGUCGAAUCUUAUUGAC	Cleavage	evidence_code:10F0H1E1I	**no**	
siR27-8	Solyc04g011960.1.1	3	20.727	1	21	846	866	GUGGUUAAUAGAAUUC	GAGUCGAAUCUUAUUGAC	Cleavage	evidence_code:10F0H1E0I	**no**	
siR27-8	Solyc04g011990.1.1	3	19.756	1	21	837	857	GUGGUUAAUAGAAUUC	GAGUCGAAUCUUAUUGAC	Cleavage	evidence_code:10F0H1E0I	**no**	
siR69-1	Solyc11g020100.1.1	0	9.599	1	21	819	839	GUGGUUAAUAGAAUUC	AAGCCGAAUUCUAUUAAC	Cleavage	evidence_code:10F0H1E1I	**no**	
siR69-1	Solyc11g006630.1.1	0	13.05	1	21	831	851	GUGGUUAAUAGAAUUC	AAGCCGAAUUCUAUUAAC	Cleavage	evidence_code:10F0H1E1I	**no**	
siR69-1	Solyc11g006530.1.1	0	11.298	1	21	825	845	GUGGUUAAUAGAAUUC	AAGCCGAAUUCUAUUAAC	Cleavage	evidence_code:10F0H1E0I	**no**	
siR69-1	Solyc11g006520.1.1	0	14.042	1	21	813	833	GUGGUUAAUAGAAUUC	AAGCCGAAUUCUAUUAAC	Cleavage	evidence_code:10F0H1E1I	**no**	
siR69-1	Solyc10g051050.1.1	0.5	15.467	1	21	117	137	GUGGUUAAUAGAAUUC	AAGCCGAAUUCUAUUAAC	Cleavage	evidence_code:10F0H1E1I	**yes**	
siR69-1	Solyc11g006640.1.1	0.5	14.791	1	21	834	854	GUGGUUAAUAGAAUUC	AAGCCGAAUUCUAUUAAC	Cleavage	evidence_code:10F0H1E1I	**yes**	
siR69-1	Solyc12g017800.1.1	0.5	9.217	1	21	825	845	GUGGUUAAUAGAAUUC	AAGCCGAAUUCUGUUAAC	Cleavage	evidence_code:10F0H1E0I	**no**	
siR69-1	Solyc12g017730.1.1	1	11.913	1	21	135	155	GUGGUUAAUAGAAUUC	AAACCGAAUUCUAUUAAC	Cleavage	evidence_code:10F0H1E0I	**no**	
siR69-1	Solyc08g005500.2.1	2.5	17.71	1	21	849	869	GUGGUUAAUAGAAUUC	AAGCCGAAUACUGUUGAC	Cleavage	genomic_reference:SL2.50	**no**	
siR69-1	Solyc04g011890.1.1	3	22.909	1	21	150	170	GUGGUUAAUAGAAUUC	AAGUCGAAUCUUAUUGAC	Cleavage	evidence_code:10F0H1E0I	**no**	
siR69-1	Solyc07g014650.2.1	3	16.84	1	21	938	958	GUGGUUAAUAGAAUUC	AAGUGGAAUUUUAUUAGC	Cleavage	genomic_reference:SL2.50	**no**	
siR69-1	Solyc04g012010.2.1	3	19.793	1	21	857	877	GUGGUUAAUAGAAUUC	AAGUCGAAUCUUAUUGAC	Cleavage	genomic_reference:SL2.50	**no**	
siR69-1	Solyc02g027080.1.1	2.5	16.476	1	21	852	872	GUGGUUAAUAGAAUUC	GAGCCGAAUACUGUUGAC	Cleavage	evidence_code:10F0H1E1I	**no**	
siR69-1	Solyc01g016960.1.1	3	14.673	1	21	246	266	GUGGUUAAUAGAAUUC	GAGUCGAAUCUUAUUGAC	Cleavage	evidence_code:10F0H1E0I	**no**	
siR69-1	Solyc08g074250.1.1	3	18.623	1	21	870	890	GUGGUUAAUAGAAUUC	GAGUCGAAUCUUAUUGAC	Cleavage	evidence_code:10F0H1E1I	**no**	
siR69-1	Solyc04g011960.1.1	3	20.727	1	21	846	866	GUGGUUAAUAGAAUUC	GAGUCGAAUCUUAUUGAC	Cleavage	evidence_code:10F0H1E0I	**no**	
siR69-1	Solyc04g011990.1.1	3	19.756	1	21	837	857	GUGGUUAAUAGAAUUC	GAGUCGAAUCUUAUUGAC	Cleavage	evidence_code:10F0H1E0I	**no**	
siR15-6	Solyc06g054600.2.1	0	22.949	1	21	347	367	AGCUGGCUGUGGAUGG	UUUAACCAUCCACAGCCA	Cleavage	genomic_reference:SL2.50	**yes**	
siR15-6	Solyc06g054620.2.1	3	20.767	1	21	484	504	AGCUGGCUGUGGAUGG	UUUAACCAUCCACAACCA	Cleavage	genomic_reference:SL2.50	**yes**	
siR15-6	Solyc04g011460.1.1	3	20.003	1	20	137	156	AGCUGGCUGUGGAUGG	UUAACCAUUUACAGCCGC	Cleavage	evidence_code:10F1H1E1I	**no**	
siR15-6	Solyc06g082350.2.1	3	18.823	1	20	545	564	AGCUGGCUGUGGAUGG	UUCAAAAUCCACAGCCAG	Cleavage	genomic_reference:SL2.50	**yes**	
siR82-4	Solyc11g008530.1.1	0	19.05	1	21	160	180	CCAUGUAGCAGCAUUC	UACUGGAAUGCUGCUACA	Cleavage	evidence_code:10F0H1E1I	**yes**	
siR82-4	Solyc06g048960.2.1	1	24.02	1	21	404	424	CCAUGUAGCAGCAUUC	UAUUGGGAUGCUGCUACA	Cleavage	genomic_reference:SL2.50	**yes**	
siR82-4	Solyc11g008520.1.1	1.5	18.344	1	21	325	345	CCAUGUAGCAGCAUUC	UUCUGGGAUGCUGCUACA	Cleavage	evidence_code:10F0H1E0I	**yes**	
siR82-4	Solyc04g051550.1.1	2.5	22.902	1	21	3688	3708	CCAUGUAGCAGCAUUC	UACUGGAGUGCUGCUGCA	Cleavage	evidence_code:10F0H1E0I	**yes**	
siR82-4	Solyc03g007040.2.1	3	13.823	1	21	1418	1438	CCAUGUAGCAGCAUUC	UAUUGGAAUGUUGAUACA	Cleavage	genomic_reference:SL2.50	**yes**	
siR82-4	Solyc09g013130.2.1	2.5	22.116	1	20	1679	1698	CCAUGUAGCAGCAUUC	AUGGGAAUGUUGCUGCAU	Cleavage	genomic_reference:SL2.50	**yes**	
siR82-4	Solyc07g005170.1.1	3	11.976	1	20	920	939	CCAUGUAGCAGCAUUC	ACAGGGAUCCUGCUGCAU	Cleavage	evidence_code:10F0H1E0I	**yes**	
siR33-14	Solyc05g007850.1.1	0.5	15.6	1	21	759	779	UUAAUAUCAGCAAGAA	UUGUUUUCUUGCUGAUGU	Cleavage	evidence_code:10F0H1E0I	**yes**	
siR33-14	Solyc09g009320.1.1	2.5	23.335	1	21	435	455	UUAAUAUCAGCAAGAA	UUGUUUUGUUGUUGGUAU	Cleavage	evidence_code:10F0H0E0I	**yes**	
siR33-14	Solyc11g008690.1.1	2.5	23.107	1	20	414	433	UUAAUAUCAGCAAGAA	UGUUUUCGUGCUGAUAUU	Cleavage	evidence_code:10F0H1E0I	**no**	
siR33-14	Solyc00g006860.1.1	3	17.96	1	20	247	266	UUAAUAUCAGCAAGAA	GGUUUUAUUGUUGAUGUU	Cleavage	evidence_code:10F0H0E0I	**yes**	
siR33-14	Solyc06g063380.1.1	3	14.097	1	20	865	884	UUAAUAUCAGCAAGAA	GGUUUUAUUGUUGAUGUU	Cleavage	evidence_code:10F0H0E0I	**yes**	
siR33-14	Solyc05g052760.2.1	2.5	10.653	1	20	366	385	UUAAUAUCAGCAAGAA	UAUCUUUUUGCUGAUAUU	Cleavage	genomic_reference:SL2.50	**yes**	
siR08-3	Solyc12g013950.1.1	3	19.705	1	21	418	438	UCUGGGAGGCUCUUCA	GAUUAUGAGGAGCUUGCC	Cleavage	evidence_code:10F0H1E1I	**yes**	
siR08-3	Solyc09g010120.2.1	3	24.219	1	20	660	679	UCUGGGAGGCUCUUCA	AUCAUGAGAAGCCUCUUA	Cleavage	genomic_reference:SL2.50	**no**	
siR08-3	Solyc05g055470.2.1	3	19.912	1	20	643	662	UCUGGGAGGCUCUUCA	GUUAUGUAGAGUCUUCCA	Cleavage	genomic_reference:SL2.50	**yes**	
siR08-3	Solyc07g053020.1.1	3	16.006	1	20	664	683	UCUGGGAGGCUCUUCA	AUCAUGAAGAGUAUUCUC	Cleavage	evidence_code:10F0H1E1I	**yes**	
siR08-3	Solyc02g021000.2.1	3	13.702	1	20	6	25	UCUGGGAGGCUCUUCA	AUCCUCAAGAGCUUCUCA	Cleavage	genomic_reference:SL2.50	**yes**	
siR08-3	Solyc05g015420.2.1	3	16.448	1	20	368	387	UCUGGGAGGCUCUUCA	AUUAUCAAAAGCUUCCCA	Cleavage	genomic_reference:SL2.50	**yes**	
siR08-3	Solyc03g019850.2.1	3	17.307	1	20	702	721	UCUGGGAGGCUCUUCA	AUUAGGAAGAGCAUUCCA	Cleavage	genomic_reference:SL2.50	**yes**	
siR44-7	Solyc12g009520.1.1	0	20.357	1	21	242	262	GGUCCAAUCCGAUAAC	GUCACGUUAUCGGAUUGG	Cleavage	evidence_code:10F0H1E1I	**yes**	
siR44-7	Solyc12g013680.1.1	0	18.657	1	21	272	292	GGUCCAAUCCGAUAAC	GUCACGUUAUCGGAUUGG	Cleavage	evidence_code:10F0H1E1I	**yes**	
siR44-7	Solyc12g013730.1.1	2.5	23.283	1	21	11	31	GGUCCAAUCCGAUAAC	GUCAUGUUAUUGGACUGC	Cleavage	evidence_code:10F0H1E0I	**no**	
siR44-7	Solyc12g009750.1.1	3	16.249	1	21	119	139	GGUCCAAUCCGAUAAC	GUCAUGUUAUCGGUUUAC	Cleavage	evidence_code:10F0H1E0I	**yes**	
siR44-7	Solyc12g009730.1.1	3	16.249	1	21	119	139	GGUCCAAUCCGAUAAC	GUCAUGUUAUCGGUUUAC	Cleavage	evidence_code:10F0H1E0I	**yes**	
siR44-7	Solyc12g009770.1.1	3	17.621	1	21	110	130	GGUCCAAUCCGAUAAC	GUCAUGUUAUCGGUUUAC	Cleavage	evidence_code:10F0H1E0I	**yes**	
siR44-7	Solyc01g080110.2.1	2.5	12.726	1	20	1479	1498	GGUCCAAUCCGAUAAC	UCAUGUUCUUGGAUUGGA	Cleavage	genomic_reference:SL2.50	**yes**	
siR07-2	Solyc07g005040.2.1	2.5	13.604	1	21	95	115	AAAAAAGAAAGGUUGU	UUUGAAUAAUUUUUUUUU	Cleavage	genomic_reference:SL2.50	**yes**	
siR07-2	Solyc05g052100.2.1	3	17.441	1	21	1369	1389	AAAAAAGAAAGGUUGU	UUUGAACGAUCUUUCUUU	Cleavage	genomic_reference:SL2.50	**yes**	
siR07-2	Solyc11g066460.1.1	3	13.214	1	21	420	440	AAAAAAGAAAGGUUGU	UUUGGAUGAUUUUUCUUU	Cleavage	evidence_code:10F0H0E0I	**yes**	
siR07-2	Solyc07g038100.2.1	2.5	11.054	1	20	2224	2243	AAAAAAGAAAGGUUGU	GUAAGCAAUCUUUUUUUU	Cleavage	genomic_reference:SL2.50	**yes**	
siR07-2	Solyc01g008170.2.1	3	17.053	1	20	2217	2236	AAAAAAGAAAGGUUGU	GGCAACAACCUUUCUUUU	Cleavage	genomic_reference:SL2.50	**yes**	
siR07-2	Solyc04g015560.2.1	2	5.984	1	20	2006	2025	AAAAAAGAAAGGUUGU	UAAAACAACUUUUUUUUU	Cleavage	genomic_reference:SL2.50	**yes**	
siR07-2	Solyc12g049190.1.1	3	16.882	1	21	693	713	AAAAAAGAAAGGUUGU	UUUGACCAACCUUUCUAU	Cleavage	evidence_code:10F0H1E0I	**yes**	
siR07-2	Solyc04g081400.2.1	2.5	7.404	1	21	248	268	AAAAAAGAAAGGUUGU	UUCAAACAAACUUUUUUU	Cleavage	genomic_reference:SL2.50	**yes**	
siR07-2	Solyc04g050490.2.1	2.5	10.329	1	21	220	240	AAAAAAGAAAGGUUGU	UUUAAAACACUUUUCUUU	Cleavage	genomic_reference:SL2.50	**yes**	
siR07-2	Solyc03g082670.2.1	2.5	15.283	1	21	599	619	AAAAAAGAAAGGUUGU	UGUAAAGAACUUUUCUUU	Cleavage	genomic_reference:SL2.50	**yes**	
siR07-2	Solyc09g014920.1.1	2.5	8.084	1	21	33	53	AAAAAAGAAAGGUUGU	UAUAAAAAACCUUUUUUU	Cleavage	evidence_code:10F0H0E0I	**yes**	
siR07-2	Solyc01g008350.2.1	2.5	8.385	1	20	1523	1542	AAAAAAGAAAGGUUGU	UUCAAUAACUUUUUUUUU	Cleavage	genomic_reference:SL2.50	**yes**	

siR07-2	Solyc08g068300.2.1	2.5	7.161	1	20	106	125	AAAAAAGAAAGGUUGU	UAGAACAACUUUUUUUUU	Cleavage	genomic_reference:SL2.50	**yes**	13
siR07-2	Solyc01g006490.2.1	3	21.054	1	21	1975	1995	AAAAAAGAAAGGUUGU	UUUAUACCGCCUUUUUUU	Cleavage	genomic_reference:SL2.50	**yes**	
siR07-2	Solyc02g021440.2.1	3	12.386	1	21	194	214	AAAAAAGAAAGGUUGU	UUUAAAAACUCUUUUUUU	Cleavage	genomic_reference:SL2.50	**yes**	
siR07-2	Solyc07g065930.2.1	3	11.08	1	20	37	56	AAAAAAGAAAGGUUGU	AUAAAAAACCUUUCUUUU	Cleavage	genomic_reference:SL2.50	**yes**	
siR07-2	Solyc01g091370.2.1	3	9.153	1	20	53	72	AAAAAAGAAAGGUUGU	AUAAUUAACUUUUCUUUU	Cleavage	genomic_reference:SL2.50	**yes**	
siR07-2	Solyc07g041520.2.1	3	12.322	1	20	2268	2287	AAAAAAGAAAGGUUGU	UUCAACAACUUUUCUUUU	Cleavage	genomic_reference:SL2.50	**yes**	
siR07-2	Solyc07g006540.2.1	3	12.465	1	20	3430	3449	AAAAAAGAAAGGUUGU	UUAGAGAACCUUUAUUUU	Cleavage	genomic_reference:SL2.50	**yes**	
siR07-2	Solyc04g082770.2.1	3	9.045	1	20	24	43	AAAAAAGAAAGGUUGU	UUAAACAUCCUUUUCUUU	Cleavage	genomic_reference:SL2.50	**yes**	
siR07-2	Solyc01g102740.2.1	3	7.95	1	20	52	71	AAAAAAGAAAGGUUGU	UUAUACUAUCUUUUUUUU	Cleavage	genomic_reference:SL2.50	**yes**	
siR07-2	Solyc05g051570.2.1	3	16.066	1	20	1048	1067	AAAAAAGAAAGGUUGU	UUCAGGAAUCUUUCUUUU	Cleavage	genomic_reference:SL2.50	**yes**	
siR07-2	Solyc02g014720.2.1	3	11.978	1	20	1037	1056	AAAAAAGAAAGGUUGU	UGGAACAAACUUUUUUUU	Cleavage	genomic_reference:SL2.50	**yes**	
siR07-2	Solyc01g014820.1.1	3	10.692	1	20	178	197	AAAAAAGAAAGGUUGU	UUAAAGUGCCUUUUUUUU	Cleavage	evidence_code:10F0H1E0	**yes**	
siR07-2	Solyc04g082580.2.1	3	15.15	1	20	130	149	AAAAAAGAAAGGUUGU	UUAAAGAUGCUUUCUUUU	Cleavage	genomic_reference:SL2.50	**yes**	
siR72-6	Solyc11g008540.1.1	0	15.532	1	21	528	548	UUCGGAUUUGAGAAUG	UAUCCCAUUCUCAAAUCC	Cleavage	evidence_code:10F0H1E1	**no**	
siR72-6	Solyc11g008520.1.1	0.5	13.801	1	21	693	713	UUCGGAUUUGAGAAUG	UAUCCCGUUCUCAAAUCC	Cleavage	evidence_code:10F0H1E0	**no**	
siR72-6	Solyc11g008530.1.1	0.5	13.559	1	21	528	548	UUCGGAUUUGAGAAUG	UAUCCCAUUUUCAAAUCC	Cleavage	evidence_code:10F0H1E1	**no**	
siR72-6	Solyc08g080900.2.1	2	15.898	1	20	26	45	UUCGGAUUUGAGAAUG	AUUCCAUUCUCACAUCUG	Cleavage	genomic_reference:SL2.50	**yes**	
siR72-6	Solyc11g072320.1.1	3	16.097	1	21	1000	1020	UUCGGAUUUGAGAAUG	UACCUUAUUCUCAAAUCU	Cleavage	evidence_code:10F0H1E1	**yes**	
siR72-6	Solyc05g046280.1.1	3	13.351	1	20	1493	1512	UUCGGAUUUGAGAAUG	CUUUCAUUCUCAAAUCUG	Cleavage	evidence_code:10F0H1E0	**yes**	
siR72-6	Solyc05g008370.1.1	3	19.273	1	21	294	314	UUCGGAUUUGAGAAUG	UAUCCCUUUAUCAGAUCU	Cleavage	evidence_code:10F0H1E1	**yes**	
siR72-6	Solyc05g052840.1.1	2.5	7.728	1	20	233	252	UUCGGAUUUGAGAAUG	AUCCGACUCUUAAAUCCG	Cleavage	evidence_code:10F0H1E1	**yes**	
siR72-6	Solyc08g075550.2.1	3	15.566	1	21	666	686	UUCGGAUUUGAGAAUG	UAUCGAAUUAUCAAAUCC	Cleavage	genomic_reference:SL2.50	**no**	
siR43-25	Solyc05g008070.2.1	0	8.533	1	21	827	847	GUUAGUAUAAUUCGGC	UAAAAGCCGAAUUAUACU	Cleavage	genomic_reference:SL2.50	**no**	
siR43-25	Solyc07g049700.1.1	0.5	8.68	1	21	843	863	GUUAGUAUAAUUCGGC	UAAAAGCCGAAUUAUAUU	Cleavage	evidence_code:10F0H1E1	**no**	
siR43-25	Solyc10g051050.1.1	1.5	15.007	1	21	114	134	GUUAGUAUAAUUCGGC	UAAAAGCCGAAUUCUAUU	Cleavage	evidence_code:10F0H1E1	**yes**	
siR43-25	Solyc11g020100.1.1	1.5	10.674	1	21	816	836	GUUAGUAUAAUUCGGC	UAAAAGCCGAAUUCUAUU	Cleavage	evidence_code:10F0H1E1	**yes**	
siR43-25	Solyc11g006640.1.1	1.5	14.818	1	21	831	851	GUUAGUAUAAUUCGGC	UAAAAGCCGAAUUCUAUU	Cleavage	evidence_code:10F0H1E1	**yes**	
siR43-25	Solyc11g006530.1.1	1.5	12.59	1	21	822	842	GUUAGUAUAAUUCGGC	UAAAAGCCGAAUUCUAUU	Cleavage	evidence_code:10F0H1E0	**yes**	
siR43-25	Solyc11g006520.1.1	1.5	14.002	1	21	810	830	GUUAGUAUAAUUCGGC	UAAAAGCCGAAUUCUAUU	Cleavage	evidence_code:10F0H1E1	**yes**	
siR43-25	Solyc11g006630.1.1	2	14.41	1	21	828	848	GUUAGUAUAAUUCGGC	UAGAAGCCGAAUUCUAUU	Cleavage	evidence_code:10F0H1E1	**yes**	
siR43-25	Solyc12g017800.1.1	2.5	11.166	1	21	822	842	GUUAGUAUAAUUCGGC	UAGAAGCCGAAUUCUGUU	Cleavage	evidence_code:10F0H1E0	**yes**	
siR43-25	Solyc12g017730.1.1	3	11.931	1	21	132	152	GUUAGUAUAAUUCGGC	UAGAAACCGAAUUCUAUU	Cleavage	evidence_code:10F0H1E0	**yes**	
siR43-25	Solyc11g070170.1.1	3	11.665	1	20	1169	1188	GUUAGUAUAAUUCGGC	AAGAGCCUAAUUAUGCUA	Cleavage	evidence_code:10F0H1E1	**yes**	
siR43-25	Solyc11g072310.1.1	3	16.799	1	20	143	162	GUUAGUAUAAUUCGGC	AGAAGCCAAAUUAUGCUA	Cleavage	evidence_code:10F1H1E1	**yes**	
siR43-25	Solyc04g015220.2.1	3	18.397	1	20	679	698	GUUAGUAUAAUUCGGC	AAAAGUCGAAUUUUGUUC	Cleavage	genomic_reference:SL2.50	**yes**	

Genome-wide identification and characterization of phased small interfering RNA genes in response to Botrytis cinerea infection in Solanum lycopersicum

Fangli Wu, Yue Chen, Xing Tian, Xiaole Zhu, Weibo Jin

Table S7 Primers used in this study.

name	Sequence		Annealing temp
RT-PCR for 91 PHAS genes			
Sly-PHAS01	F	TTGTCATCCCGGACATTCT	53
	R	TGACTTCCACAAGCTGATTT	
Sly-PHAS02	F	GCAATAGCTTACCAAGGCGATGA	55
	R	TCTACGCATTTCACCGCTCCA	
Sly-PHAS03	F	GGGAGGTATTGGCAAAACTA	50
	R	CATACCGAGTACCGCATTTA	
Sly-PHAS04	F	GAAGGGAACCACCAGGAGTG	59
	R	AAGGGCAGGGACGTAGTCAA	
Sly-PHAS05	F	CGATCGGGGATAATCGTCGG	62
	R	GCCCAAGTCCCCTGAAACGG	
Sly-PHAS06	F	GTTCGCGGGTCGTTCTGTTG	60
	R	CTACGTCCCTGCCCTTTGCA	
Sly-PHAS07	F	GGTAAGACAACACTGACTGAGAAAA	55
	R	GAAGCCCCATTCCATATCTAAG	
Sly-PHAS08	F	AAGGAGTCGGTCTAACATTGGAAGG	58
	R	ATGCTTTCCCTCAGACGCAATATA	
Sly-PHAS09	F	GACTACAAGTCTCTAAACAAGAGAC	49
	R	GATCAGCAGATAAAACAGTGAGA	
Sly-PHAS10	F	CTTGAAATGACTCCACCGATTA	54
	R	AGCTGCGGAGACGACTATGT	
Sly-PHAS11	F	TGCTCTTGGGGCGCTTAGTA	60
	R	CGCAAAGGAGTTGCAGTGGT	
Sly-PHAS12	F	GAGAAATGGAGAACTTGCCTCT	52
	R	TTGGCATCAAGGTATCTACGA	
Sly-PHAS13	F	ATCAGAGCTGCTTTCCAAAA	55
	R	GATCTCATAGCGATCCGTGT	
Sly-PHAS14	F	CCGCTTCCCTCCCGACAATT	61
	R	GGTGAGATCCCCGTCGTTCC	
Sly-PHAS15	F	GCTGCGACCCTCTGTCTTCTT	57
	R	CCGACACGGGAACCACTCT	
Sly-PHAS16	F	ACGGGAACCACTTCAGGG	55
	R	TGTCTCCTTGTATCGCTATCTCC	
Sly-PHAS17	F	AGAGTGGTTTGGAATGGGTA	51
	R	CTTCAGCCGATCAATTATGTG	

Sly-PHAS18	F	CTTACCCTCCACCTTTTCAA	55
	R	TCCTGCGACAGTAGAGGAAT	
Sly-PHAS19	F	GCCCCCGACTATCTTTGTTAATC	55
	R	TCTCGTAGTTGGACTTTGGGATG	
Sly-PHAS20	F	GAAATTGGCTCAACCGACTTA	54
	R	GACCAAGAGGCTTCAGTAGACA	
Sly-PHAS21	F	CATCACTTCTACATTCGTGCTTGG	55
	R	AACTTCAGGCTCTGGTCCACACT	
Sly-PHAS22	F	GATTGGCAAAACGACTCTTG	55
	R	ATCTTTTCACGACATCCCAT	
Sly-PHAS23	F	AGTTTTACAAGTTGACGAGGACAT	55
	R	TGGTATGGGCGGTATCGGTA	
Sly-PHAS24	F	AAGTTTCCCCGTGTTGAGTC	55
	R	AACTGCGAAAGCATTTACCAA	
Sly-PHAS25	F	CGCTGGATAGTAGAAAGGCAC	54
	R	TGGGGACGGACAAGGAACGG	
Sly-PHAS26	F	GCCTTACGACCATACTCCCC	55
	R	GTCCTATTACATTGGCCTTCGGG	
Sly-PHAS27	F	CGCTGGACGATAAACTTGCT	54
	R	TAGAGGTCTGAAGGGTAGGAGA	
Sly-PHAS28	F	GTTGCCCTGGATGATGTG	50
	R	TCAATCAGCACCCTGGAA	
Sly-PHAS29	F	TGCGGGTAAGACAACACTAG	51
	R	CATTTCTCAGGTTATCCCACTC	
Sly-PHAS30	F	CAACCTCTGATTTGGAGCGTAAC	55
	R	TACCATGATGAGAGGGTGAAGAA	
Sly-PHAS31	F	CGATTATCTGTTGCTCCCATCT	55
	R	TGCAGCTACATACCACCTCACT	
Sly-PHAS32	F	GGACCAAAAGGATTTGAGAT	49
	R	AATAGACTTACCAGCCCCTT	
Sly-PHAS33	F	ATGGGGTATGGAAGGAGTAAGAA	54
	R	CAGTCTTCCAAACCATAAACGC	
Sly-PHAS34	F	AGGTGATGAGGCAAGTGATAGTA	54
	R	GGAATGGGTGGCATAGGTAAG	
Sly-PHAS35	F	CTTCAGCCGATCAATTATGTG	53
	R	CAGAGTGGTTTGGAATGGGTA	
Sly-PHAS36	F	TGGGACGGAGTTACTTGTGA	53
	R	GAAAGATCAAGGGAAACCAAA	
Sly-PHAS37	F	AGATTGTTGCTCATGGGATG	53
	R	ATTCTAAGATGGAAAGGTGG	
Sly-PHAS38	F	GGAATGGGCGGCTTGGGTAA	60
	R	CAATTTGGGCTAAATCGTTG	
Sly-PHAS39	F	GGGATGGATTACAACTTGAT	55

	R	ATGATATTGGGATAGGTCTTTC	
Sly-PHAS40	F	TACCAAAAGAAGGAGAAAGG	55
	R	CCACAAGCTCAGAAACAACT	
Sly-PHAS41	F	GGAATAAAATCAACAGCTCAGG	55
	R	GTCTTCCTAACGATGAGTGTCG	
Sly-PHAS42	F	TGTTTCCAAGTTGATTAGGCT	55
	R	ACTAAGGCATTGTTGGCGGA	
Sly-PHAS43	F	ACTGACGGACCTTCTTACCA	55
	R	GGGAGCTTTTGACCAACTAT	
Sly-PHAS44	F	GAGCTACCTCCTTCACTAACTG	55
	R	GCAACATGGATTTCCTTTGG	
Sly-PHAS45	F	AAACAACATATCATAGGACCCT	55
	R	AATTAACTTTTGCTGCCAAG	
Sly-PHAS46	F	AAAATCTTGAGCAATGAGTCAT	55
	R	GATTCCTCACTCTTTTCTTTTTG	
Sly-PHAS47	F	CTTTACCCTTCTTGTATCGG	55
	R	GATTGAATGCTAATGTACGTTG	
Sly-PHAS48	F	CTTTACCCAATGTATTGTCTGC	55
	R	GTTTTGAAATCCCTGATGCT	
Sly-PHAS49	F	TTTGGTTATAGTCCCTTTTGTC	55
	R	TTCCCTAACAATTTGTAGGTG	
Sly-PHAS50	F	TCCGTTCCTCTAATTGTTATCTC	55
	R	CCATTGGCTAAAGAATTGATGT	
Sly-PHAS51	F	CTTGACTCATTTGGTGGCATA	55
	R	TAGAATCGTCTTCTGGTGGG	
Sly-PHAS52	F	ATGTTTCTAATGGAGGCATAGTG	55
	R	TTCTTGACTCATTTGGTGGC	
Sly-PHAS53	F	ACAAGACACCTCCCTCAAAC	55
	R	AACGGAGACATTTCAGATATTTT	
Sly-PHAS54	F	CCAAGAGGATTTTCATTTTGA	55
	R	TGTGTTGGAAGAGTAGGGTAAG	
Sly-PHAS55	F	TTGTTGTCCTTGATGATGTGC	55
	R	ACTCATGATCCCTGTTTTCTAG	
Sly-PHAS56	F	TGTCATTGACCTGCATCTAGTC	55
	R	ATAATCAAATTGCCACTTGGC	
Sly-PHAS57	F	TATGGGAAAAGGGACAGATT	55
	R	GGATGGTTGTTTTATGGGTT	
Sly-PHAS58	F	CAAATTTGCAATCCCCATAC	55
	R	ATGCCAATCTTCTCACTCTGC	
Sly-PHAS59	F	CCCCATCACAATAAATTTTCA	55
	R	AGCTTCTTAGAGGGACTACAACT	
Sly-PHAS60	F	CAAGGAGCAGGATGCACAAA	55
	R	GGTCAATGAACTAAAGCGAGAA	

Sly-PHAS61	F	AGCCAGTACTTGTTGACCGA	55
	R	TTAGGAGCCCCCTTGACAA	
Sly-PHAS62	F	TCTTGAATCACTAACAATGCCT	55
	R	AATTTCATCAAATCCCAAGG	
Sly-PHAS63	F	CAATGTTAAGGCCACAGGAG	55
	R	CCCTTTTCTTTATGCTTTCTG	
Sly-PHAS64	F	GTGAAGTAGAAAGACAATCCATTG	55
	R	GTTCCTCTAATTGTGATCTGCC	
Sly-PHAS65	F	CTGTAATGCCATATCCTTCGA	55
	R	GACCGACAGTCAATTAACCAC	
Sly-PHAS66	F	CATCTTCATGTGAGGTGTATGG	55
	R	GCATCGAATATGTAGAGTTCCAC	
Sly-PHAS67	F	AATAATACTTGACAAGGTGGGT	55
	R	CAGGCTCGGACAAATAGA	
Sly-PHAS68	F	TTGGCTAATGCTGCAAAGCTCGA	55
	R	TGAAGCAAGGTCTGGAAATAGCACA	
Sly-PHAS69	F	TAATGGAGTTTGTGTACAGAGGTC	55
	R	TATGATCAGCTTCACTTAGGCAC	
Sly-PHAS70	F	TGAAACCAGATTGGAAAGGTATGAG	55
	R	TTATTGGACTTCTCCCCGTGTGAT	
Sly-PHAS71	F	TGCCTCAAATTCCTCAACCG	55
	R	GGAGATTCTGAAGCCCAACC	
Sly-PHAS72	F	TATTGTAGGAATGGGTGGTGC	55
	R	CGTTACCTGAACAAAACTGCTT	
Sly-PHAS73	F	ACCTTGCTTACAATGACTTTTATCC	55
	R	GGTTTAGTGTGTGGAGATGATGAAG	
Sly-PHAS74	F	ATTGATTGTCCCACTAAGTCTGTTG	55
	R	GGAAAAATTCAAGAGTTCAAGTCCA	
Sly-PHAS75	F	TCTATGGAATCTCACCAACATAGTG	55
	R	CAAGTAGTTTGATGACAACCAAAGT	
Sly-PHAS76	F	GTTGATGGAAGGCTCCTAAAAAG	55
	R	AAGCTATCGTGTGCTGGATTATG	
Sly-PHAS77	F	GACTTGCTATGAGGAGGAAAA	55
	R	CCTATATTCGTTCTCGATTTGA	
Sly-PHAS78	F	TTCAAGTCCAAAACATTAAGTGTCG	55
	R	GCTAATGACCCTTAAAGAGTTTCCA	
Sly-PHAS79	F	CCATTCGTTACGGTAGAGATAGAGT	55
	R	CCCAAAACACATACGGGAATAACAA	
Sly-PHAS80	F	AAACTGTGCATACCATTCGTTACAG	55
	R	CTTATTGTATCTAGCTTTCGTTGAG	
Sly-PHAS81	F	TGTGTATAGAAGTCTCAAGGGTAGG	55
	R	CTAATCTTACCACTCCTTTGACTTG	
Sly-PHAS82	F	GGATTCCCCTCCTATGTATCTGCCT	55

	R	GGCTCATTCGTGATCAATTTGAG	
Sly-PHAS83	F	AACTTCCAGTCTTCCCTATAATTGG	55
	R	GCGTTGCATGAACAACAGTAAACTA	
Sly-PHAS84	F	GTAGAGATTGTTGTTTATGGGATGG	55
	R	CGAGCCGTAGACTACCAGTAGCATA	
Sly-PHAS85	F	TCATAAAAAGCAGTCGTGTCGT	55
	R	GGAGCGGTAAAGAAGCCATC	
Sly-PHAS86	F	CTGAGCCATATGATGCTTTGAGAAT	55
	R	GAACAGAACTCCTTTACAAGACCAT	
Sly-PHAS87	F	TTACTACCAAAGGCAGTCAAGA	55
	R	TATTTCTACAAGTAAAGGAGGACAC	
Sly-PHAS88	F	ACAACTCTCGCTAACAAGCTATTTC	55
	R	CAGGACAACTGAAAGAGGTAGCC	
Sly-PHAS89	F	TATCCTAATGTTGTTGATCTTGTGA	55
	R	CCAACTCAAGCAACGGTTTAT	
Sly-PHAS90	F	CAGCCTGCCCACCCTGGAAT	55
	R	CGGCGACTTAGCCAACGACA	
Sly-PHAS91	F	CTTTGTCATCCCGGACATTC	55
	R	CATTGACTTCCACAAGCTGATTT	
Quantitative RT-PCR			
siR15-2	F	TATGAAAACAGGGCACTGTAA	60
siR15-6	F	AGCTGGCTGTGGATGGTTAAA	60
siR26-2	F	GCTTTTAGGACTCCGCCGGCA	60
siR26-4	F	TCTTGGATTTATGAAAGACGA	60
Ubi3-F		GCCGACTACAACATCCAGAAGG	60
Ubi3-R		TGCAACACAGCGAGCTTAACC	
